# Supplementary material for: ZEB1-mediated fibroblast polarization controls inflammation and sensitivity to immunotherapy in colorectal cancer
Source: EMBO Rep. 2024 Jun 27;25(8):15. doi: 10.1038/s44319-024-00186-7 (PMC11315988; doi:10.1038/s44319-024-00186-7)
Supplement: Supplementary file 1 — Appendix [file 44319_2024_186_MOESM1_ESM.pdf]

**Table of Contents**


| | |
|---|---|
| Appendix Fig. S1 | 2 |
| Appendix Fig. S2 | 4 |
| Appendix Fig. S3 | 6 |
| Appendix Fig. S4 | 7 |
| Appendix Table S1. Genotyping Primer sequences | 8 |
| Appendix Table S2. Antibodies | 9 |
| Appendix Table S3. qRT-PCR Primer sequences | 11 |

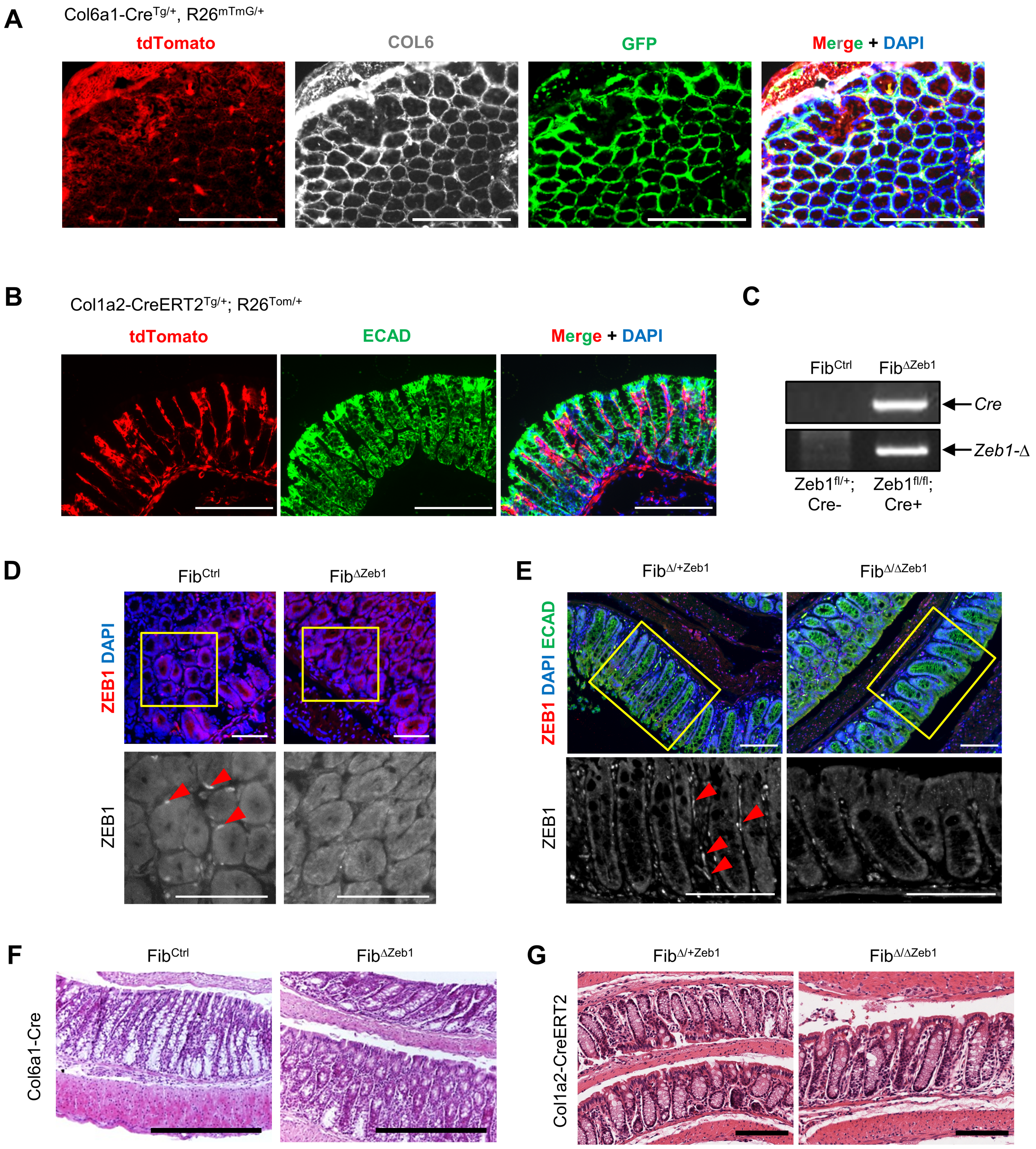

A
Col6a1-CreTg/+, R26mTmG/+
tdTomato
COL6
GFP
Merge + DAPI
B
Col1a2-CreERT2Tg/+; R26Tom/+
tdTomato
ECAD
Merge + DAPI
C
FibCtrl
FibΔZeb1
Cre
Zeb1-Δ
Zeb1fl/+; Cre-
Zeb1fl/fl; Cre+
D
FibCtrl
FibΔZeb1
ZEB1 DAPI
ZEB1
E
FibΔ/+Zeb1
FibΔ/ΔZeb1
ZEB1 DAPI ECAD
ZEB1
F
FibCtrl
FibΔZeb1
Col6a1-Cre
G
FibΔ/+Zeb1
FibΔ/ΔZeb1
Col1a2-CreERT2

**Appendix Fig. S1. Loss of *Zeb1* in fibroblasts cells does not affect intestinal homeostasis.**

(**A**) Col6 immunofluorescence (IF) and DAPI staining on colon cryosections of Col6a1-$Cre^{Tg/+}$;Rosa26-$mTmG^{Tg/+}$ mice. Overlay with tdTomato and GFP fluorescence. Scale bar: 200 µm.

(**B**) IF of tdTomato, E-cadherin and DAPI on colon sections of Col1a2-$CreERT^{Tg/+}$;Rosa26-$tdTomato^{Tg/+}$ mice. Scale bar: 200 µm.

(**C**) Genotyping of colon DNA confirms presence of Cre transgene and Col6a1-Cre induced recombination of *Zeb1* in indicated mouse genotypes.

(**D, E**) ZEB1 IF and DAPI staining on colon sections of $Fib^{Ctrl}$ ($Zeb1^{fl/fl}$;Col6a1-$Cre^{+/+}$) and $Fib^{\Delta Zeb1}$ ($Zeb1^{fl/fl}$;Col6a1-$Cre^{Tg/+}$) mice (**D**) and ZEB1/E-cadherin IF and DAPI staining on colon sections of heterozygous $Fib^{\Delta/+Zeb1}$ ($Zeb1^{fl/+}$;Col1a2-$CreERT2^{Tg/+}$) and $Fib^{\Delta Zeb1}$ ($Zeb1^{fl/fl}$;Col1a2-$CreERT2^{Tg/+}$) tamoxifen-fed mice (**E**). Bottom rows show magnification of ZEB1 of the marked areas in grayscale. Red arrowheads indicate ZEB1-positive stromal cells, which are absent in $Fib^{\Delta Zeb1}$. Scale bars: 100 µm.

(**F, G**) H&E stainings of colon sections from $Fib^{Ctrl}$ and $Fib^{\Delta Zeb1}$ mice (**F**), as well as of $Fib^{+/\Delta Zeb1}$ and $Fib^{\Delta Zeb1}$ tamoxifen-treated mice (**G**). Scale bars: 200 µm.

Data information: Scale bars represent 200 µm (A-B,F-G) or 100 µm (D-E).

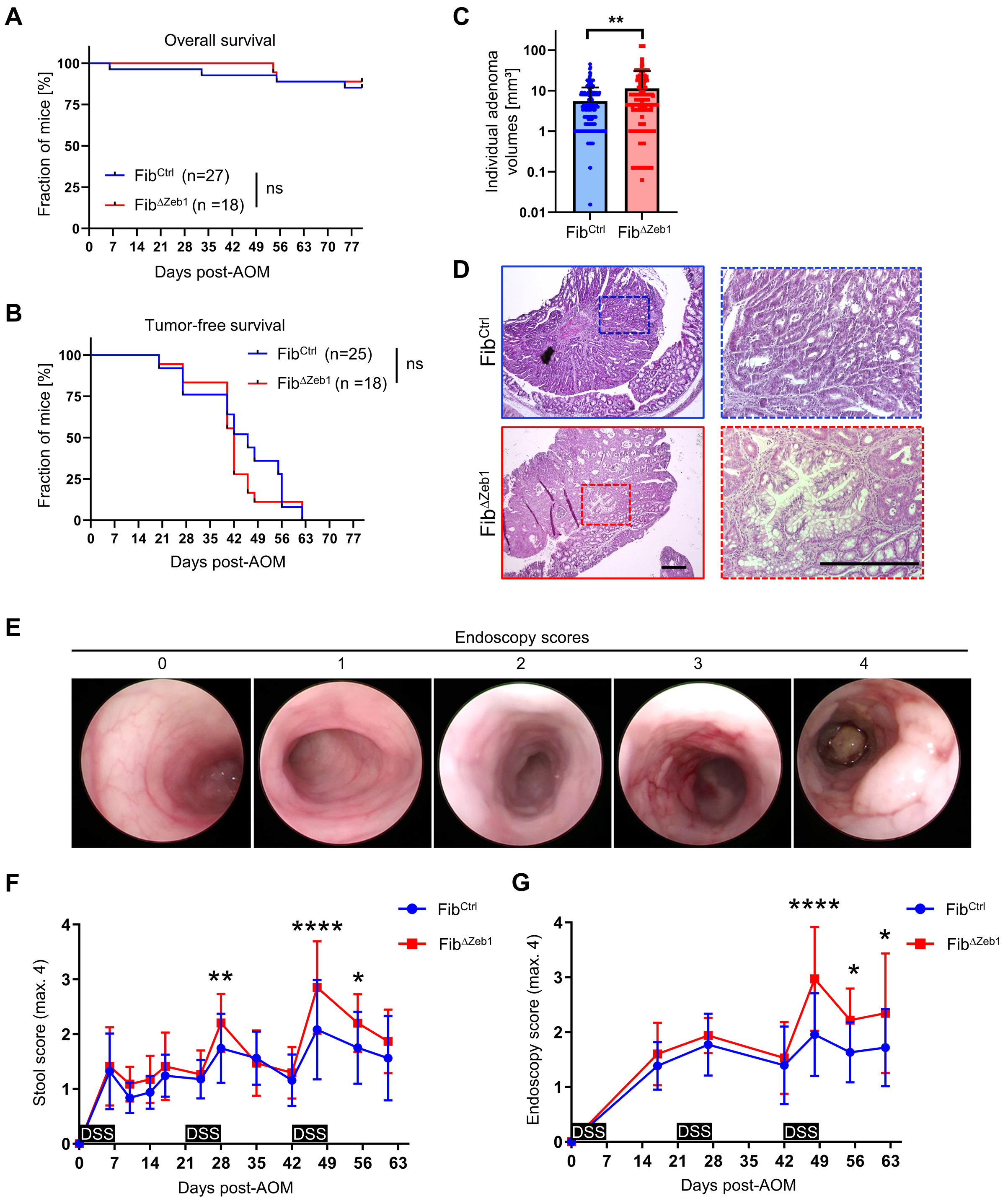
A
Overall survival
Fraction of mice [%]
Fib^Ctrl (n=27)
Fib^ΔZeb1 (n =18)
ns
Days post-AOM
B
Tumor-free survival
Fraction of mice [%]
Fib^Ctrl (n=25)
Fib^ΔZeb1 (n =18)
ns
Days post-AOM
C
**
Individual adenoma volumes [mm³]
Fib^Ctrl
Fib^ΔZeb1
D
Fib^Ctrl
Fib^ΔZeb1
E
Endoscopy scores
0
1
2
3
4
F
Stool score (max. 4)
DSS
Days post-AOM
Fib^Ctrl
Fib^ΔZeb1
G
Endoscopy score (max. 4)
DSS
Days post-AOM
Fib^Ctrl
Fib^ΔZeb1

**Appendix Fig. S2. ZEB1 in fibroblasts moderately exacerbates AOM/DSS-induced colitis.**

(**A, B**) Kaplan-Meier plots showing overall survival (**A**) and tumor-free survival (**B**) of $Fib^{Ctrl}$ and $Fib^{\Delta Zeb1}$ mice during AOM/DSS tumorigenesis. Numbers of experimental mice are indicated (Overall: p=0.7151, Tumor-free: p=0.3613, Mantel-Cox test).

(**C**) Quantification of volumes of individual adenomas after AOM/DSS tumorigenesis (n=204/207 for $Fib^{Ctrl}$/$Fib^{\Delta Zeb1}$ depicted on logarithmic scale and derived from 23/16 mice, p=0.0015, Mann-Whitney test).

(**D**) Representative images of H&E stainings of colon adenoma sections from $Fib^{Ctrl}$ and $Fib^{\Delta Zeb1}$ mice. Higher magnification of the indicated region is shown on the right.

(**E-G**) Representative endoscopic images (**E**), and associated stool (**F**) and endoscopy scores (**G**). Quantification of intestinal inflammation based on stool scoring (**F**) and on endoscopy scoring (**G**, also refer to images in E). n=25/17 (**F**) and n=25/18 (**G**) mice for $Fib^{Ctrl}$/$Fib^{\Delta Zeb1}$, Stool day 28, 47, 55: p= 0.0070, <00001, 0.0127, pUncorrected Fisher's LSD, Endoscopy day 48, 55, 62: p=<0.0001, 0.0343, 0.0195, Šídák's multiple comparisons test).

Data information: Data are presented as mean ± SD (C,F-G). Scale bars represent 300 µm (D).

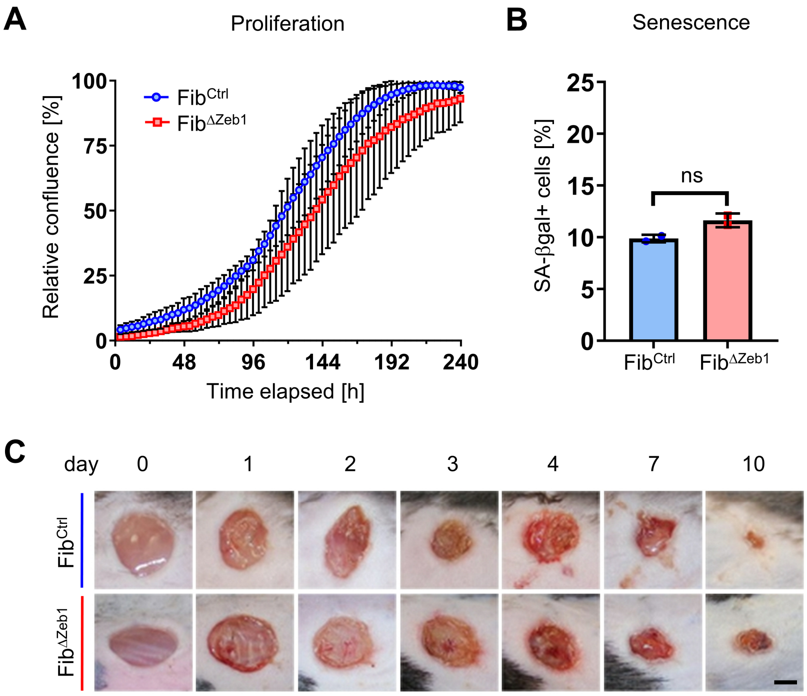


**Appendix Fig. S3. Effects of *Zeb1* loss in fibroblasts on proliferation, senescence and wound healing.**

(**A**) Proliferation assay by live-cell monitoring of cell confluence in $Fib^{Ctrl}$ and $Fib^{\Delta Zeb1}$ fibroblast cultures using IncuCyte (n=2/2 for $Fib^{Ctrl}/Fib^{\Delta Zeb1}$).

(**B**) Senescence-associated β-galactosidase (SA-β-gal) assay in cultured $Fib^{Ctrl}$ and $Fib^{\Delta Zeb1}$ fibroblasts (n=2/2 for $Fib^{Ctrl}/Fib^{\Delta Zeb1}$, p=0.0819, student's t-test).

(**C**) Representative images of the *in vivo* wound healing assay in $Fib^{Ctrl}$ and $Fib^{\Delta Zeb1}$ mice (n=16/22).

Data information: Data are presented as mean ± SD (A-B). Scale bars represent 2 mm (C).

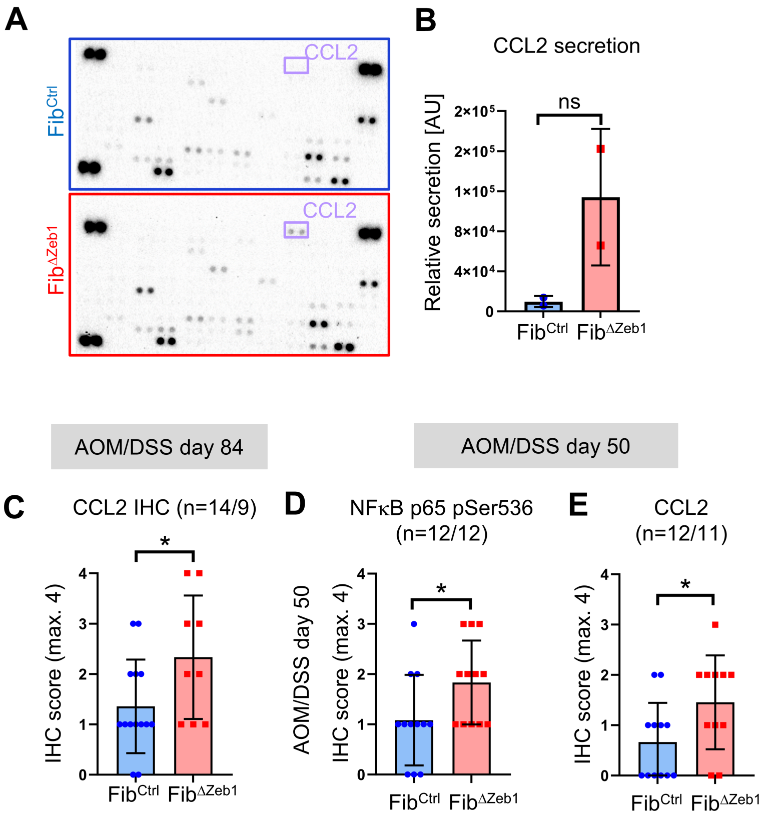


**Appendix Fig. S4. Increased CCL2 secretion in *Zeb1* deficient CAFs and IHC staining during AOM/DSS tumorigenesis.**

(**A, B**) Secretome analysis of $Fib^{Ctrl}$ and $Fib^{\Delta Zeb1}$ CAFs isolated from AOM/DSS tumors using a secretome array showing one representative exposure of an arrayed membrane (**A**) and quantification using two independent pairs of CAFs (p=0.1639, student's t-test) (**B**).

(**C**) IHC-based quantification of stromal CCL2 in tumors 84 days after AOM/DSS tumorigenesis. Numbers of experimental mice per genotype are indicated (p=0.0414, student's t-test)

(**D, E**) IHC-based quantification of phospho-NFκB p65 (Ser536) (**D**) and stromal CCL2 (**E**) in colons 50 days after AOM/DSS tumorigenesis. Numbers of experimental mice per genotype are indicated (p65: p=0.0459, Ccl2: p=0.0388, student's t-test).

Data information: Data are presented as mean ± SD (B-E).

## Appendix Table S1. Genotyping Primer sequences

| Target gene | Primer direction | Primer sequence |
|---|---|---|
| *Zeb1 floxed* | forward | CGTGATGGAGCCAGAATCTGACCCC |
| *Zeb1 floxed* | reverse | GGCCTGTCTTTCTCAGCAGTGTGG |
| *Zeb1 deleted* | reverse | GCCATCTCACCAGCCCTTACTGTGC |
| *Cre* | forward | TCCCCGCAGAACCTGAAGATGTTCG |
| *Cre* | reverse | GCCAGATTACGTATATCCTGGCAGC |
| *Col1a2-Cre* | forward | CAGGAGGTTTCGACTAAGTTGG |
| *Col1a2-Cre* | reverse | CATGTCCATCAGGTTCTTGC |
| *Col6a1-Cre* | forward | ACACACCGTAGCAACAGGAAGTC |
| *Col6a1-Cre* | reverse | TAGCTGGCCCAAATGTTGCT |
| *R26-mTmG* | forward | GTACTTGGCATATGATACACTTGATGTAC |
| *R26-mTmG* | reverse | AAAGTCGCTCTGAGTTGTTAT |
| *p53FRT* | forward | TCCCTTCCCTTATCCCTGTC |
| *p53FRT* | reverse | GGGTCACAGTATCCACAGAG |
| *FLP* | forward | GAGCCTGCAGTTCAAGTACAAGAC |
| *FLP* | reverse | GTTGTAAGGGATGATGGTGAACTC |
| *Gabra1 (internal DNA control)* | forward | AACACACACTGGAGGACTGGCTAGG |
| *Gabra1 (internal DNA control)* | reverse | CAATGGTAGGCTCACTCTGGGAGATGATA |

## Appendix Table S2. Antibodies

| Target antigen | used for | labeled | company | clone | catalogue number | working concentration | RRIDs |
|---|---|---|---|---|---|---|---|
| cl. CASP3 | IHC | unconjugated | Cell Signaling | rabbit polyclonal | 9661S | 1:200 dilution | Cell Signaling Technology Cat# 9661 (also NYUIHC-314, 9661S, 9661L), RRID:AB_2341188 |
| KI67 | IHC | unconjugated | abcam | SP6 | ab16667 | 1:300 dilution | Abcam Cat# ab16667, RRID:AB_302459 |
| CD4 | IHC | unconjugated | Sino Biol | Clone #1 | 50134-R001 | 1:500 dilution | Sino Biological Cat# 50134-R001, RRID:AB_2860490 |
| CD8$\alpha$ | IHC | unconjugated | Sino Biol | rabbit polyclonal | 50389-T26 | 1:500 dilution | - |
| FOXP3 | IHC | unconjugated | eBiosciences | FJK-16s | 14-5773-82 | 1:200 dilution | Thermo Fisher Scientific Cat# 14-5773-82, RRID:AB_467576 |
| PD-L1 | IHC | unconjugated | LSBio | rabbit polyclonal | LS-C19686 | 1:300 dilution | - |
| B220 | IHC | unconjugated | Thermo Fisher Scientific | RA3-6B2 | 14-0452-82 | 1:800 dilution | Thermo Fisher Scientific Cat# 14-0452-82, RRID:AB_467254 |
| MCP1 (CCL2) | IHC | unconjugated | Thermo Fisher Scientific | rabbit polyclonal | PA5-115555 | 1:200 dilution | Thermo Fisher Scientific Cat# PA5-115555, RRID:AB_2893318 |
| RELA/NFkB p-p65 (p-Ser536) | IHC | unconjugated | Novus | rabbit polyclonal | NB100-82088 | 1:100 dilution | Novus Cat# NB100-82088, RRID:AB_1144569 |
| ZEB1 | IHC | unconjugated | Novus | rabbit polyclonal | NBP1-05987 | 1:250 dilution | Novus Cat# NBP1-05987, RRID:AB_1556166 |
| CD4 | IHC | unconjugated | Cell Signaling | D7D2Z | 25229 | 1:100 dilution | Cell Signaling Technology Cat# 25229, RRID:AB_2798898 |
| FOXP3 | IHC | unconjugated | R&D Systems | 1054C | MAB8214 | 1:500 dilution | R and D Systems Cat# MAB8214, RRID:AB_2929004 |
| PD-L1 | IHC | unconjugated | Cell Signaling | E1L3N | 13684S | 1:200 dilution | - |
| CD8 | IHC | unconjugated | Cell Signaling | D4W2Z | 98941 | 1:500 dilution | Cell Signaling Technology Cat# 98941, RRID:AB_2756376 |
| F4/80 | IHC | unconjugated | Life Technologies | BM8 | MF48000 | 1:300 dilution | Thermo Fisher Scientific Cat# MF48000, RRID:AB_10376289 |
| F4/80 | IHC | unconjugated | Biorad | CI:A3-1 | MCA497 | 1:200 dilution | Bio-Rad Cat# MCA497, RRID:AB_2098196 |
| anti-rabbit-polymer | IHC | HRP | DAKO | | K4003 | | Agilent Cat# K4003 (also K4002), RRID:AB_2630375 |
| anti-rat-HRP | IHC | HRP | Life Technologies | rabbit polyclonal | A18915 | | Thermo Fisher Scientific Cat# A18915, RRID:AB_2535690 |
| ZEB1 | IF | unconjugated | Sigma | rabbit polyclonal | HPA027524 | 1:200 dilution | Sigma-Aldrich Cat# HPA027524, RRID:AB_1844977 |
| COL6 | IF | unconjugated | abcam | EPR17072 | ab182744 | 1:500 dilution | Abcam Cat# ab182744, RRID:AB_2847919 |
| ZEB1 | IF | unconjugated | Bethyl | rabbit polyclonal | IHC-00419 | 1:200 dilution | Bethyl Cat# IHC-00419 (also IHC-00419-T), RRID:AB_1659852 |
| E-cadherin | IF | unconjugated | BD Biosciences | 36 | 610181 | 1:200 dilution | BD Biosciences Cat# 610181, RRID:AB_397580 |
| $\alpha$SMA | IF | unconjugated | Thermo Fisher Scientific | 1A4 | 14-9760-82 | 1:200 dilution | Thermo Fisher Scientific Cat# 14-9760-82 (also 14-9760), RRID:AB_2572996 |
| anti-mouse | IF | Alexa Fluor 488 | Thermo Fisher Scientific | donkey polyclonal | A21202 | 1:500 dilution | Thermo Fisher Scientific Cat# A-11055, RRID:AB_2534102 |

| | | | | | | | |
|---|---|---|---|---|---|---|---|
| anti-rabbit | IF | Alexa Fluor 594 | Thermo Fisher Scientific | donkey polyclonal | A21207 | 1:500 dilution | Thermo Fisher Scientific Cat# A-21207, RRID:AB_141637 |
| anti-rabbit | IF | Alexa Fluor 647 | Thermo Fisher Scientific | donkey polyclonal | A31573 | 1:500 dilution | Thermo Fisher Scientific Cat# A-31571, RRID:AB_162542 |
| IκBα | Western blot | unconjugated | Cell Signaling | rabbit polyclonal | CS9242 | 1:5000 dilution | Cell Signaling Technology Cat# 9242, RRID:AB_331623 |
| NFκB p65 | Western blot | unconjugated | Cell Signaling | D14E12 | CS8242 | 1:2000 dilution | Cell Signaling Technology Cat# 8242, RRID:AB_10859369 |
| p-IκBα Ser32 | Western blot | unconjugated | Cell Signaling | 14D4 | CS2859 | 1:1000 dilution | Cell Signaling Technology Cat# 2859 (also 2859S, 2859P, 2859L), RRID:AB_561111 |
| p-NFκB p65 Ser536 | Western blot | unconjugated | Cell Signaling | 7F1 | CS3036 | 1:1000 dilution | Cell Signaling Technology Cat# 3036, RRID:AB_331281 |
| Zeb1 | Western blot | unconjugated | Sigma | rabbit poyclonal | HPA027524 | 1:2000 dilution | Sigma-Aldrich Cat# HPA027524, RRID:AB_1844977 |
| β-actin | Western blot | unconjugated | Sigma | AC-15 | A5441 | 1:20000 dilution | Sigma-Aldrich Cat# A5441, RRID:AB_476744 |
| anti-rabbit IgG-HRP | Western blot | unconjugated | Dianova | goat polyclonal | 111-035-144 | 1:10000 dilution | Jackson ImmunoResearch Labs Cat# 111-035-144, RRID:AB_2307391 |
| anti-mouse IgG-HRP | Western blot | unconjugated | Dianova | goat polyclonal | 115-035-146 | 1:10000 dilution | Jackson ImmunoResearch Labs Cat# 115-035-146, RRID:AB_2307392 |
| EPCAM | Phenoptics | unconjugated | Cell Signaling | E6V8Y | 93790S | 1:500 dilution | Cell Signaling Technology Cat# 93790, RRID:AB_2800214 |
| VIM | Phenoptics | unconjugated | abcam | EPR3776 | ab92547 | 1:100 dilution | Abcam Cat# ab92547, RRID:AB_10562134 |
| αSMA | Phenoptics | unconjugated | Sigma | 1A4 | F3777 | 1:5000 dilution | Sigma-Aldrich Cat# F3777, RRID:AB_476977 |
| C3 | Phenoptics | unconjugated | abcam | EPR19394 | ab200999 | 1:2000 dilution | Abcam Cat# ab200999, RRID:AB_2924273 |
| MHCII | Phenoptics | unconjugated | Invitrogen | M5/114.15.2 | 14-5321-82 | 1:400 dilution | Thermo Fisher Scientific Cat# 14-5321-82, RRID:AB_467561 |
| CD45 | Phenoptics | unconjugated | Cell Signaling | D3F8Q | 70257 | 1:500 dilution | Cell Signaling Technology Cat# 70257, RRID:AB_2799780 |
| CD16/CD32 | FACS | unconjugated | BD Biosciences | 2.4G2 | 553142 | 1:100 dilution | BD Biosciences Cat# 553141, RRID:AB_394656 |
| CD45 | FACS | PE/Cy7 | BioLegend | 30-F11 | 103113 | 1:100 dilution | BioLegend Cat# 103114 (also 103113), RRID:AB_312979 |
| CD31 | FACS | PE | Thermo Fisher Scientific | 390 | 12-0311-82 | 1:100 dilution | Thermo Fisher Scientific Cat# 12-0311-82, RRID:AB_465632 |
| EPCAM | FACS | eFluor450 | Thermo Fisher Scientific | G8.8 | 48-5791-82 | 1:100 dilution | Thermo Fisher Scientific Cat# 48-5791-82, RRID:AB_10717090 |
| CD140a | FACS | APC | Thermo Fisher Scientific | APA5 | 17-1401-81 | 1:100 dilution | Thermo Fisher Scientific Cat# 17-1401-81, RRID:AB_529482 |

## Appendix Table S3. qRT-PCR Primer sequences

| Target mRNA | primer direction | primer sequence | UPL |
|---|---|---|---|
| *Zeb1* | forward | AGGTGATCCAGCCAAACG | 93 |
| *Zeb1* | reverse | AGGCCTGACATGTAGTCTTGTG | 93 |
| *Acta2* | forward | CTCTCTTCCAGCCATCTTTCAT | 58 |
| *Acta2* | reverse | TATAGGTGGTTTCGTGGATGC | 58 |
| *Gapdh* | forward | GGGTTCCTATAAATACGGACTGC | 52 |
| *Gapdh* | reverse | CCATTTTGTCTACGGGACGA | 52 |
| *Gapdh #2* | forward | AGCTTGTCATCAACGGGAAG | 9 |
| *Gapdh #2* | reverse | TTTGATGTTAGTGGGGTCTCG | 9 |
| *Pdgfrb* | forward | TCAAGCTGCAGGTCAATGTC | 67 |
| *Pdgfrb* | reverse | CCATTGGCAGGGTGACTC | 67 |
| *Ccl2* | forward | CCAATGAGTAGGCTGGAGAGC | 62 |
| *Ccl2* | reverse | CCCATTCCTTCTTGGGGTCA | 62 |
| *Cxcl1* | forward | GACTCCAGCCACACTCCAAC | 83 |
| *Cxcl1* | reverse | TGACAGCGCAGCTCATTG | 83 |
| *Hprt* | forward | AAGCTTGCTGGTGAAAAGGA | - |
| *Hprt* | reverse | TTGCGCTCATCTTAGGCTTT | - |
| *Tagln* | forward | CGGCCTTTAAACCCCTCACC | - |
| *Tagln* | reverse | GACTGCACTTCTCGGCTCAT | - |
| *Acta2 #2* | forward | AGGGCTGTTTTCCCATCCAT | - |
| *Acta2 #2* | reverse | GGCCCATTCCAACCATTACTC | - |
| *Ccl2 #2* | forward | AGCCTACTCATTGGGATCATCTTG | - |
| *Ccl2 #2* | reverse | CAGCCAGATGCAGTTAACGC | - |
| *Cxcl1 #2* | forward | ACTGCACCCAAACCGAAGTC | - |
| *Cxcl1 #2* | reverse | TGGGGACACCTTTTAGCATCTT | - |
