# Supplementary figures and images for: ZEB1-mediated fibroblast polarization controls inflammation and sensitivity to immunotherapy in colorectal cancer
Source: EMBO Rep. 2024 Jun 27;25(8):15. doi: 10.1038/s44319-024-00186-7 (PMC11315988; doi:10.1038/s44319-024-00186-7)

## Slide 1
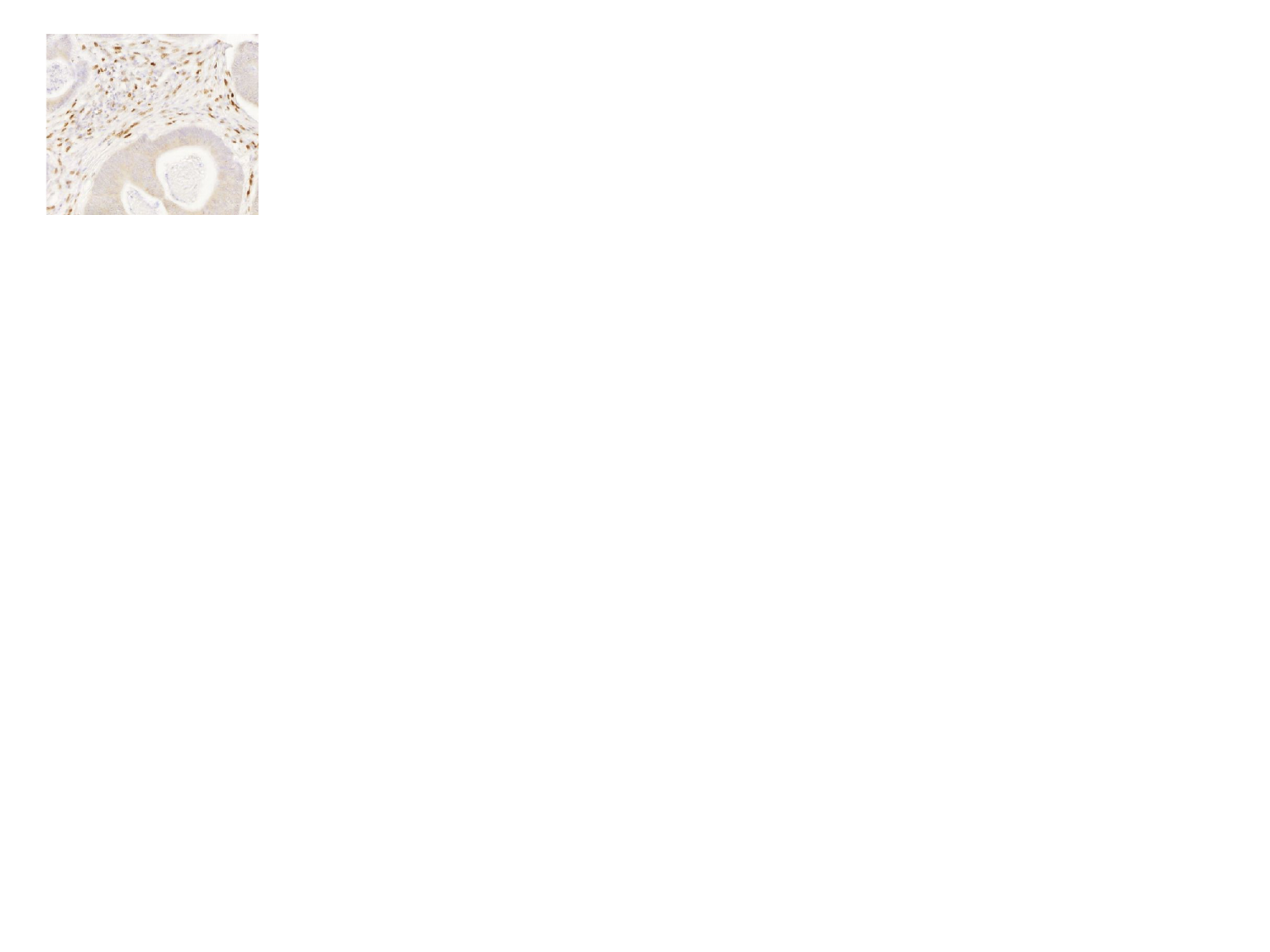

Supplement: Supplementary file 3 — Source data Fig. 1 [file 44319_2024_186_MOESM3_ESM.zip › EMBOR-2024-59433V3 Fig. 1 Source Data/1A/Fig. 1A image data.pptx]

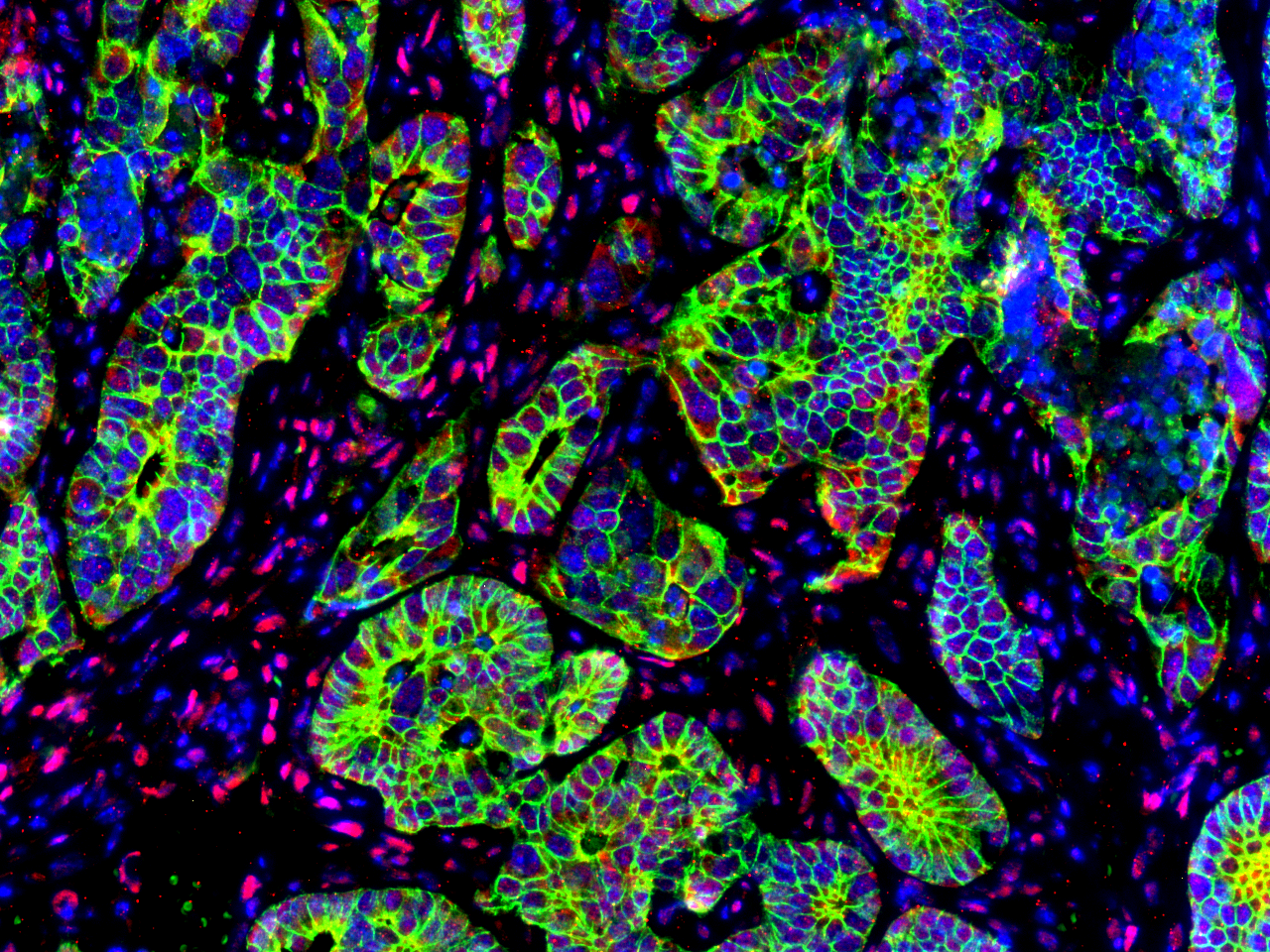

Supplement: Supplementary file 3 — Source data Fig. 1 [file 44319_2024_186_MOESM3_ESM.zip › EMBOR-2024-59433V3 Fig. 1 Source Data/1B/mouse IF image.tif]

## Slide 1
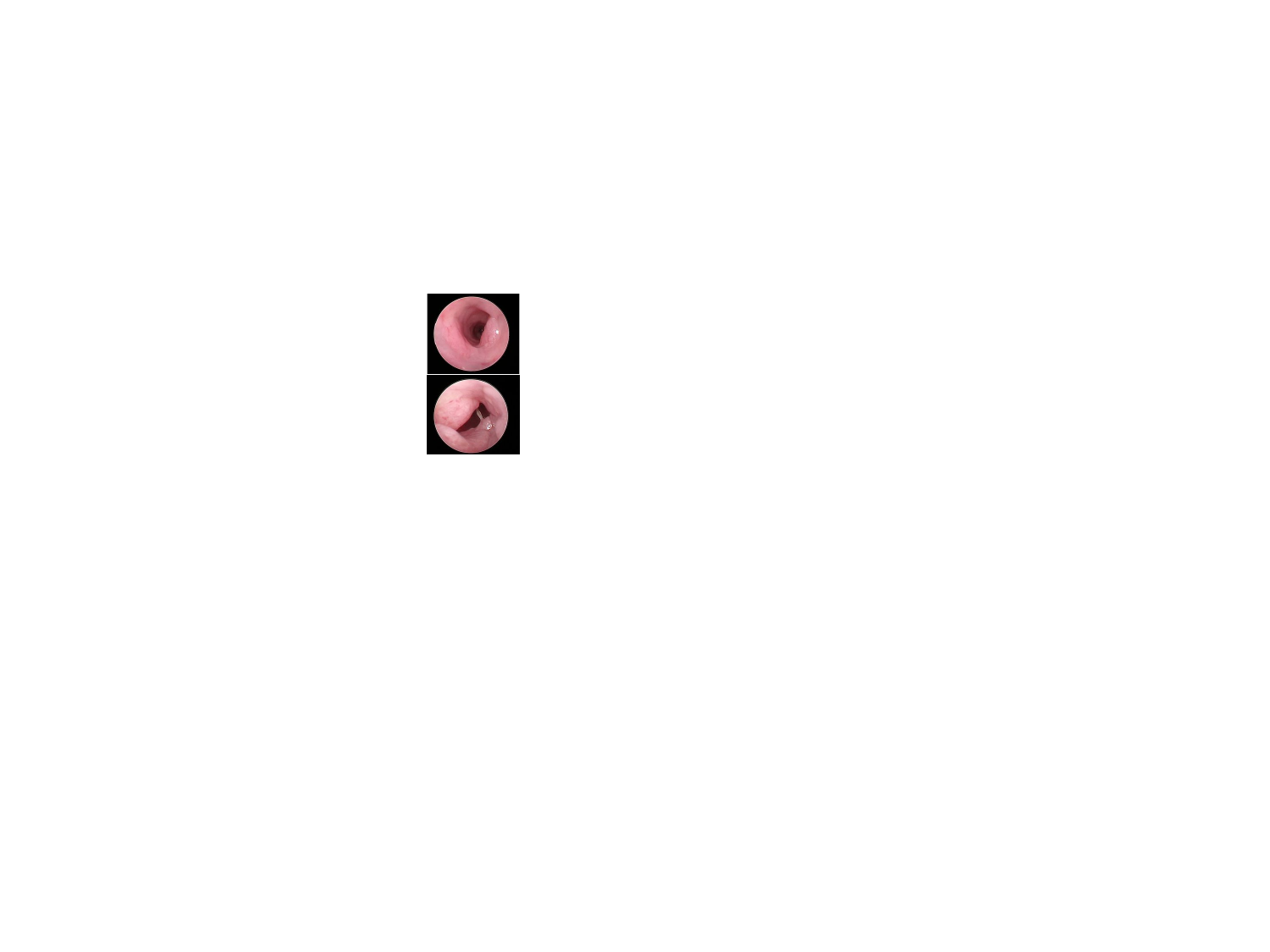

Supplement: Supplementary file 3 — Source data Fig. 1 [file 44319_2024_186_MOESM3_ESM.zip › EMBOR-2024-59433V3 Fig. 1 Source Data/1D/Fig. 1D image data.pptx]

## Slide 1
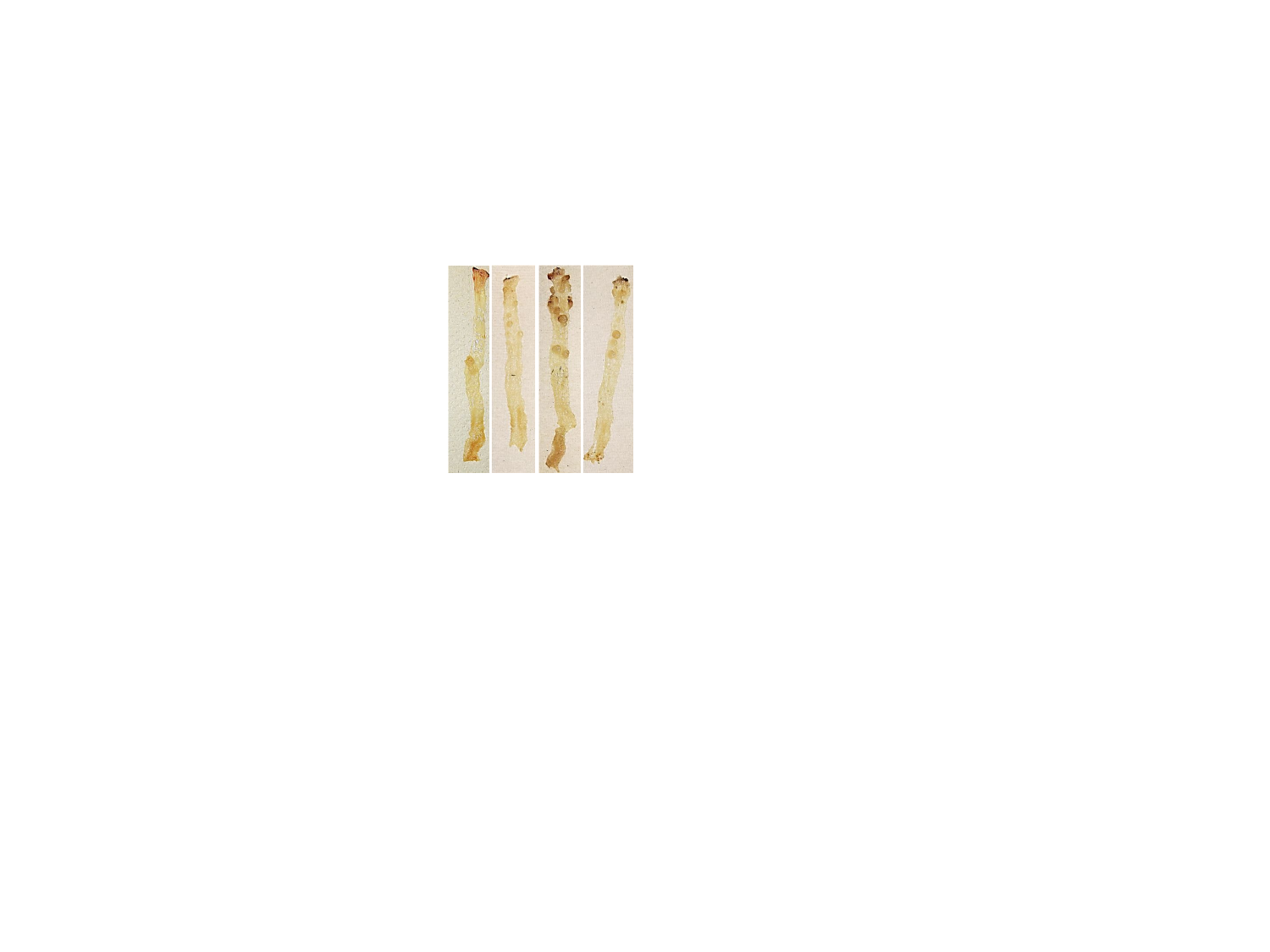

Supplement: Supplementary file 3 — Source data Fig. 1 [file 44319_2024_186_MOESM3_ESM.zip › EMBOR-2024-59433V3 Fig. 1 Source Data/1E/Fig. 1E image data.pptx]

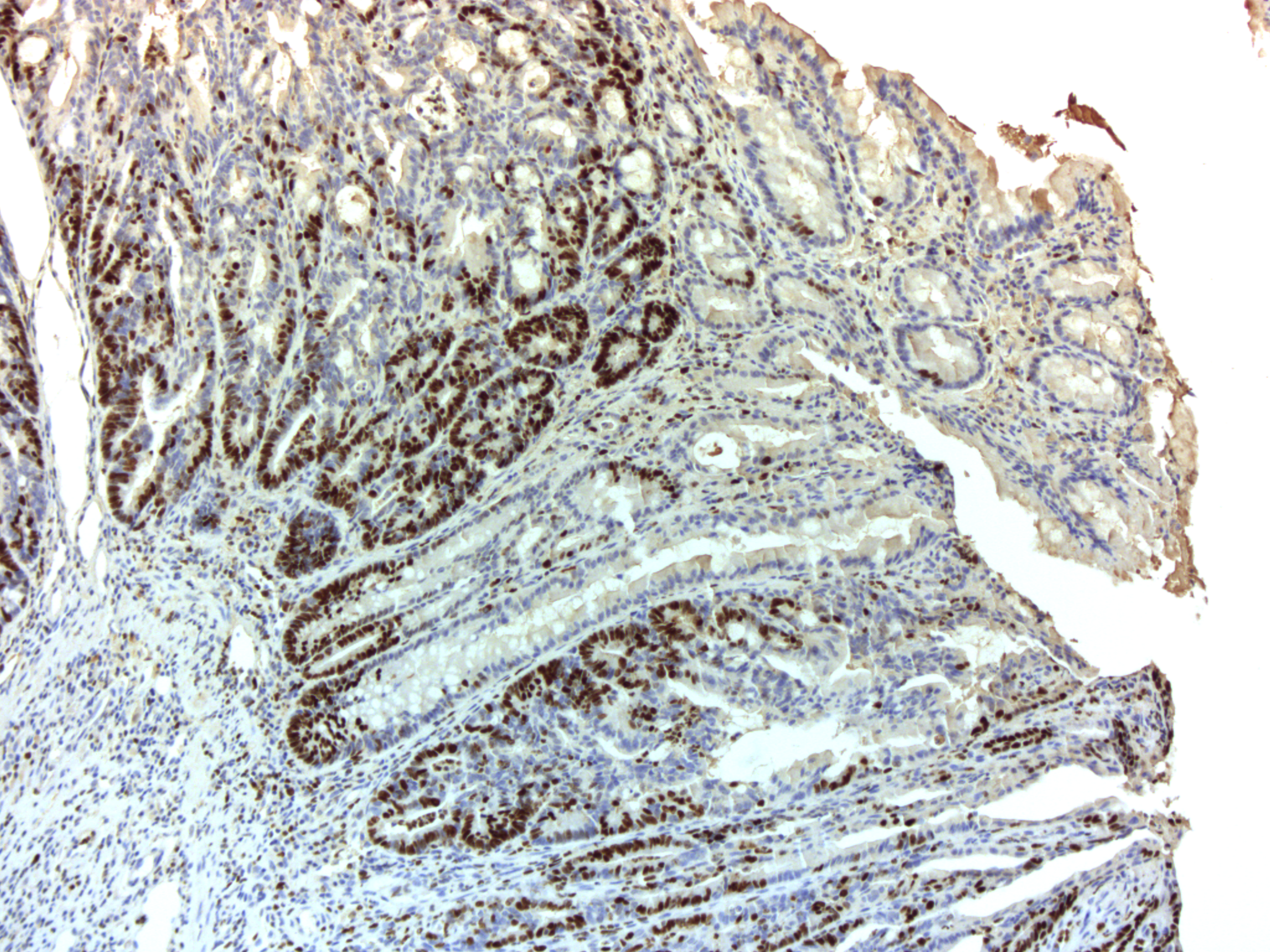

Supplement: Supplementary file 3 — Source data Fig. 1 [file 44319_2024_186_MOESM3_ESM.zip › EMBOR-2024-59433V3 Fig. 1 Source Data/1H/IHC Ctrl Ki67.tif]

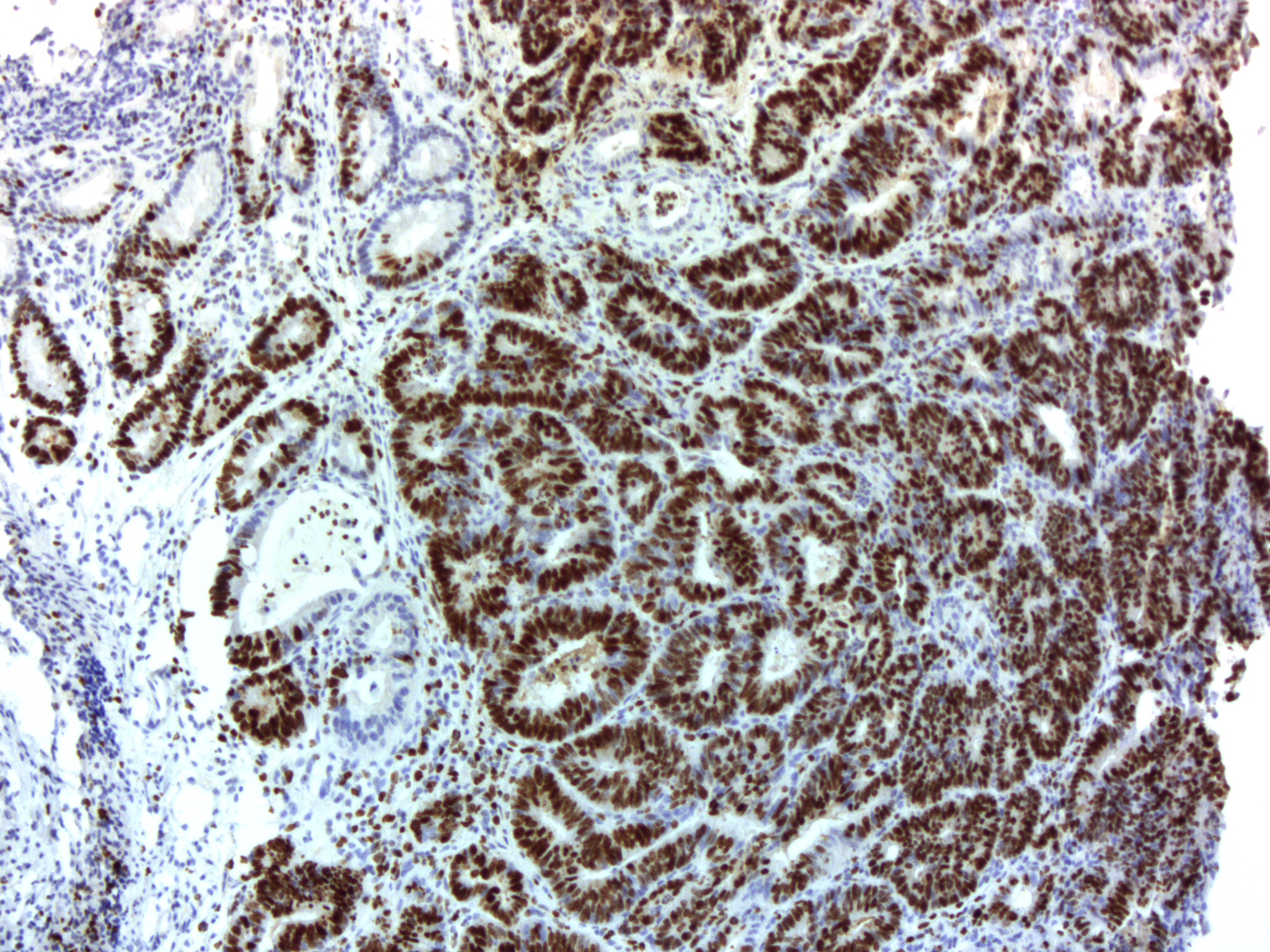

Supplement: Supplementary file 3 — Source data Fig. 1 [file 44319_2024_186_MOESM3_ESM.zip › EMBOR-2024-59433V3 Fig. 1 Source Data/1H/IHC Del Ki67.tif]

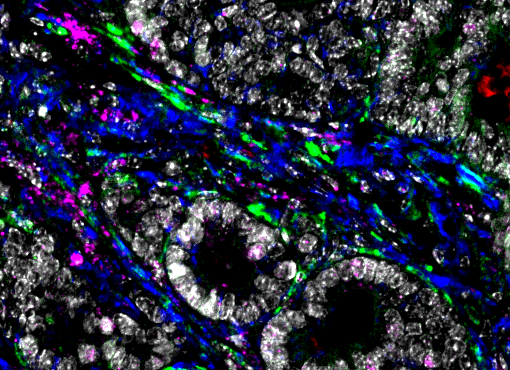

Supplement: Supplementary file 4 — Source data Fig. 2 [file 44319_2024_186_MOESM4_ESM.zip › EMBOR-2024-59433V3 Fig. 2 Source Data/2K/Ctrl IF image.tif]

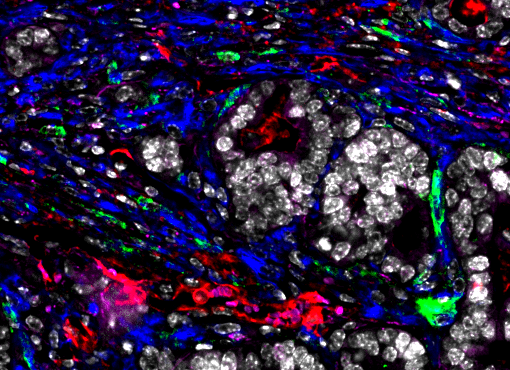

Supplement: Supplementary file 4 — Source data Fig. 2 [file 44319_2024_186_MOESM4_ESM.zip › EMBOR-2024-59433V3 Fig. 2 Source Data/2K/Del IF image.tif]

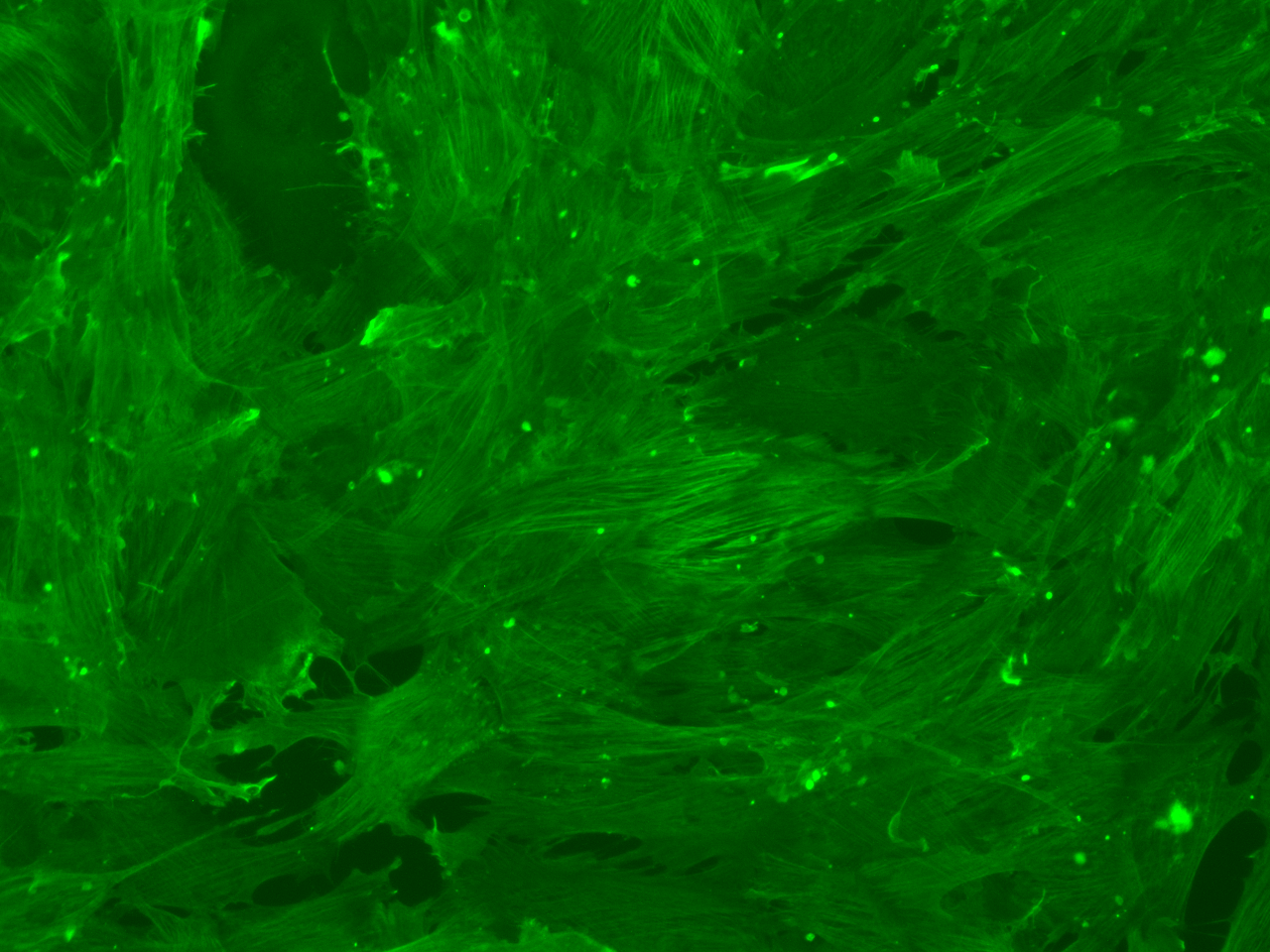

Supplement: Supplementary file 5 — Source data Fig. 3 [file 44319_2024_186_MOESM5_ESM.zip › EMBOR-2024-59433V3 Fig. 3 Source Data/3A/Ctrl aSMA IF.tif]

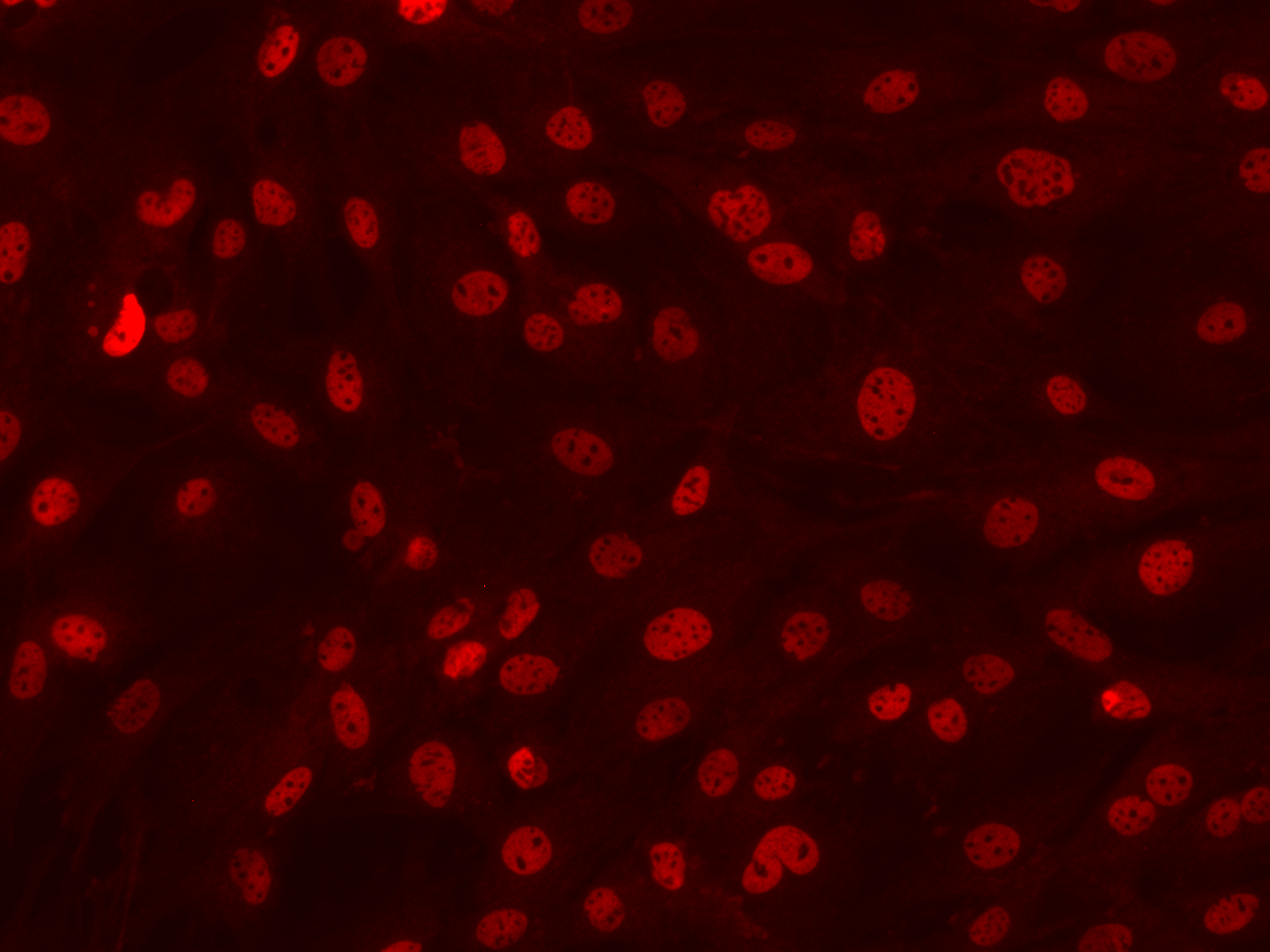

Supplement: Supplementary file 5 — Source data Fig. 3 [file 44319_2024_186_MOESM5_ESM.zip › EMBOR-2024-59433V3 Fig. 3 Source Data/3A/Ctrl Zeb1 IF.tif]

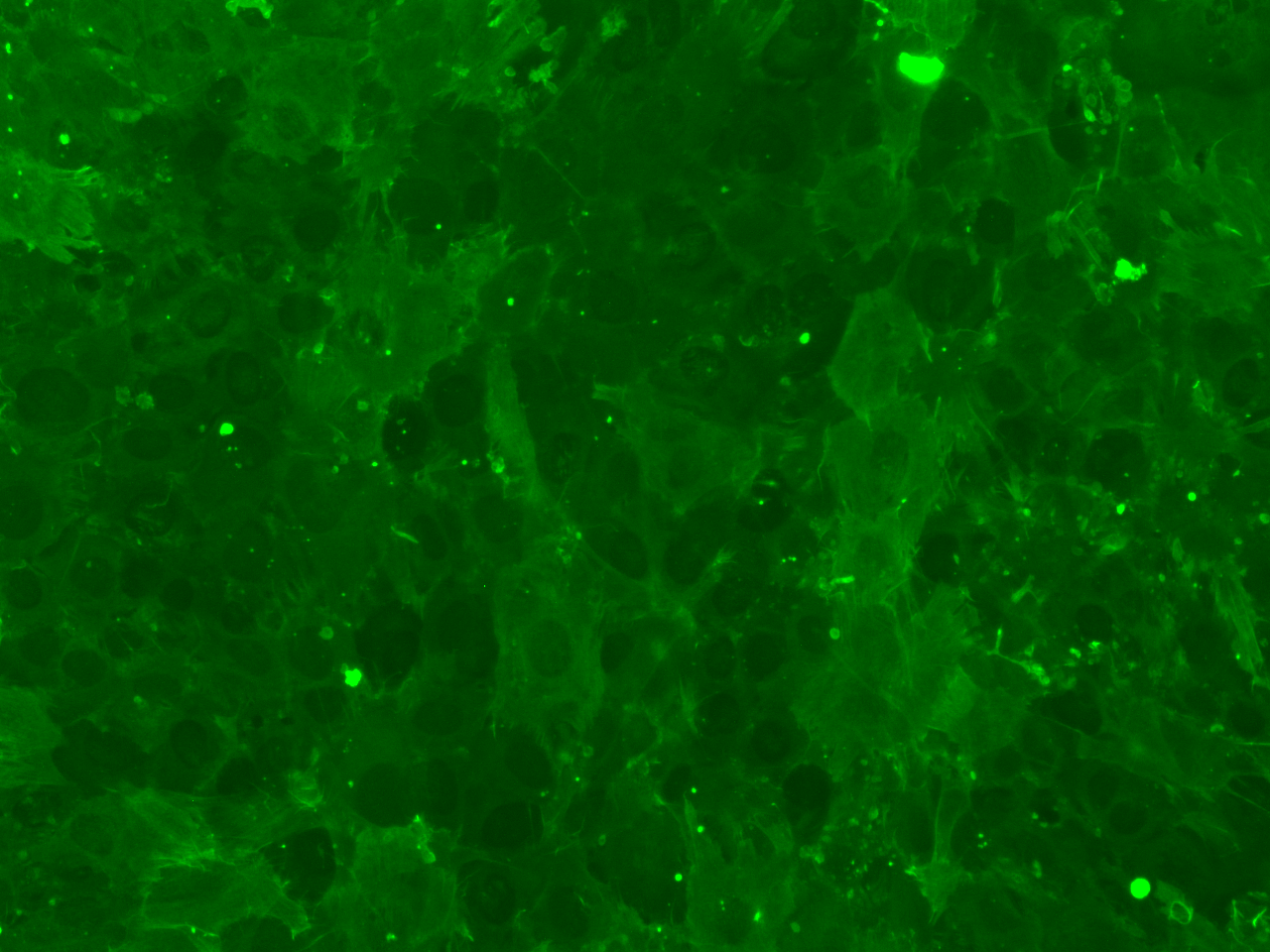

Supplement: Supplementary file 5 — Source data Fig. 3 [file 44319_2024_186_MOESM5_ESM.zip › EMBOR-2024-59433V3 Fig. 3 Source Data/3A/KO aSMA IF.tif]

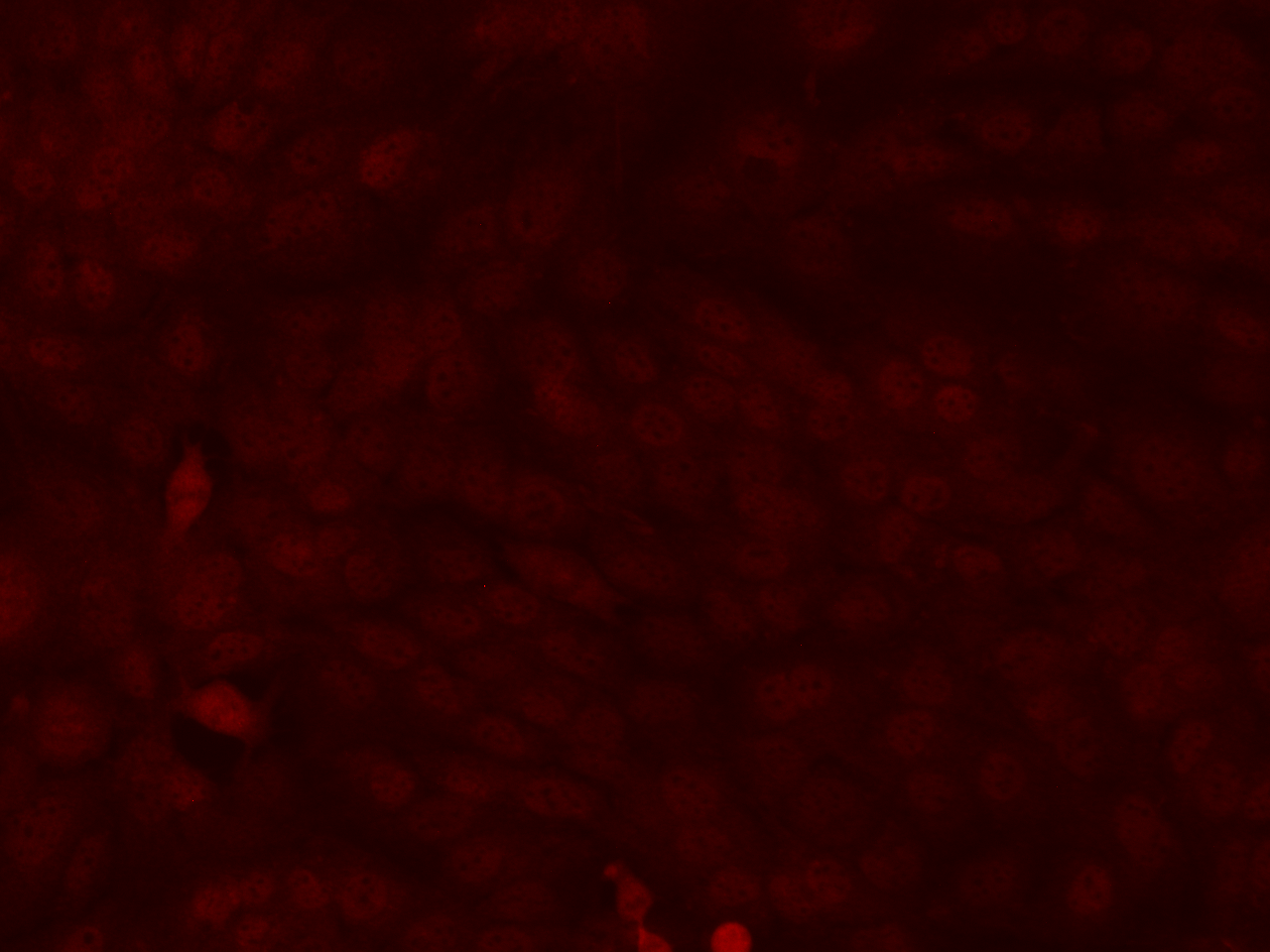

Supplement: Supplementary file 5 — Source data Fig. 3 [file 44319_2024_186_MOESM5_ESM.zip › EMBOR-2024-59433V3 Fig. 3 Source Data/3A/KO Zeb1 IF.tif]

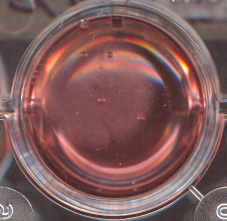

Supplement: Supplementary file 5 — Source data Fig. 3 [file 44319_2024_186_MOESM5_ESM.zip › EMBOR-2024-59433V3 Fig. 3 Source Data/3D/Ctrl contraction.png]

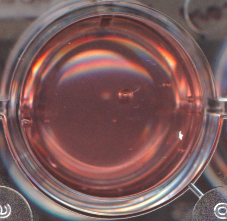

Supplement: Supplementary file 5 — Source data Fig. 3 [file 44319_2024_186_MOESM5_ESM.zip › EMBOR-2024-59433V3 Fig. 3 Source Data/3D/Del contraction.png]

## Slide 1
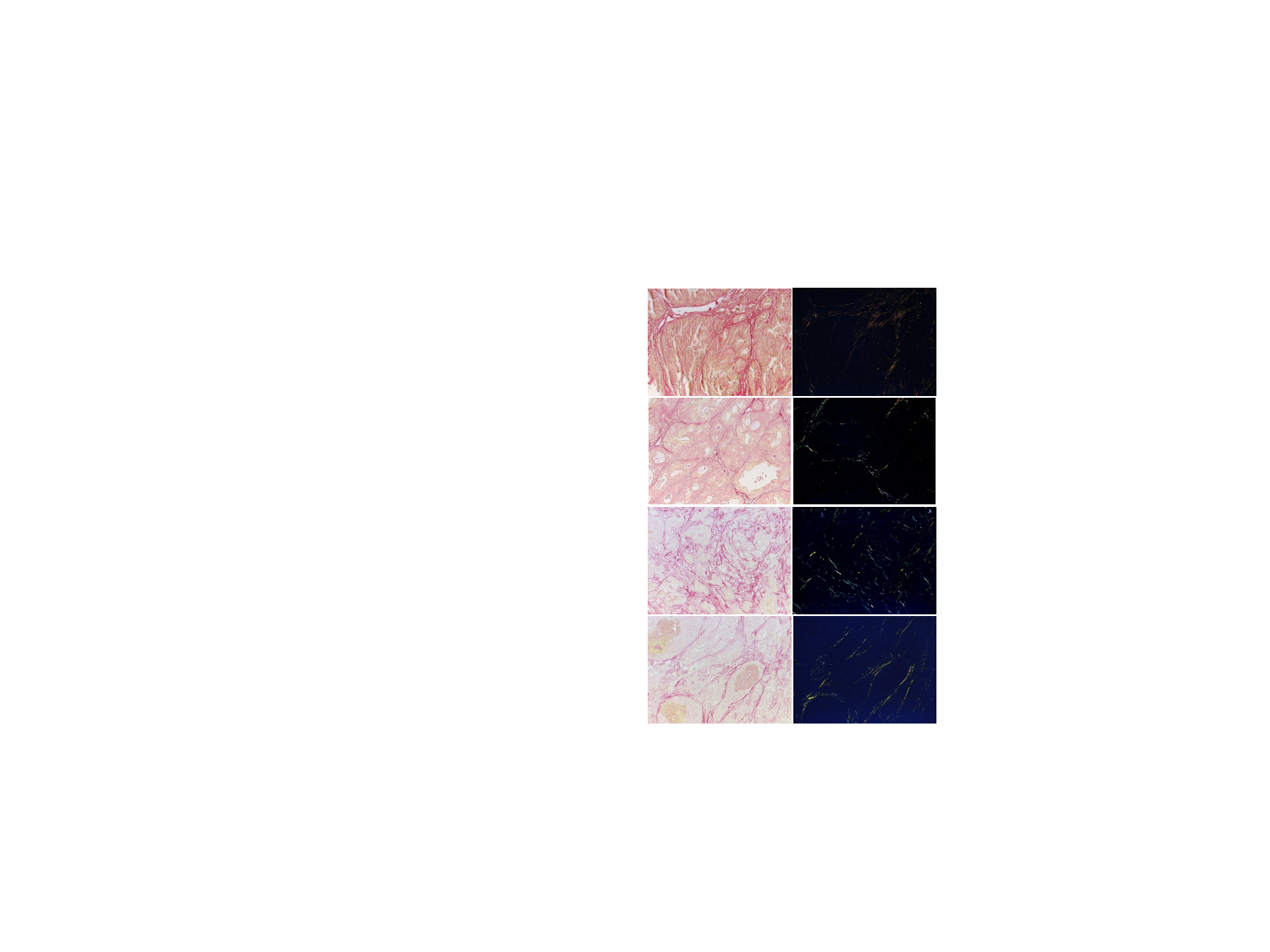

Supplement: Supplementary file 5 — Source data Fig. 3 [file 44319_2024_186_MOESM5_ESM.zip › EMBOR-2024-59433V3 Fig. 3 Source Data/3F/Fig. 3F image data.pptx]

## Slide 1
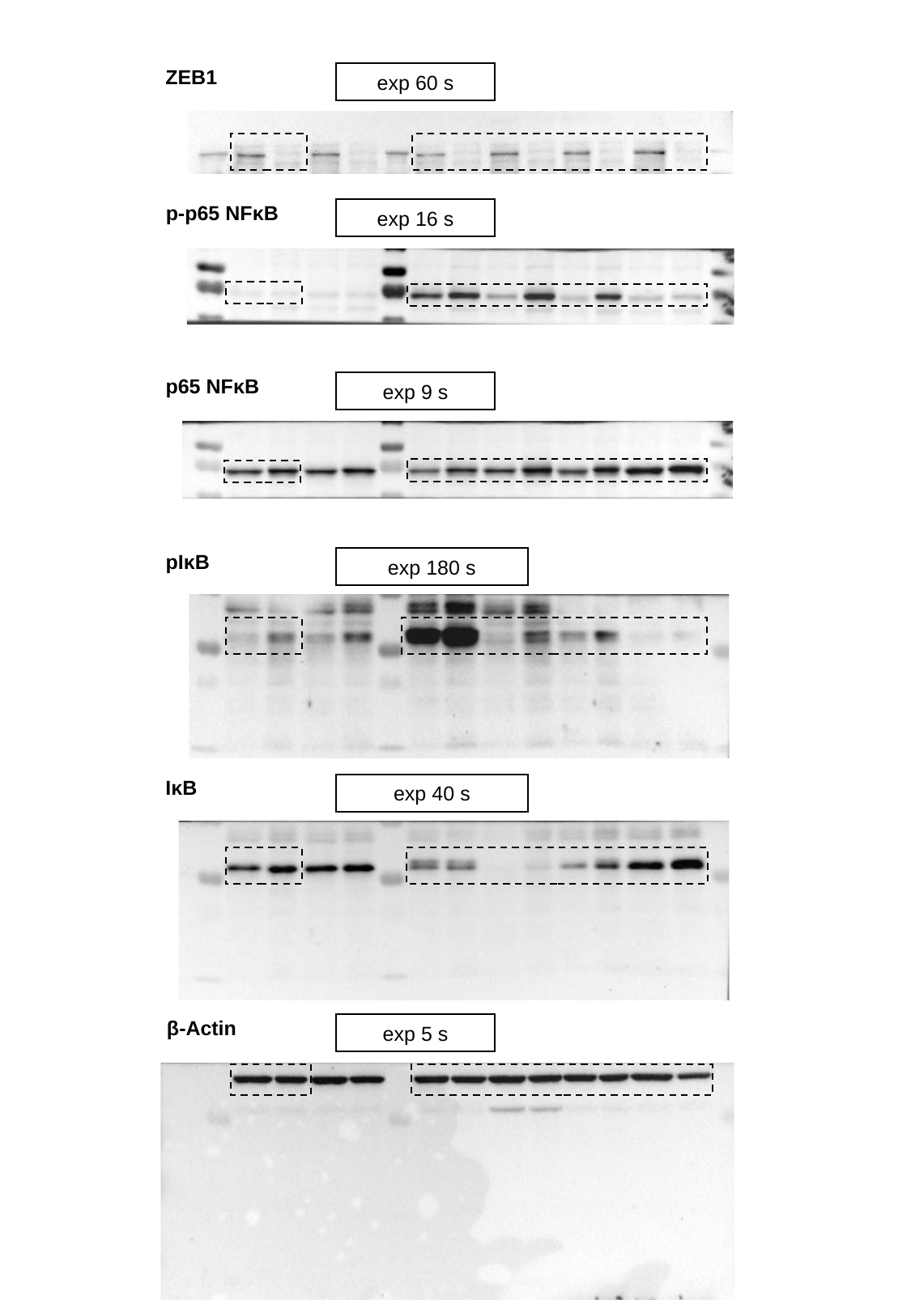

ZEB1
exp 60 s
p-p65 NFκB
exp 16 s
p65 NFκB
exp 9 s
pIκB
exp 180 s
IκB
exp 40 s
β-Actin
exp 5 s

Supplement: Supplementary file 6 — Source data Fig. 4 [file 44319_2024_186_MOESM6_ESM.zip › EMBOR-2024-59433V3 Fig. 4 Source Data/4D/Fig. 4D image data.pptx]

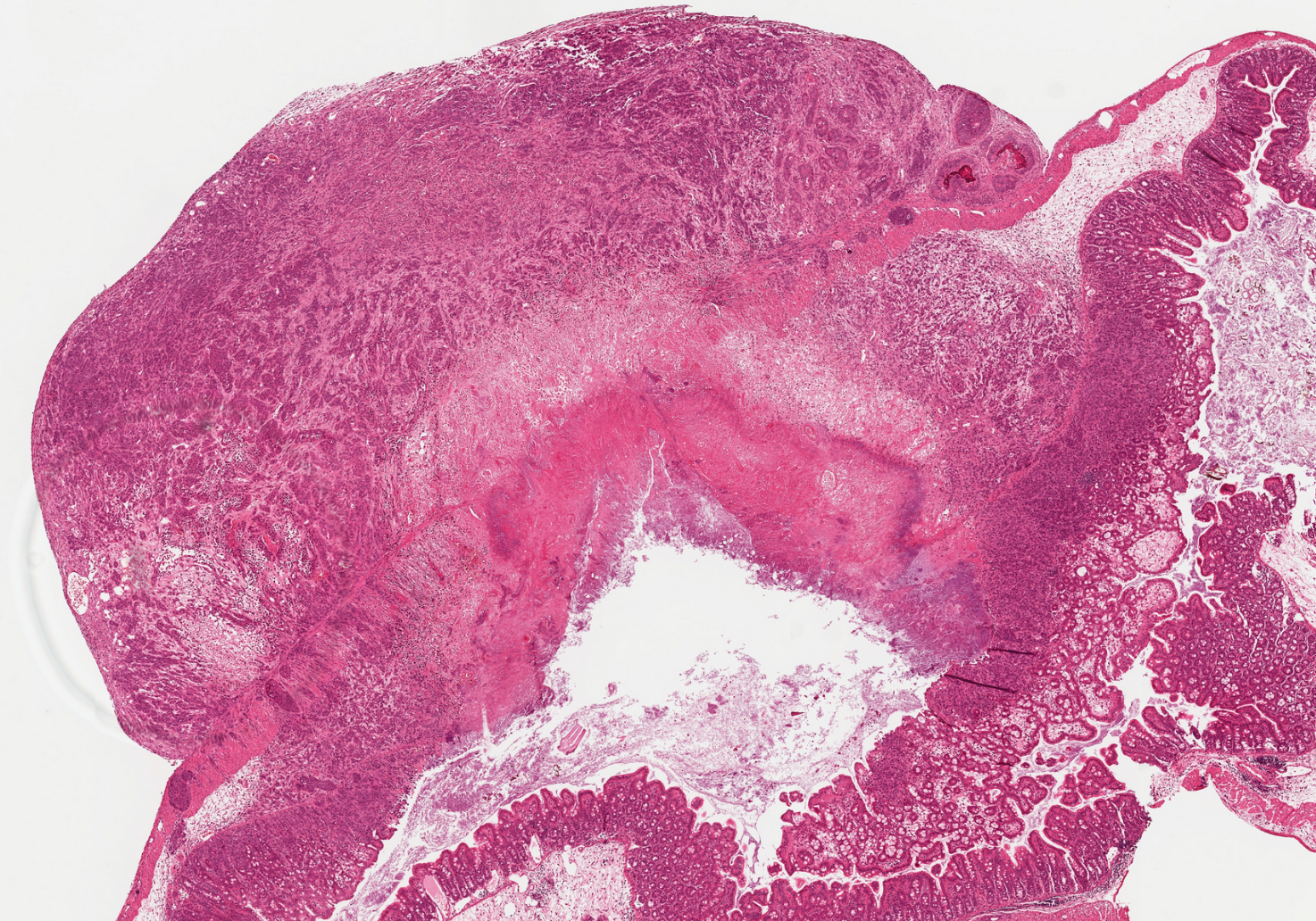

Supplement: Supplementary file 7 — Source data Fig. 5 [file 44319_2024_186_MOESM7_ESM.zip › EMBOR-2024-59433V3 Fig. 5 Source Data/5B/Ctrl Histology.png]

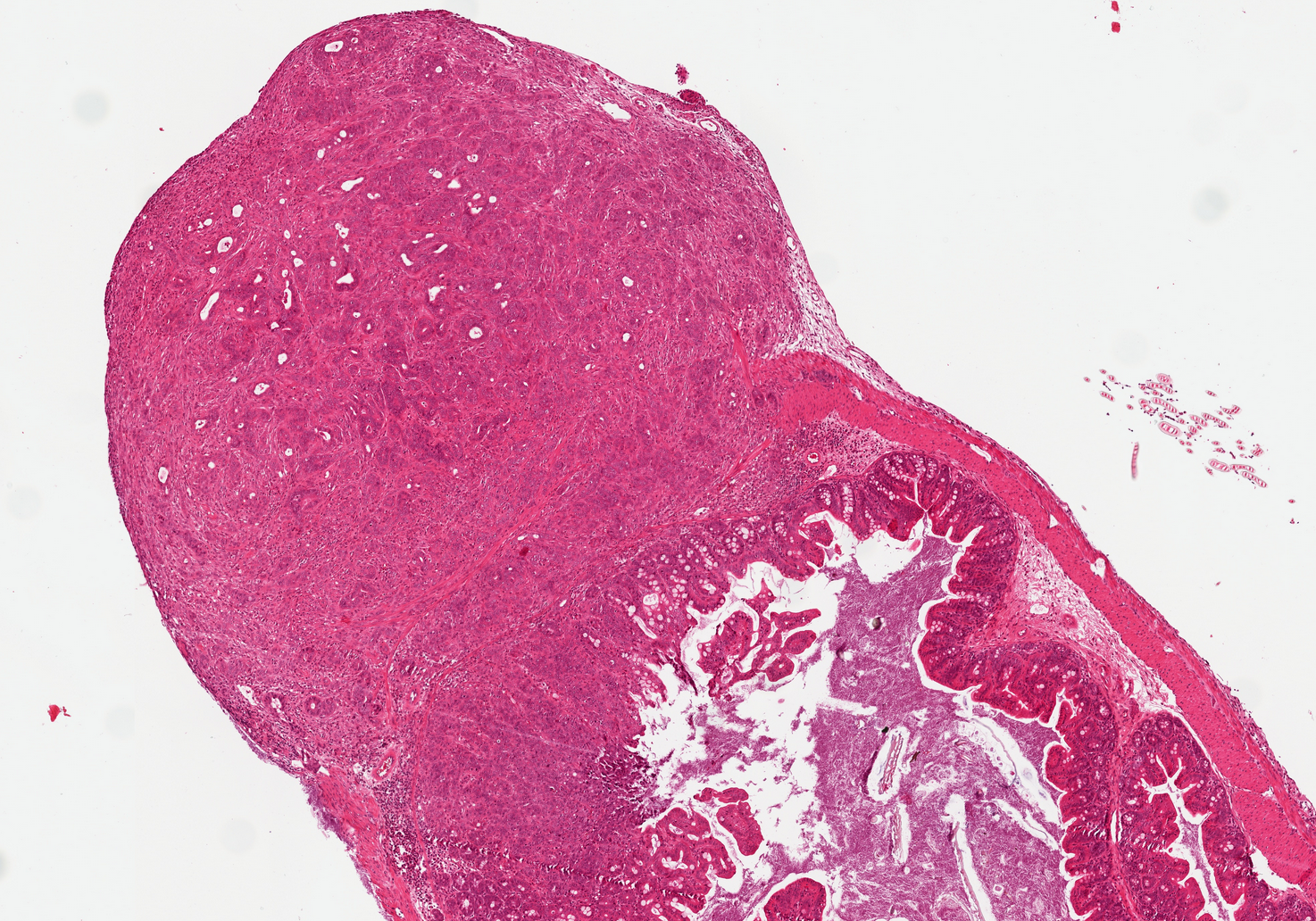

Supplement: Supplementary file 7 — Source data Fig. 5 [file 44319_2024_186_MOESM7_ESM.zip › EMBOR-2024-59433V3 Fig. 5 Source Data/5B/Del Histology.png]

## Slide 1
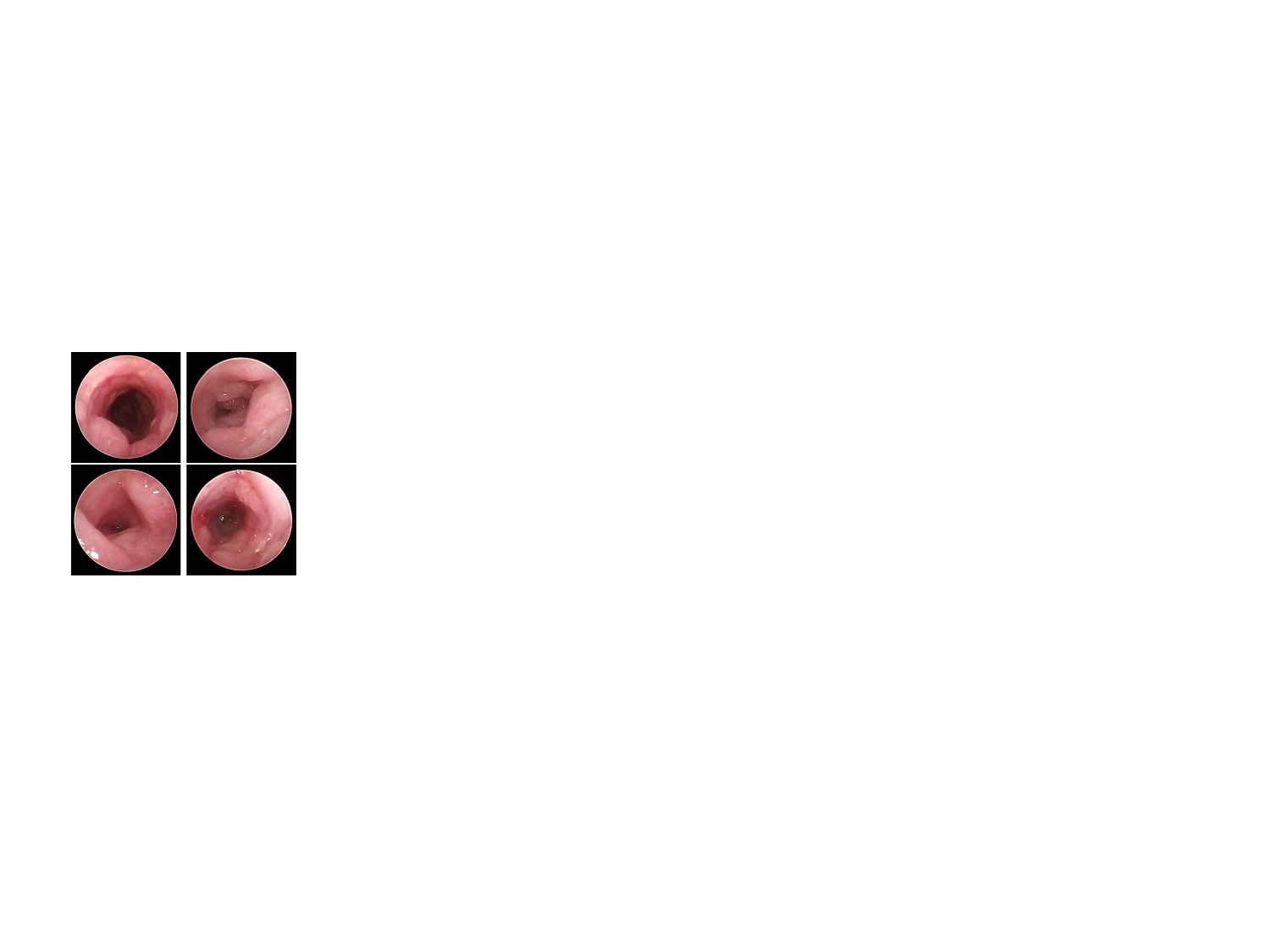

Supplement: Supplementary file 7 — Source data Fig. 5 [file 44319_2024_186_MOESM7_ESM.zip › EMBOR-2024-59433V3 Fig. 5 Source Data/5C/Fig. 5C image data.pptx]

## Slide 1
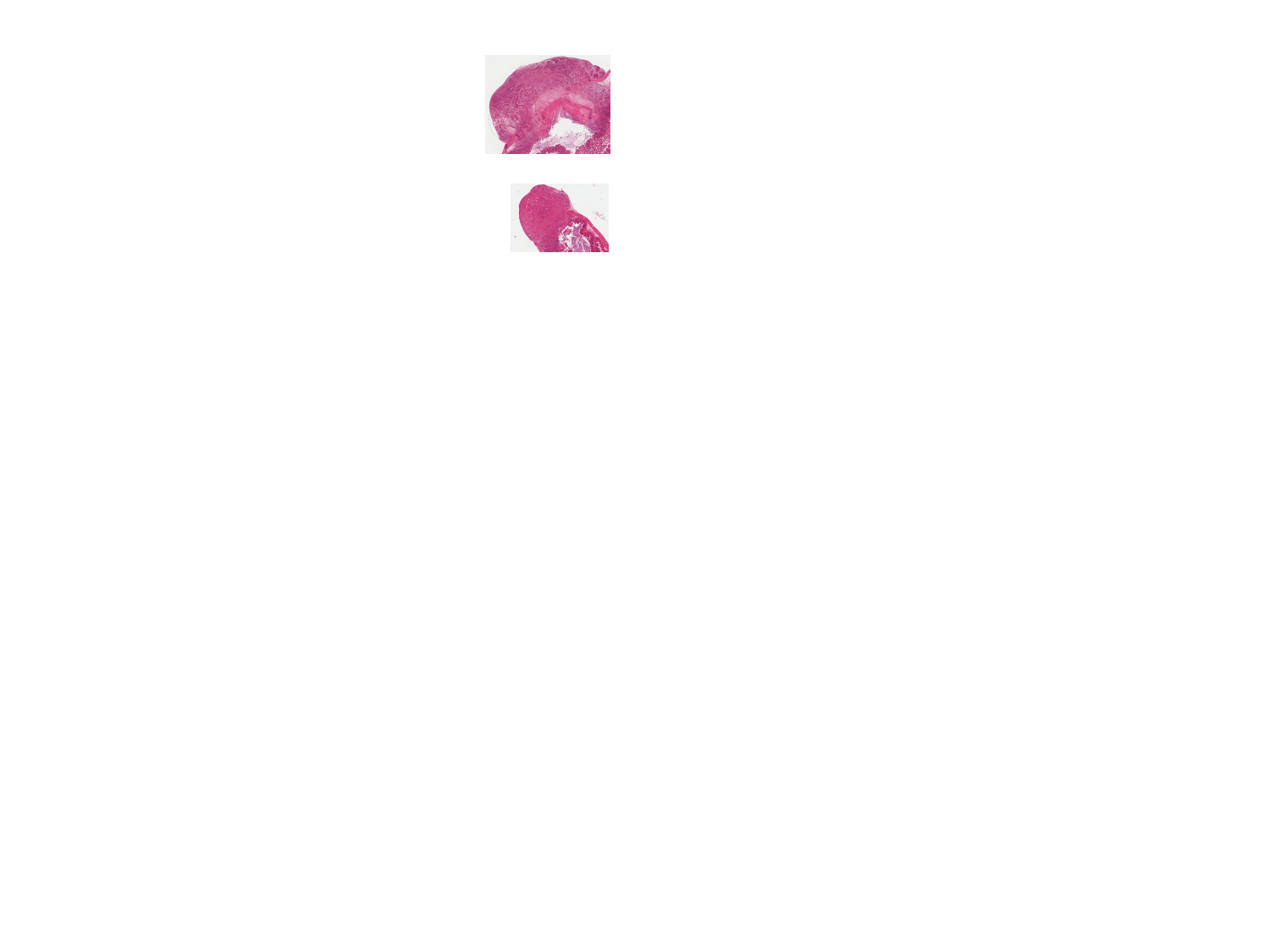

Supplement: Supplementary file 7 — Source data Fig. 5 [file 44319_2024_186_MOESM7_ESM.zip › EMBOR-2024-59433V3 Fig. 5 Source Data/Fig. 5B image data.pptx]

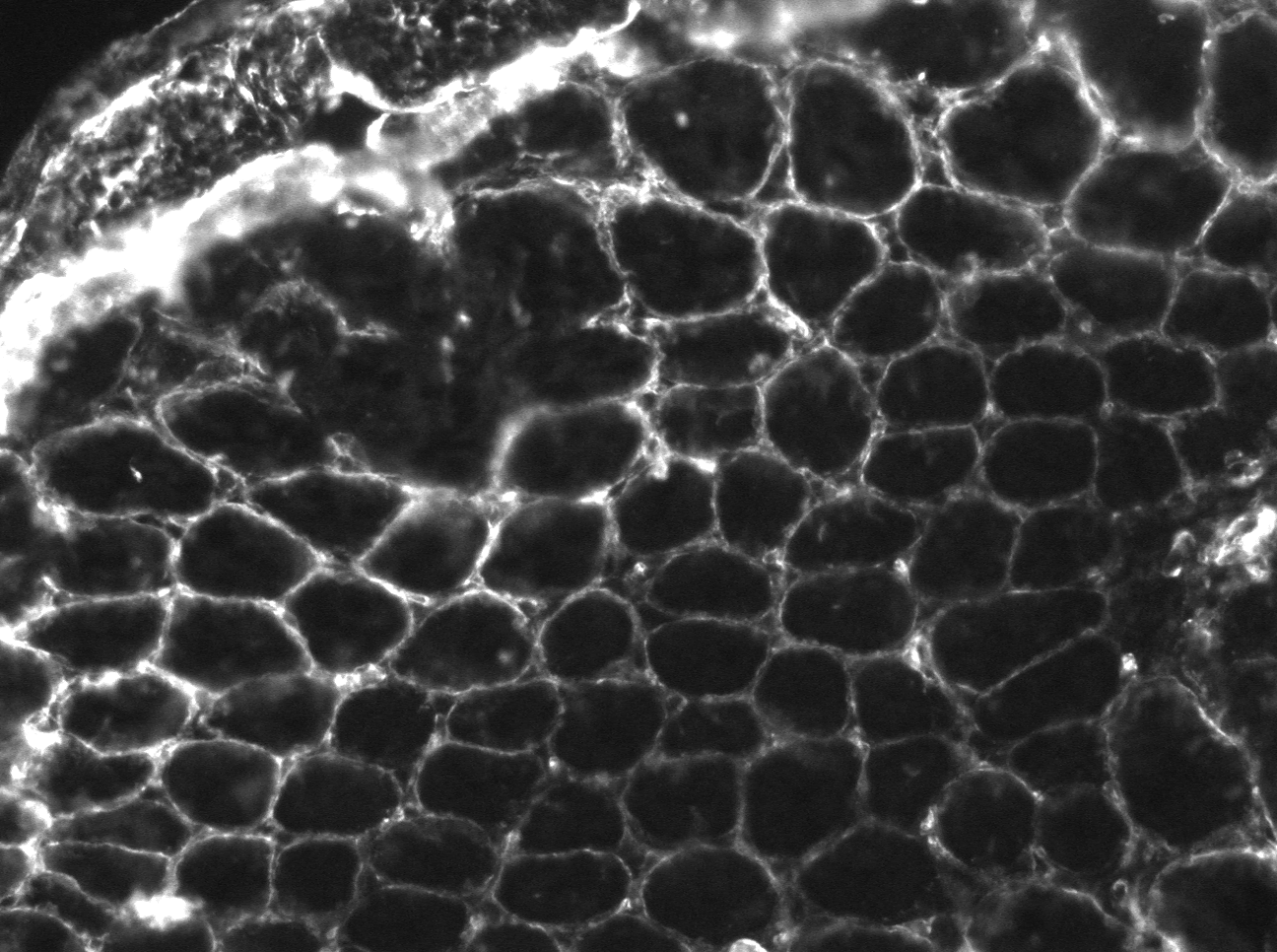

Supplement: Supplementary file 8 — Figure EV1-EV5, Appendix Fig. S1-S4 Source Data [file 44319_2024_186_MOESM8_ESM.zip › EMBOR-2024-59433V3 EV+Appendix Source Data/Appendix Fig S1/S1A/COL6.tif]

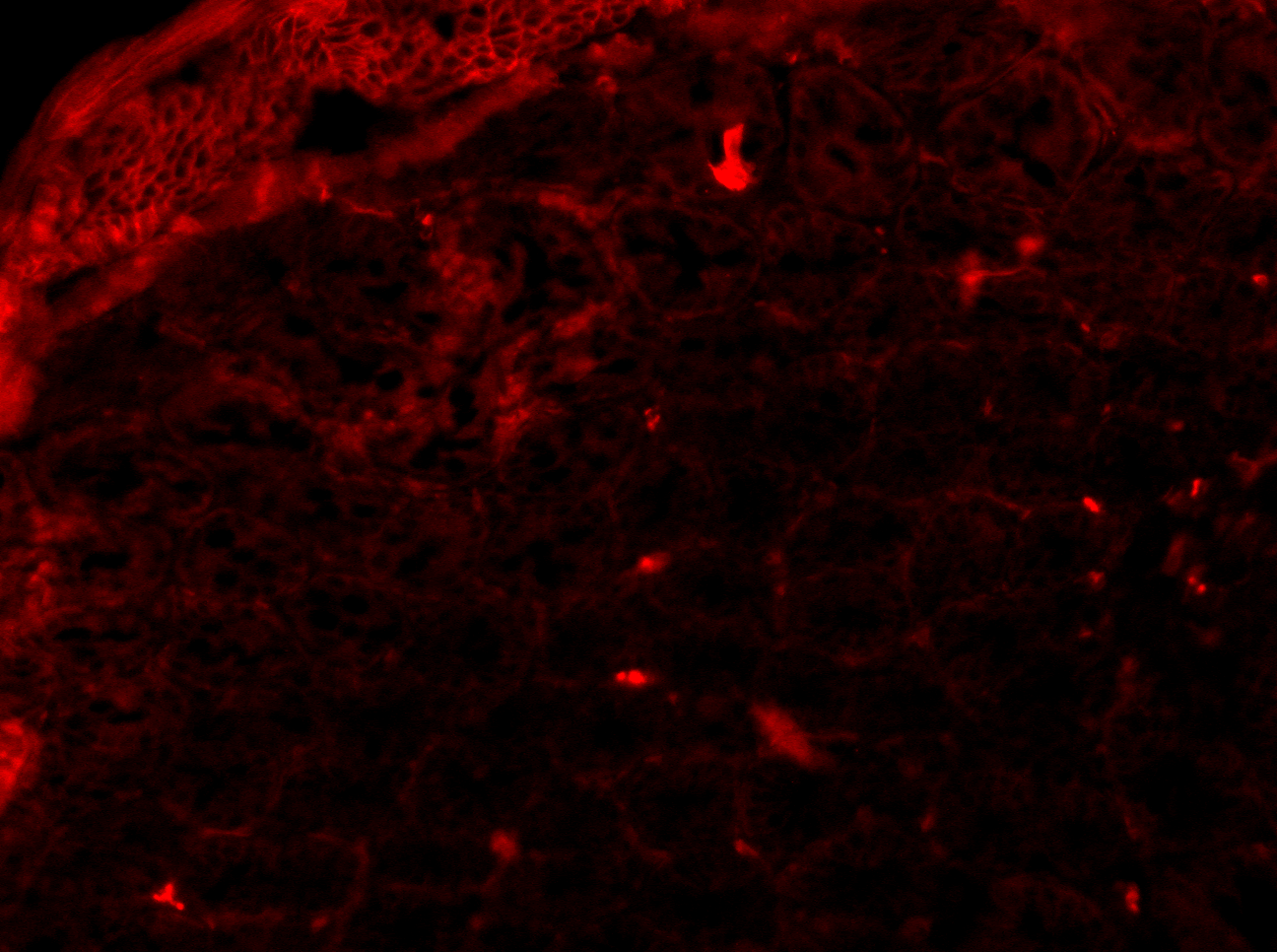

Supplement: Supplementary file 8 — Figure EV1-EV5, Appendix Fig. S1-S4 Source Data [file 44319_2024_186_MOESM8_ESM.zip › EMBOR-2024-59433V3 EV+Appendix Source Data/Appendix Fig S1/S1A/GFP.tif]

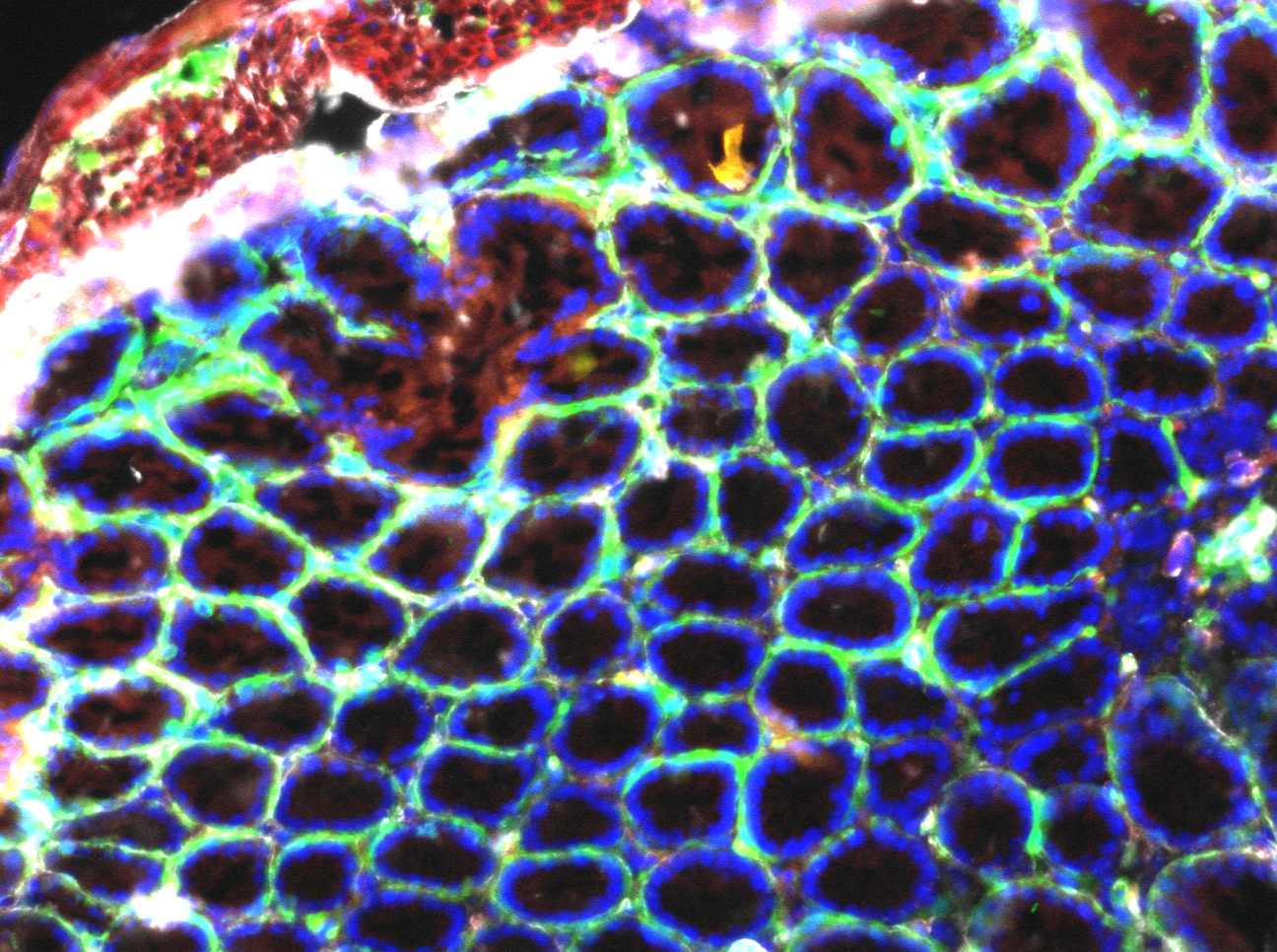

Supplement: Supplementary file 8 — Figure EV1-EV5, Appendix Fig. S1-S4 Source Data [file 44319_2024_186_MOESM8_ESM.zip › EMBOR-2024-59433V3 EV+Appendix Source Data/Appendix Fig S1/S1A/merge.tif]

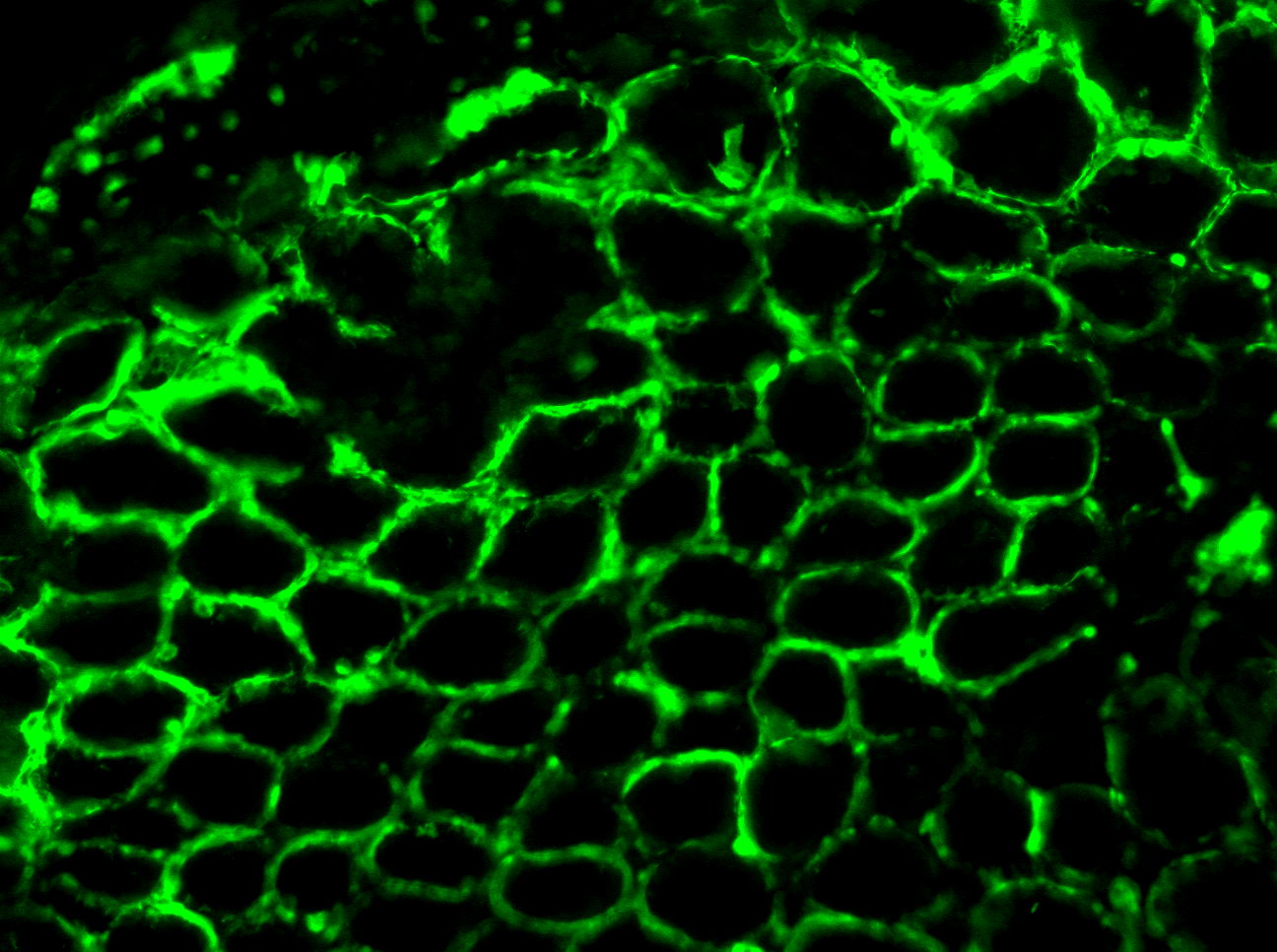

Supplement: Supplementary file 8 — Figure EV1-EV5, Appendix Fig. S1-S4 Source Data [file 44319_2024_186_MOESM8_ESM.zip › EMBOR-2024-59433V3 EV+Appendix Source Data/Appendix Fig S1/S1A/tdTomato.tif]

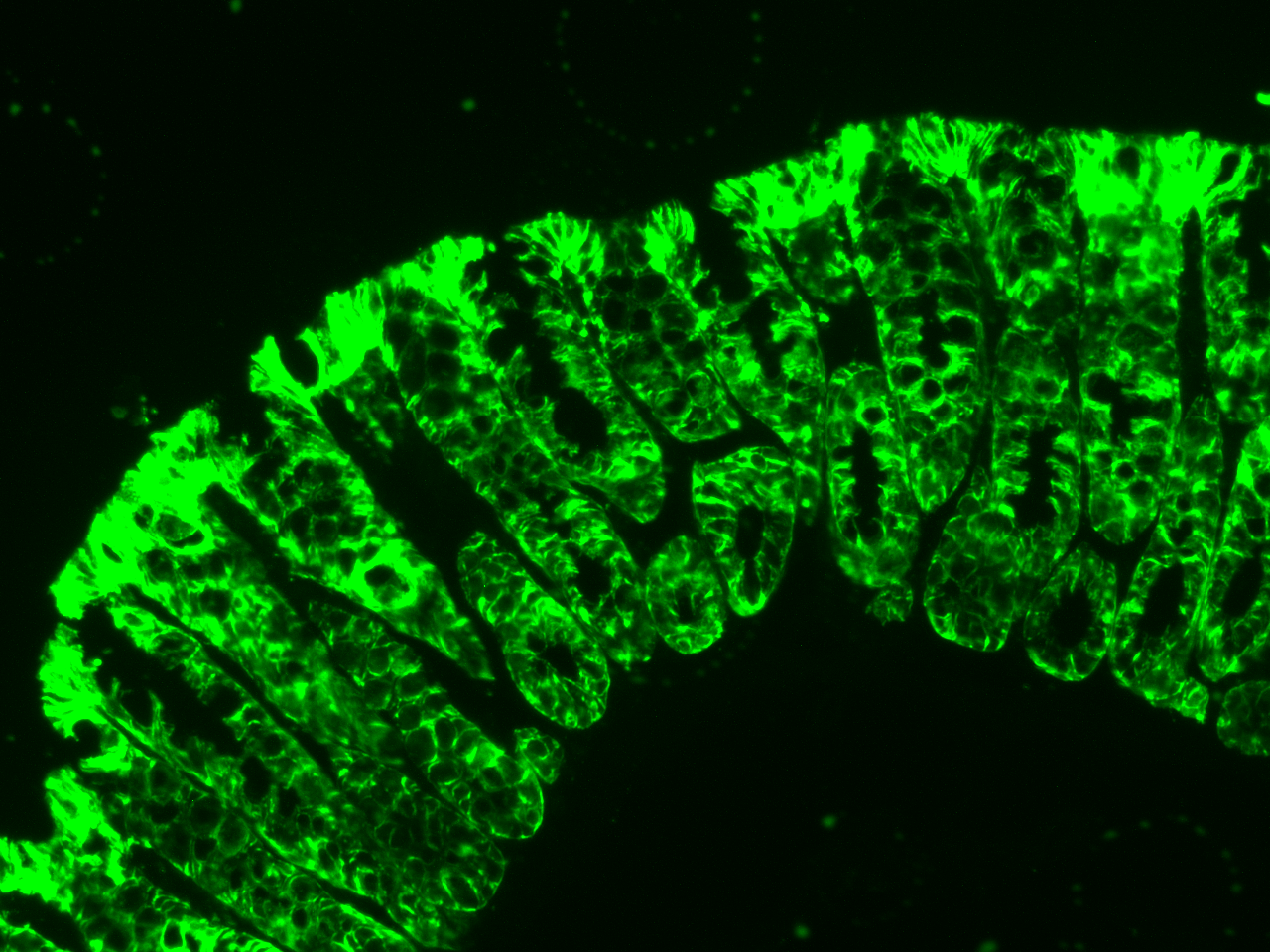

Supplement: Supplementary file 8 — Figure EV1-EV5, Appendix Fig. S1-S4 Source Data [file 44319_2024_186_MOESM8_ESM.zip › EMBOR-2024-59433V3 EV+Appendix Source Data/Appendix Fig S1/S1B/Col1a2 IF ECAD.tif]

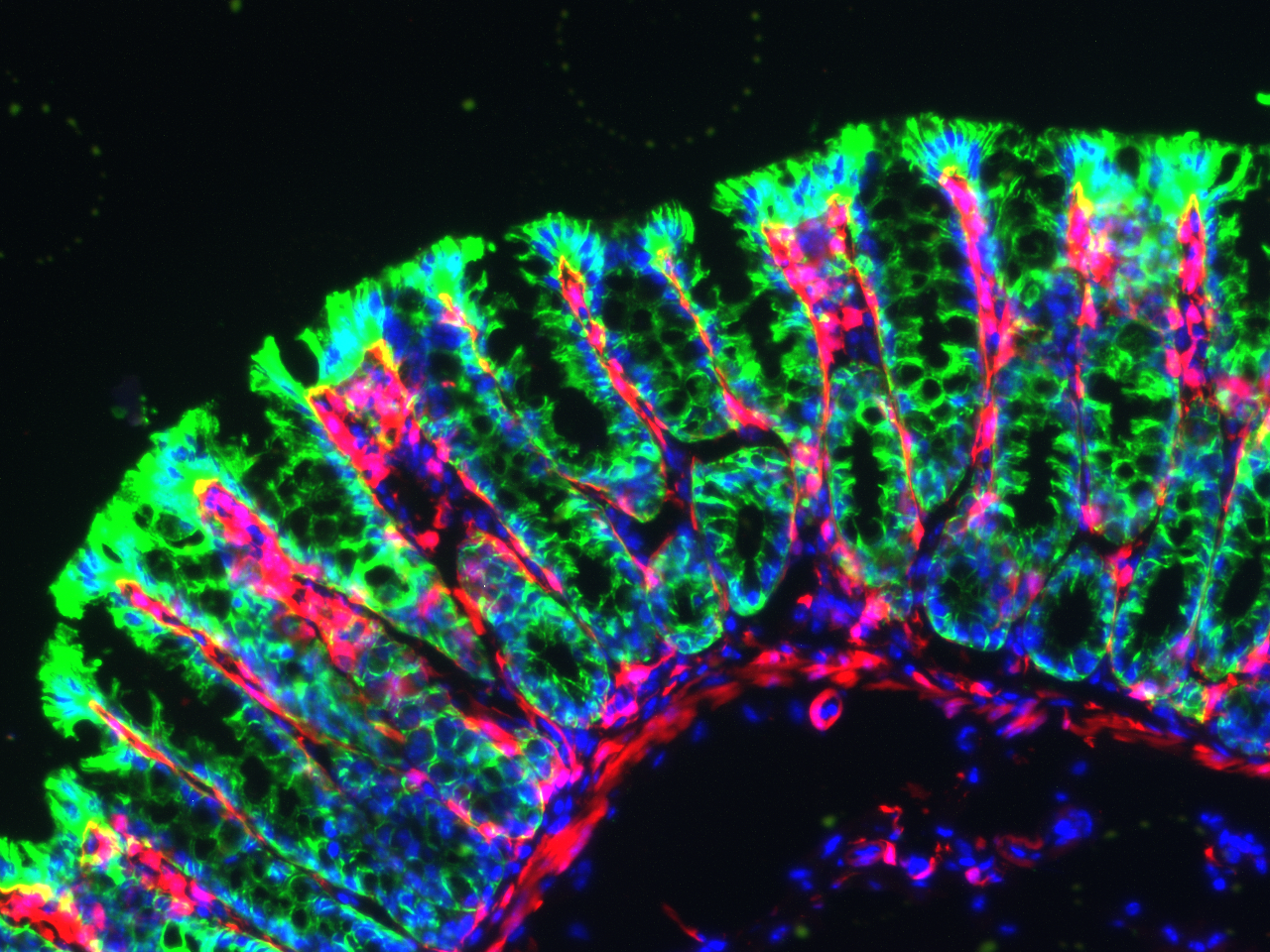

Supplement: Supplementary file 8 — Figure EV1-EV5, Appendix Fig. S1-S4 Source Data [file 44319_2024_186_MOESM8_ESM.zip › EMBOR-2024-59433V3 EV+Appendix Source Data/Appendix Fig S1/S1B/Col1a2 IF Merge.tif]

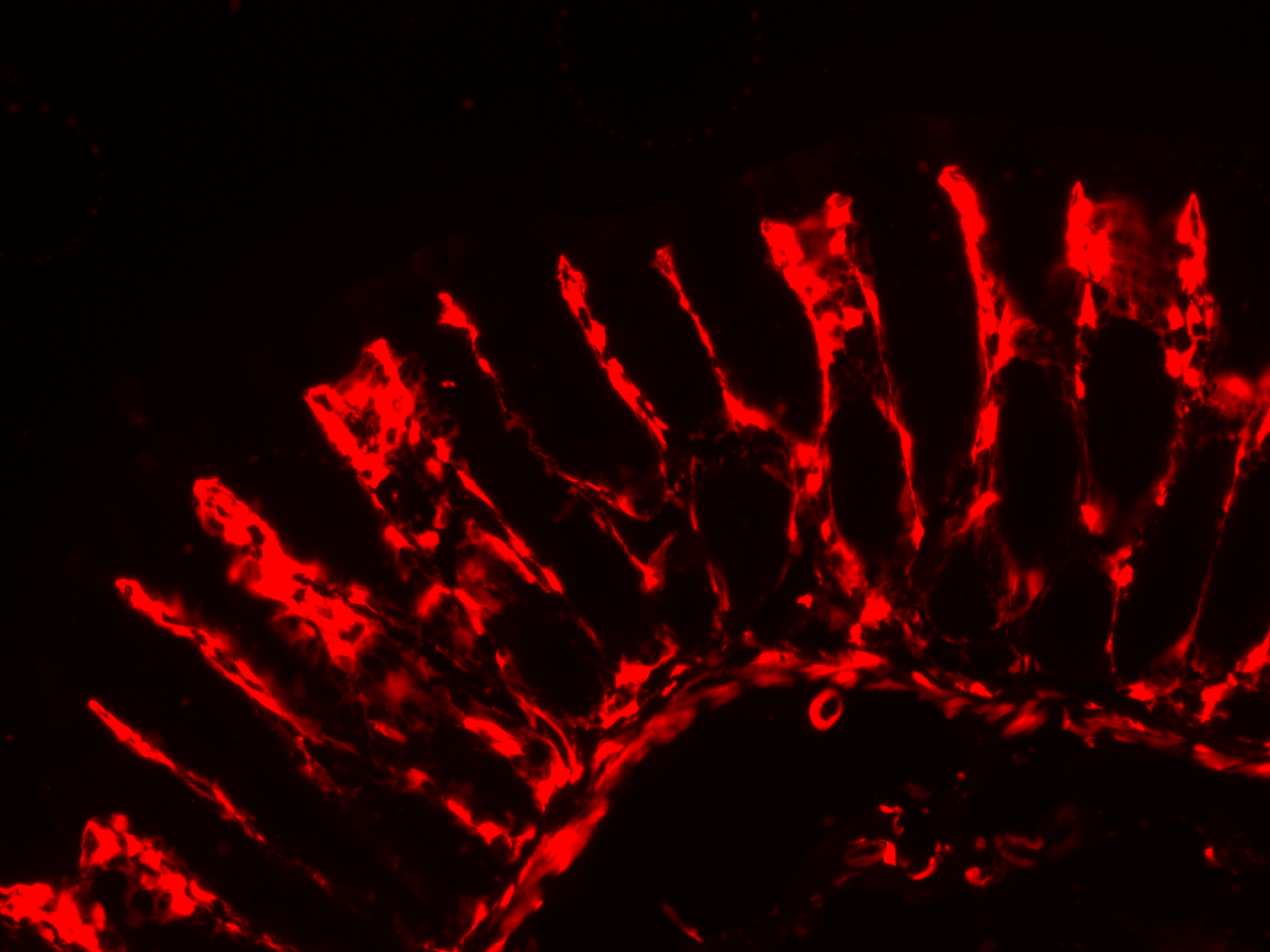

Supplement: Supplementary file 8 — Figure EV1-EV5, Appendix Fig. S1-S4 Source Data [file 44319_2024_186_MOESM8_ESM.zip › EMBOR-2024-59433V3 EV+Appendix Source Data/Appendix Fig S1/S1B/Col1a2 IF RFP.tif]

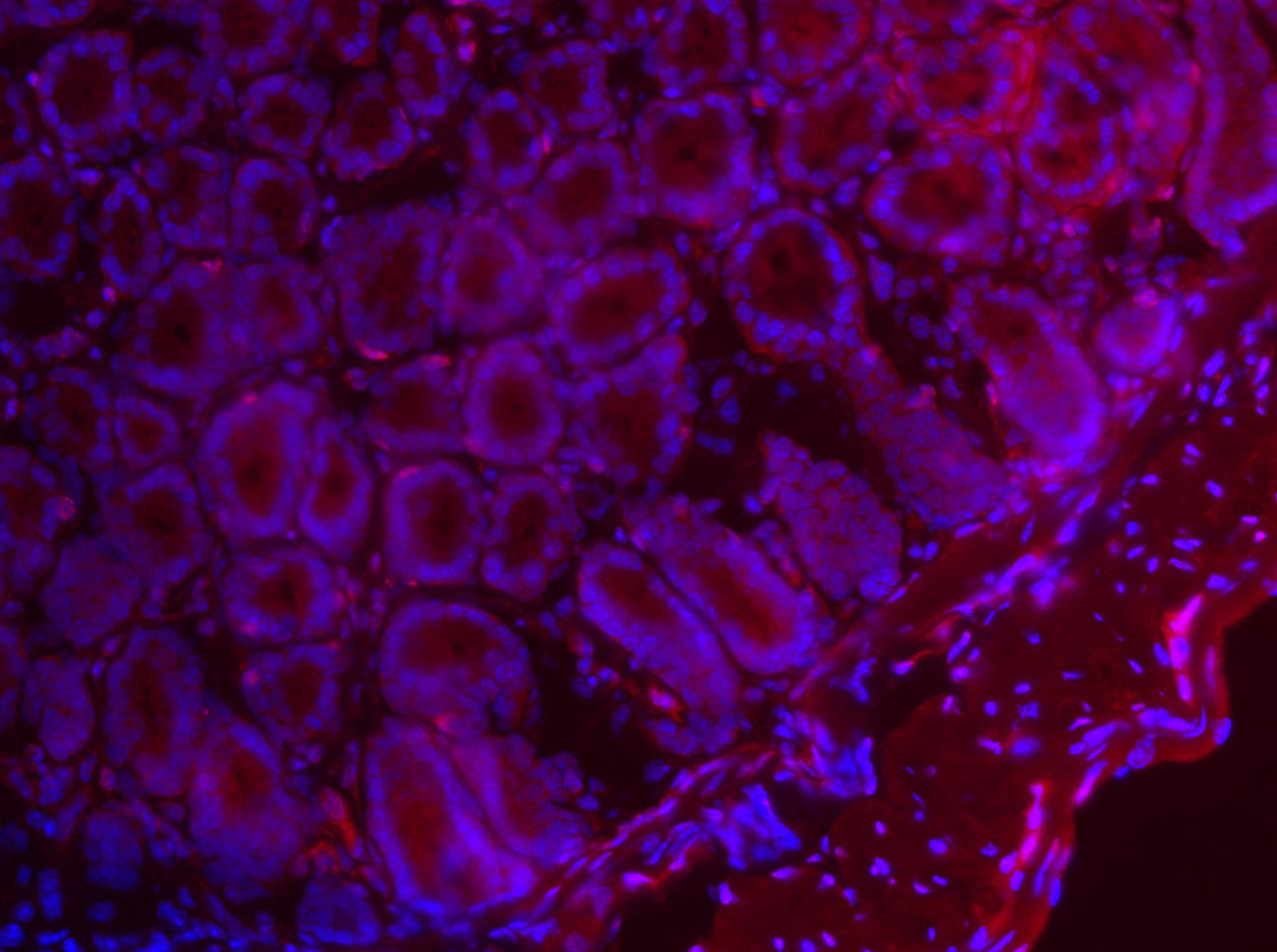

Supplement: Supplementary file 8 — Figure EV1-EV5, Appendix Fig. S1-S4 Source Data [file 44319_2024_186_MOESM8_ESM.zip › EMBOR-2024-59433V3 EV+Appendix Source Data/Appendix Fig S1/S1D/Ctrl DAPI ZEB1 IF.tif]

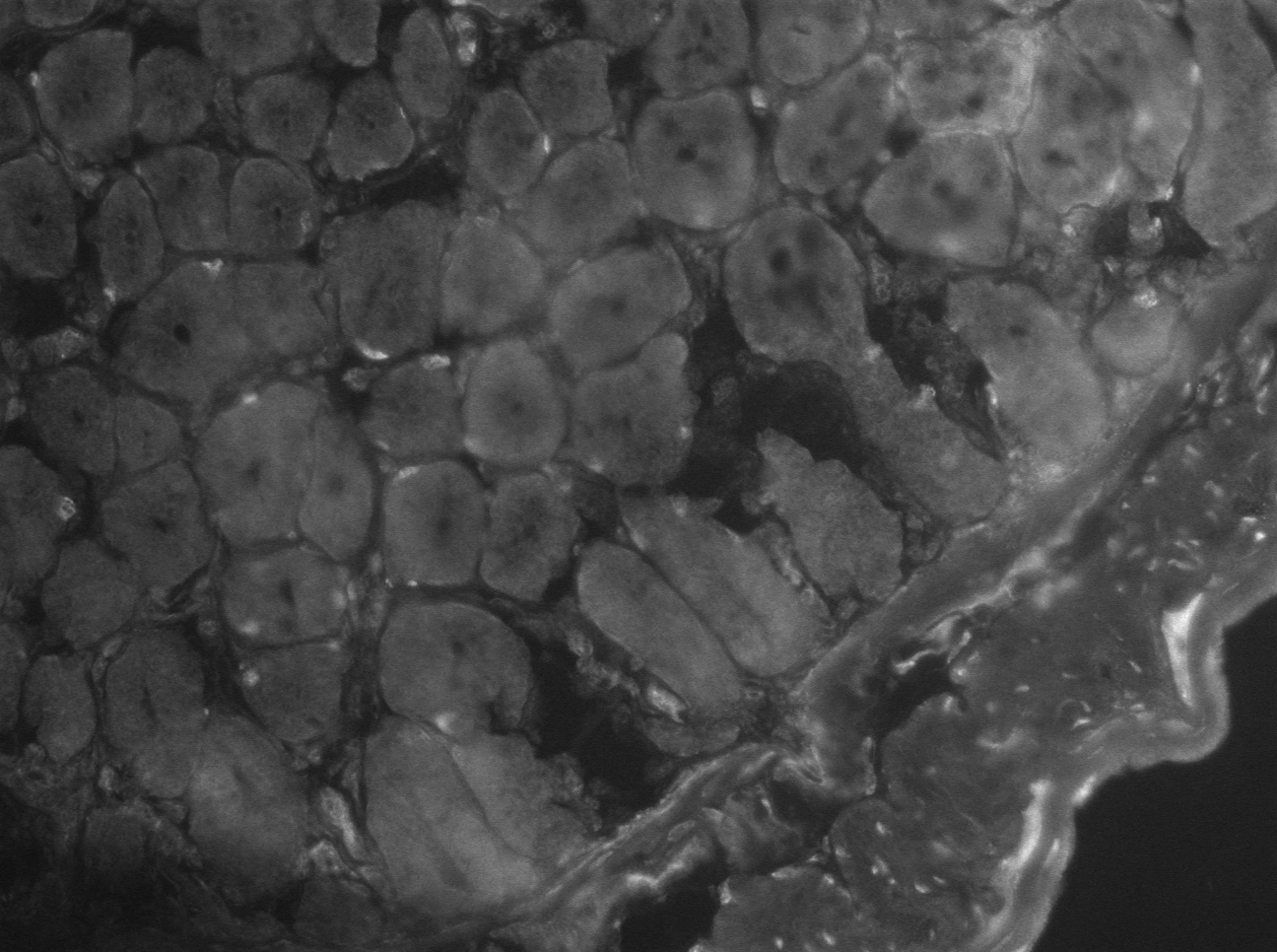

Supplement: Supplementary file 8 — Figure EV1-EV5, Appendix Fig. S1-S4 Source Data [file 44319_2024_186_MOESM8_ESM.zip › EMBOR-2024-59433V3 EV+Appendix Source Data/Appendix Fig S1/S1D/Ctrl ZEB1.tif]

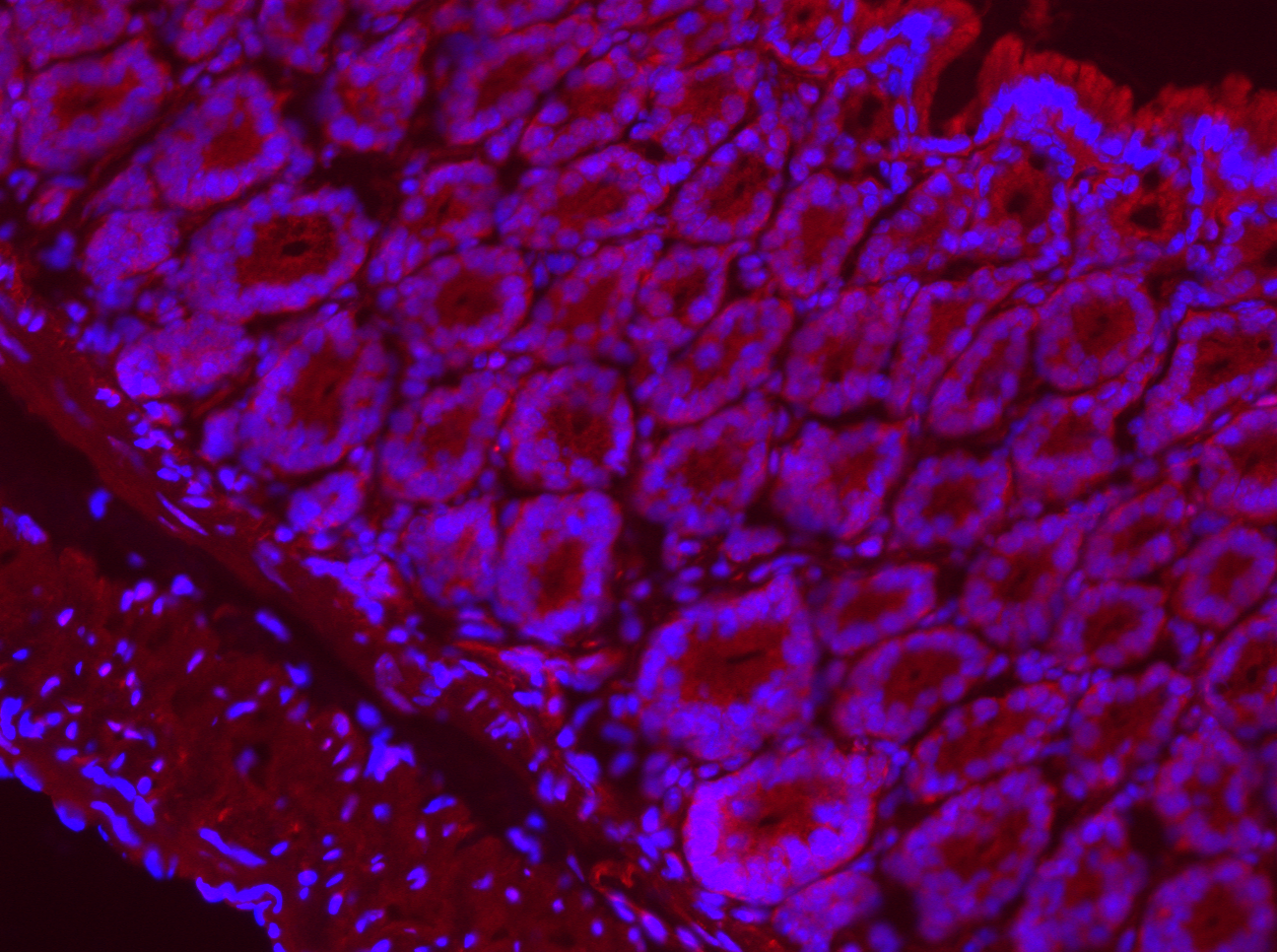

Supplement: Supplementary file 8 — Figure EV1-EV5, Appendix Fig. S1-S4 Source Data [file 44319_2024_186_MOESM8_ESM.zip › EMBOR-2024-59433V3 EV+Appendix Source Data/Appendix Fig S1/S1D/Del DAPI ZEB1 IF.tif]

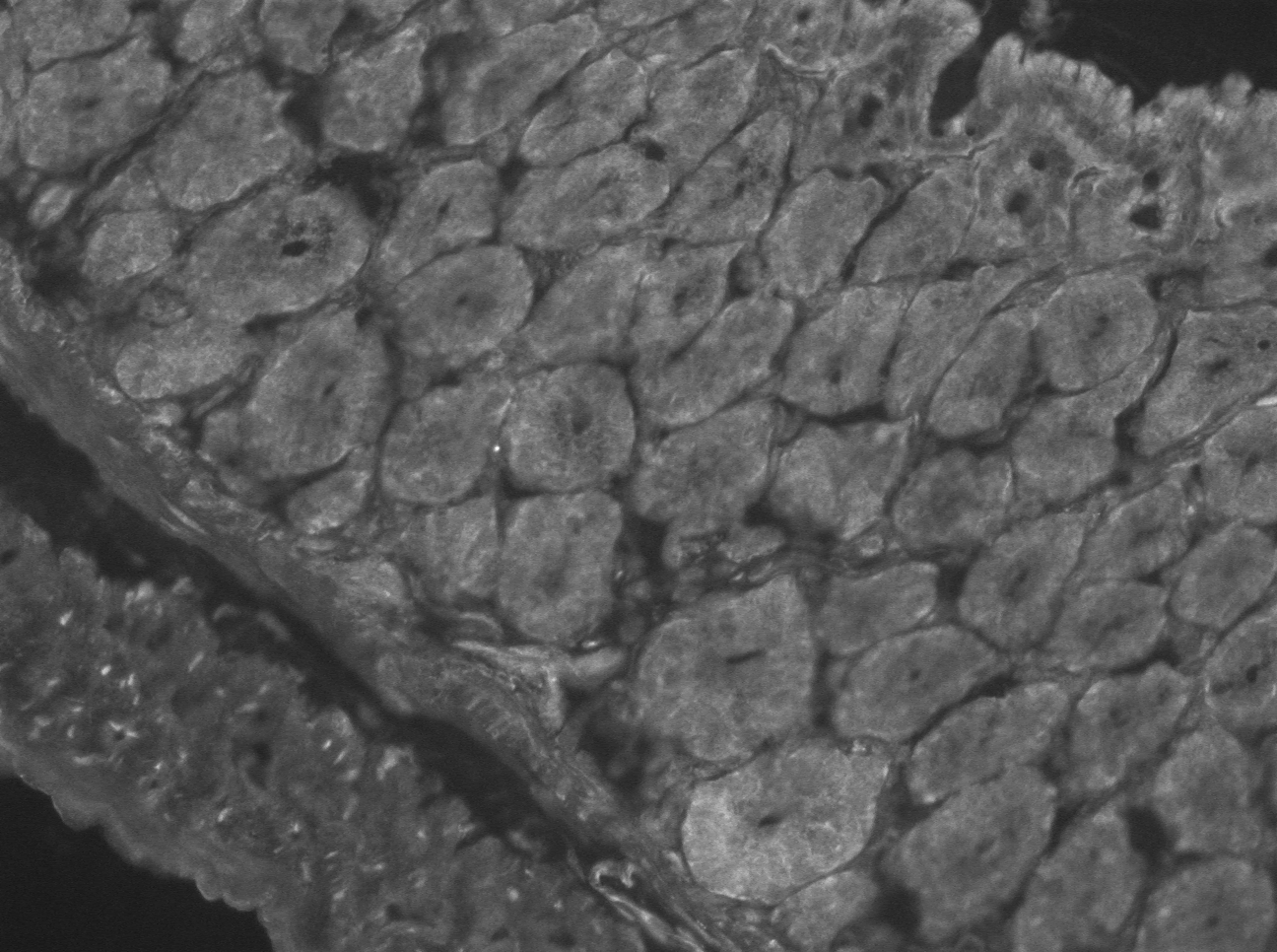

Supplement: Supplementary file 8 — Figure EV1-EV5, Appendix Fig. S1-S4 Source Data [file 44319_2024_186_MOESM8_ESM.zip › EMBOR-2024-59433V3 EV+Appendix Source Data/Appendix Fig S1/S1D/Del ZEB1 IF.tif]

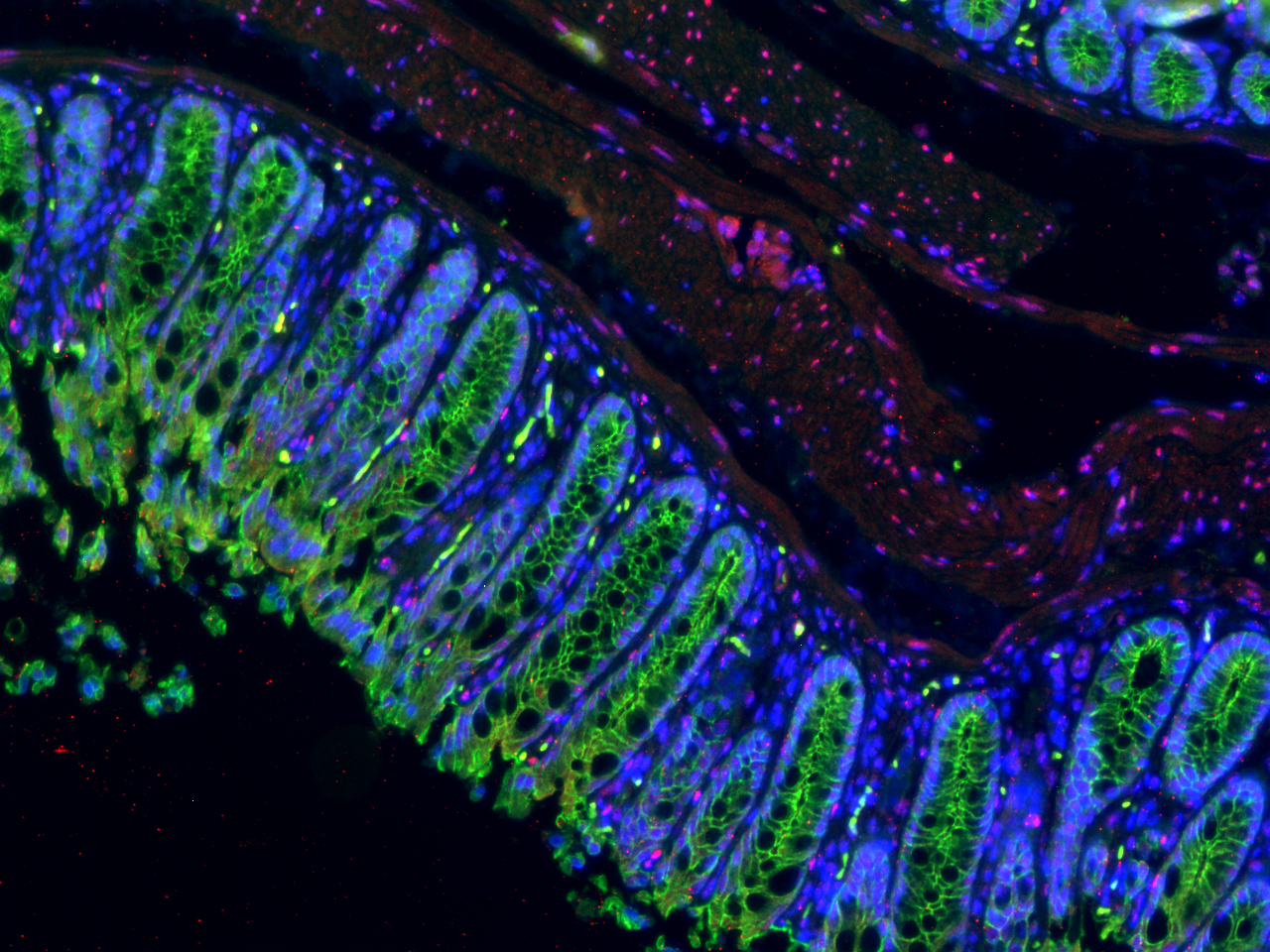

Supplement: Supplementary file 8 — Figure EV1-EV5, Appendix Fig. S1-S4 Source Data [file 44319_2024_186_MOESM8_ESM.zip › EMBOR-2024-59433V3 EV+Appendix Source Data/Appendix Fig S1/S1E/Ctrl IF Overview.tif]

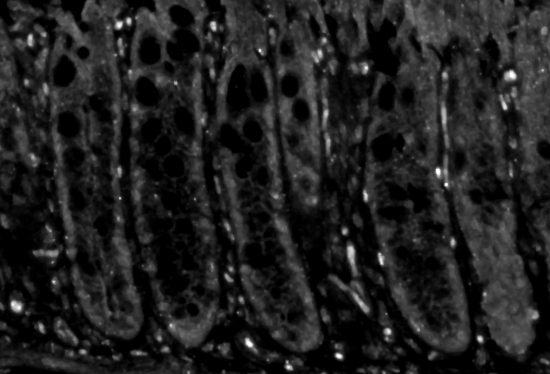

Supplement: Supplementary file 8 — Figure EV1-EV5, Appendix Fig. S1-S4 Source Data [file 44319_2024_186_MOESM8_ESM.zip › EMBOR-2024-59433V3 EV+Appendix Source Data/Appendix Fig S1/S1E/Ctrl IF Zoom.tif]

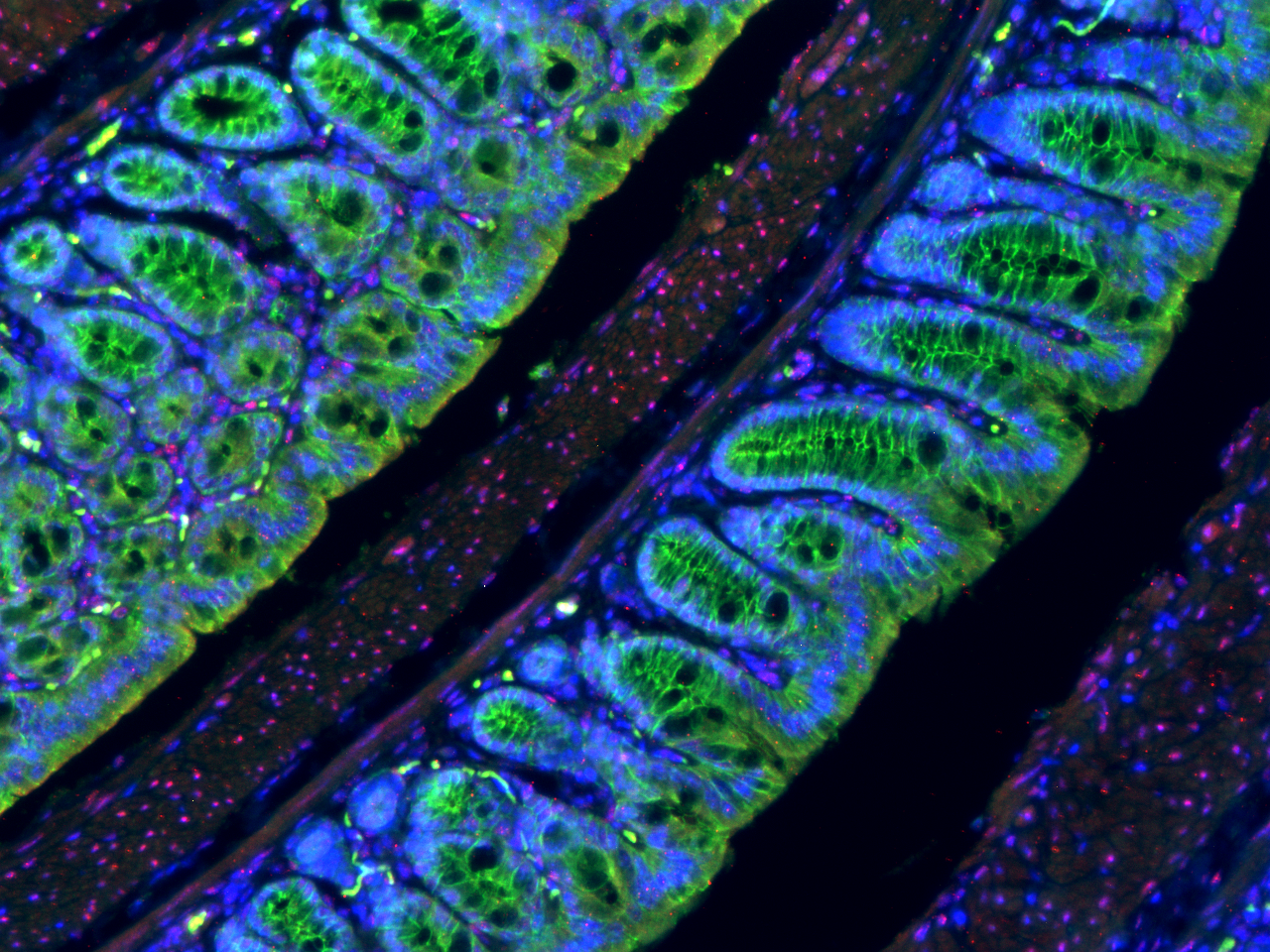

Supplement: Supplementary file 8 — Figure EV1-EV5, Appendix Fig. S1-S4 Source Data [file 44319_2024_186_MOESM8_ESM.zip › EMBOR-2024-59433V3 EV+Appendix Source Data/Appendix Fig S1/S1E/Del IF Overview.tif]

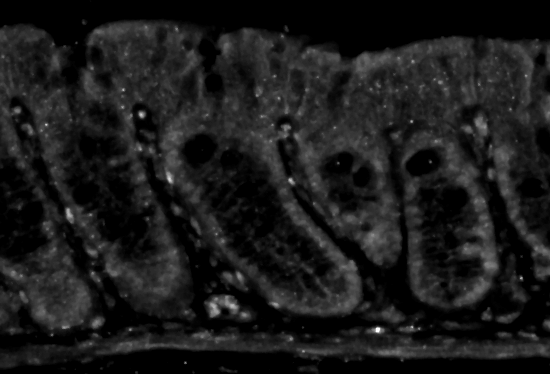

Supplement: Supplementary file 8 — Figure EV1-EV5, Appendix Fig. S1-S4 Source Data [file 44319_2024_186_MOESM8_ESM.zip › EMBOR-2024-59433V3 EV+Appendix Source Data/Appendix Fig S1/S1E/Del IF Zoom.tif]

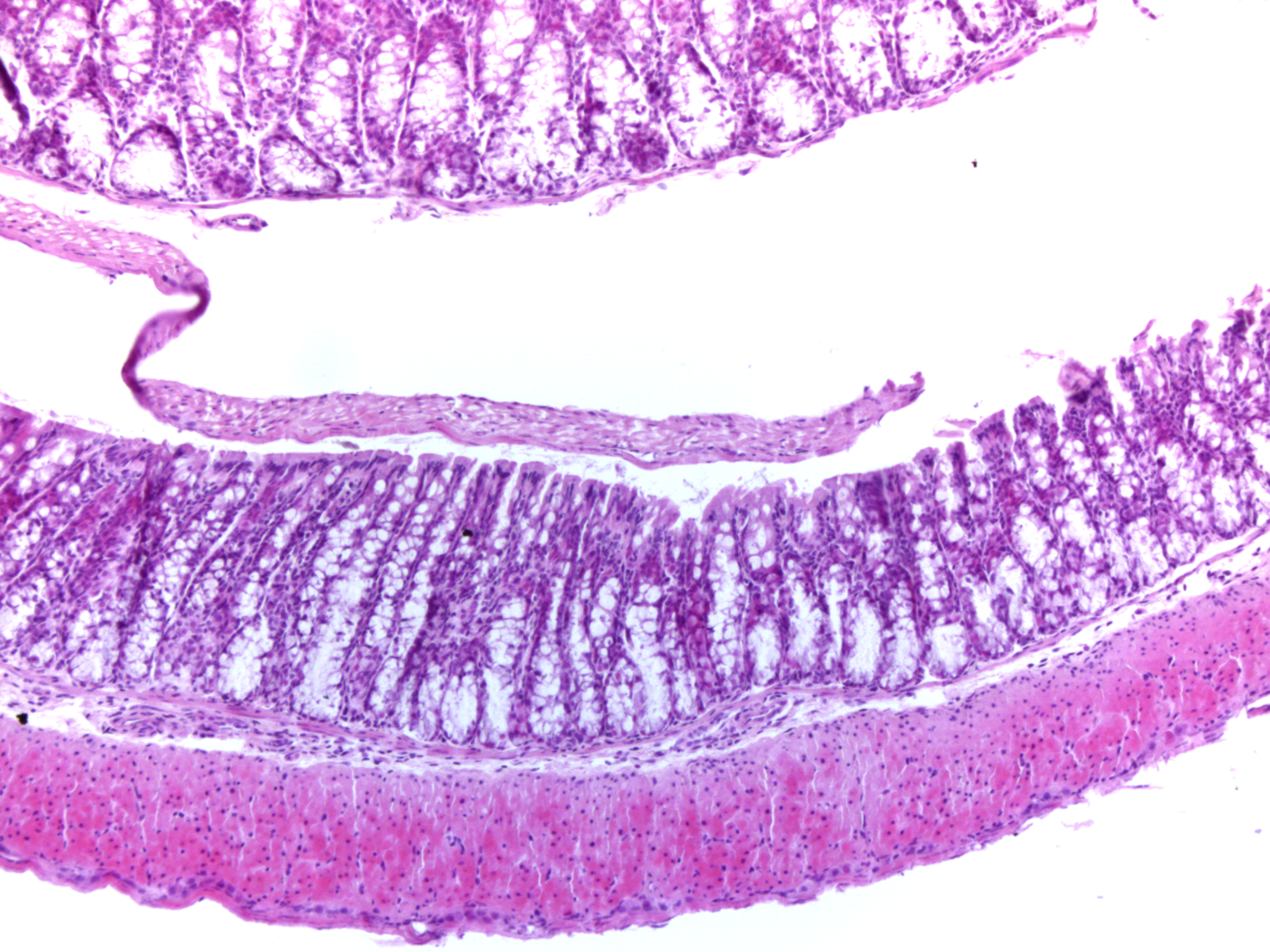

Supplement: Supplementary file 8 — Figure EV1-EV5, Appendix Fig. S1-S4 Source Data [file 44319_2024_186_MOESM8_ESM.zip › EMBOR-2024-59433V3 EV+Appendix Source Data/Appendix Fig S1/S1F/Ctrl.tif]

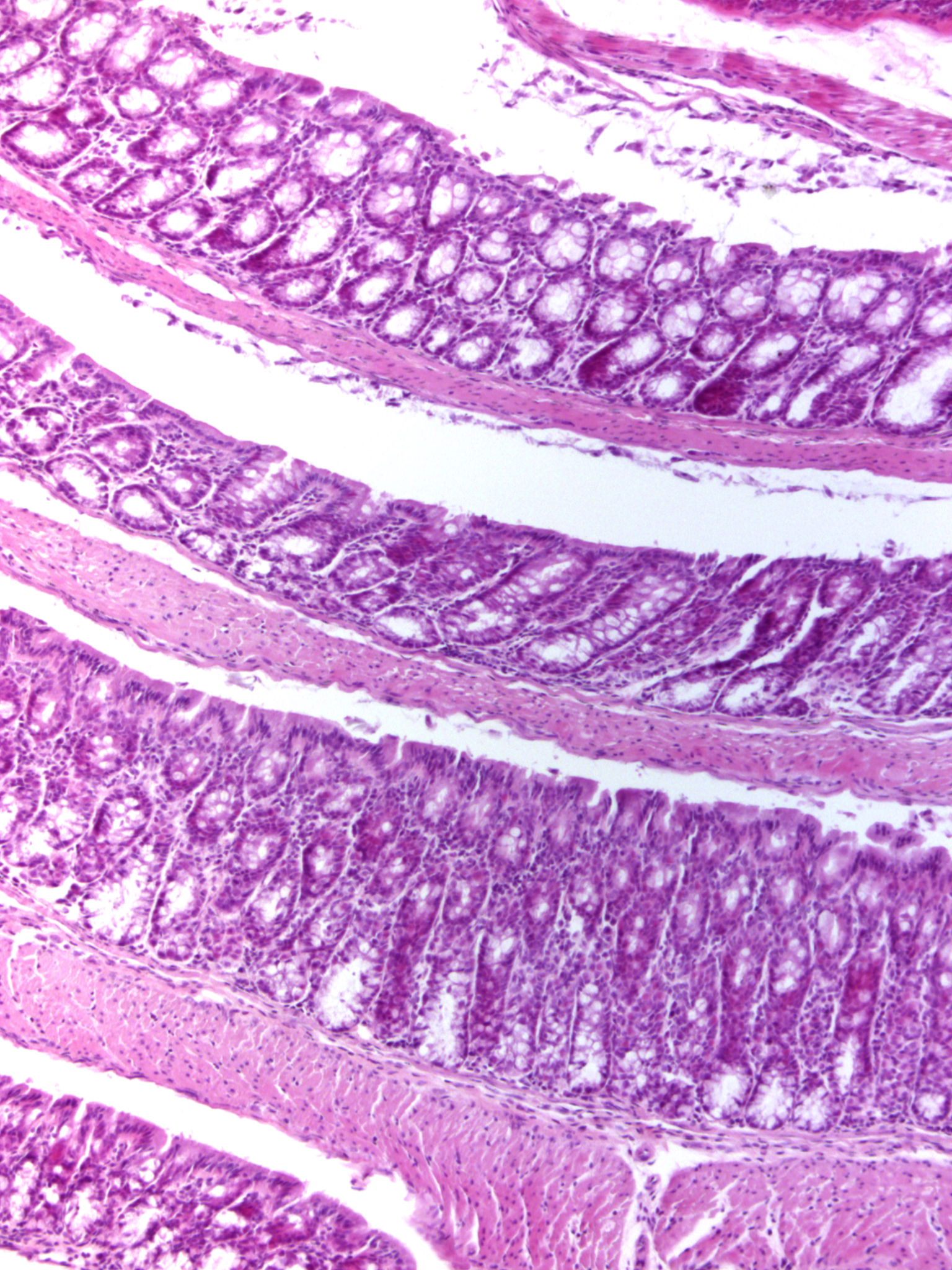

Supplement: Supplementary file 8 — Figure EV1-EV5, Appendix Fig. S1-S4 Source Data [file 44319_2024_186_MOESM8_ESM.zip › EMBOR-2024-59433V3 EV+Appendix Source Data/Appendix Fig S1/S1F/Del.tif]

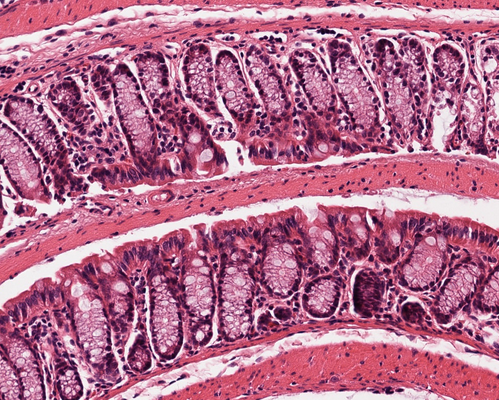

Supplement: Supplementary file 8 — Figure EV1-EV5, Appendix Fig. S1-S4 Source Data [file 44319_2024_186_MOESM8_ESM.zip › EMBOR-2024-59433V3 EV+Appendix Source Data/Appendix Fig S1/S1G/Ctrl IHC HE.png]

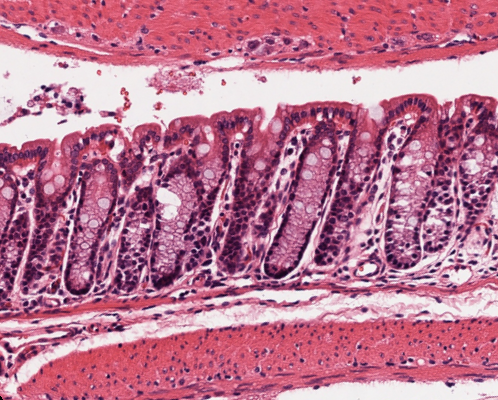

Supplement: Supplementary file 8 — Figure EV1-EV5, Appendix Fig. S1-S4 Source Data [file 44319_2024_186_MOESM8_ESM.zip › EMBOR-2024-59433V3 EV+Appendix Source Data/Appendix Fig S1/S1G/Del IHC HE.png]

## Slide 1
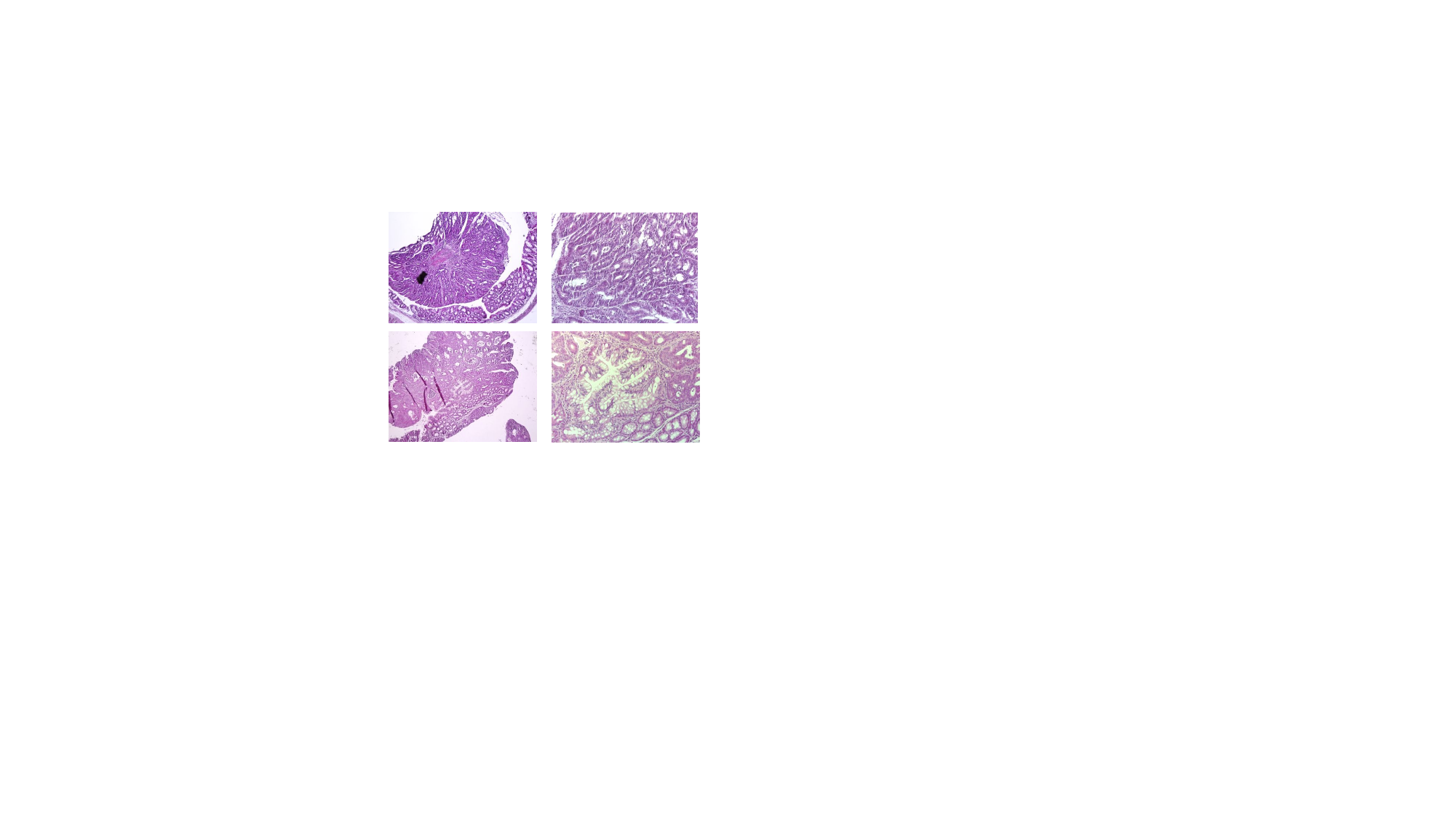

Supplement: Supplementary file 8 — Figure EV1-EV5, Appendix Fig. S1-S4 Source Data [file 44319_2024_186_MOESM8_ESM.zip › EMBOR-2024-59433V3 EV+Appendix Source Data/Appendix Fig S2/S2D/Appendix Fig. S2D image data.pptx]

## Slide 1
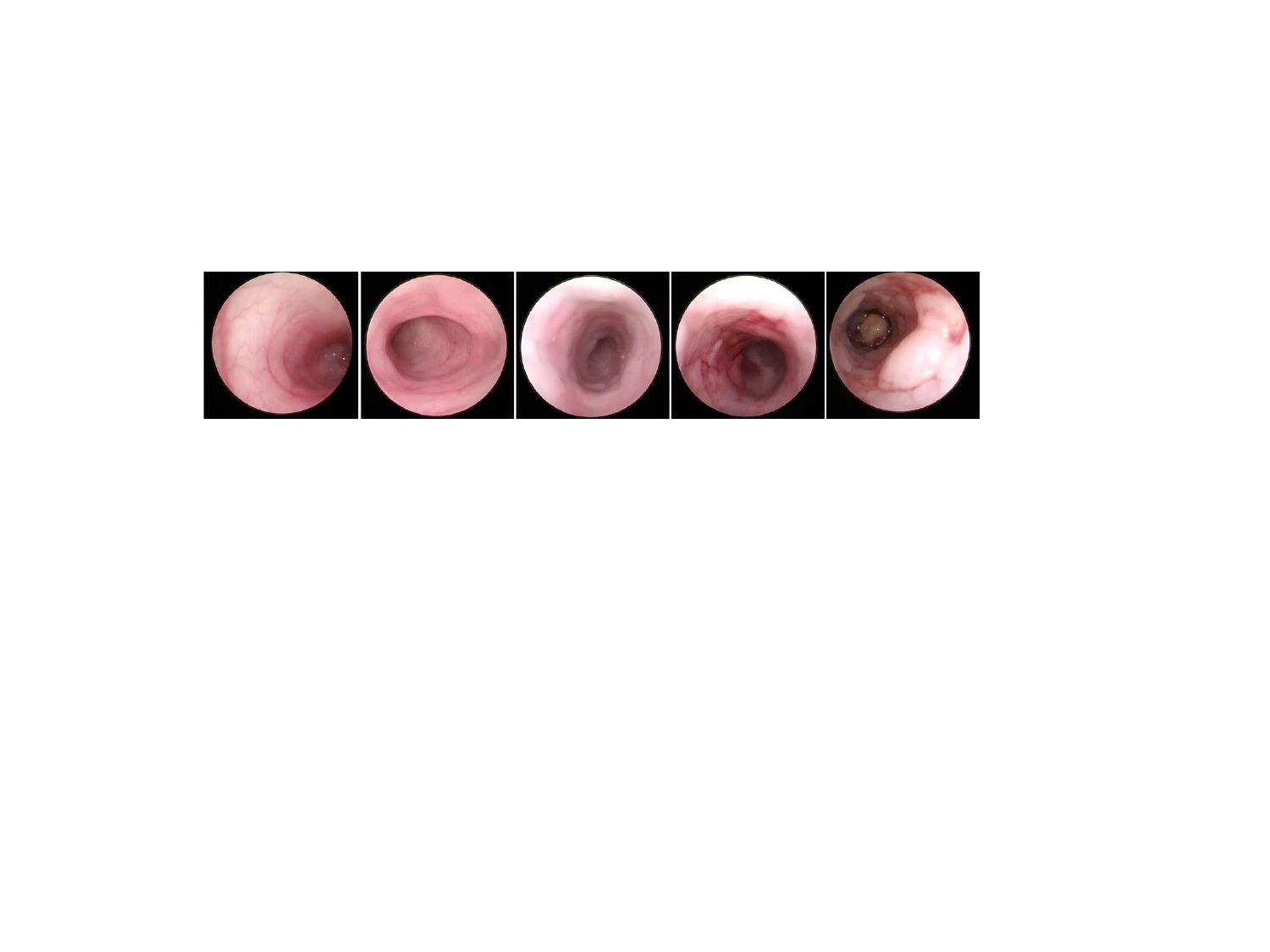

Supplement: Supplementary file 8 — Figure EV1-EV5, Appendix Fig. S1-S4 Source Data [file 44319_2024_186_MOESM8_ESM.zip › EMBOR-2024-59433V3 EV+Appendix Source Data/Appendix Fig S2/S2E/Appendix Fig. S2E image data.pptx]

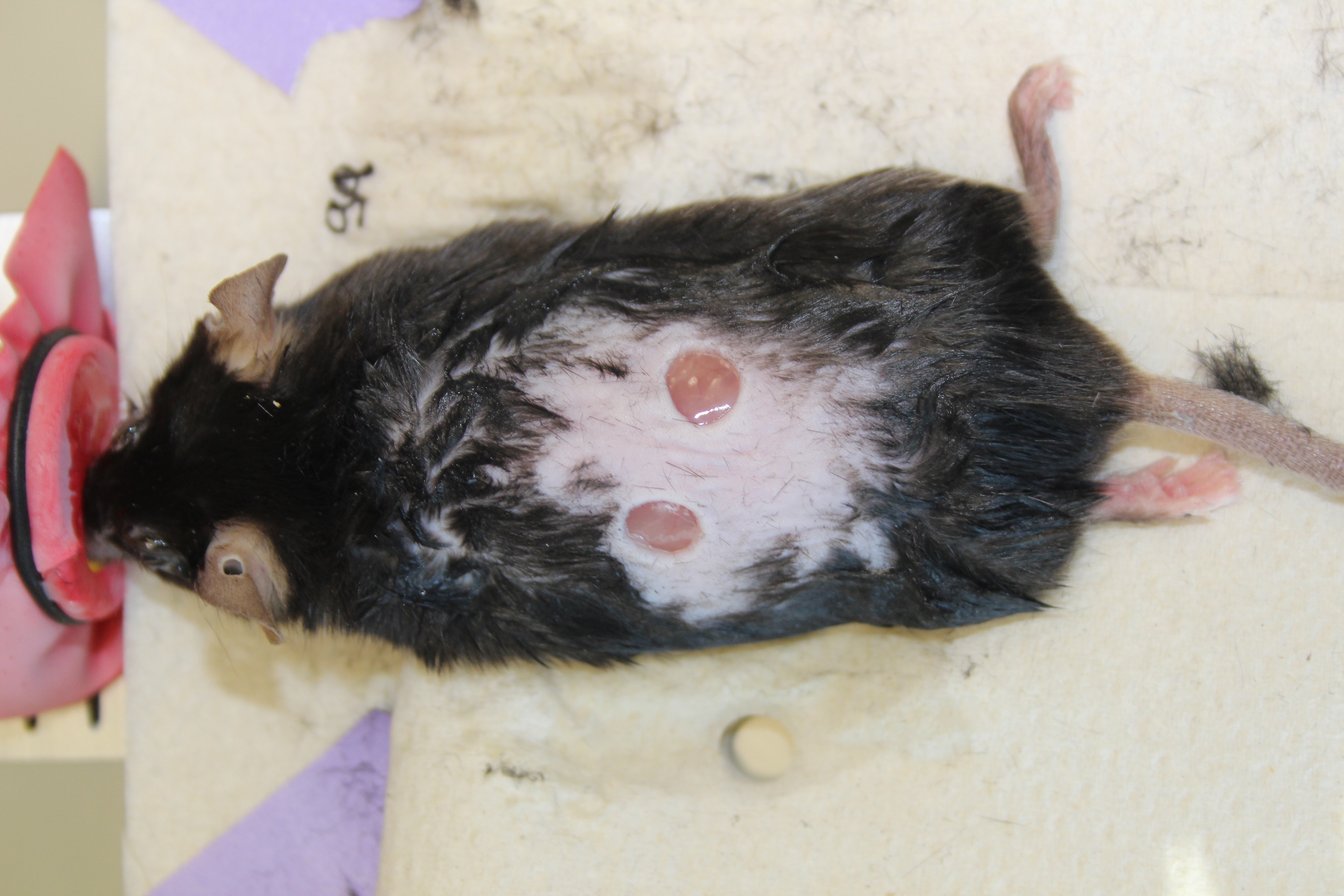

Supplement: Supplementary file 8 — Figure EV1-EV5, Appendix Fig. S1-S4 Source Data [file 44319_2024_186_MOESM8_ESM.zip › EMBOR-2024-59433V3 EV+Appendix Source Data/Appendix Fig S3/S3C/Ctrl d0.JPG]

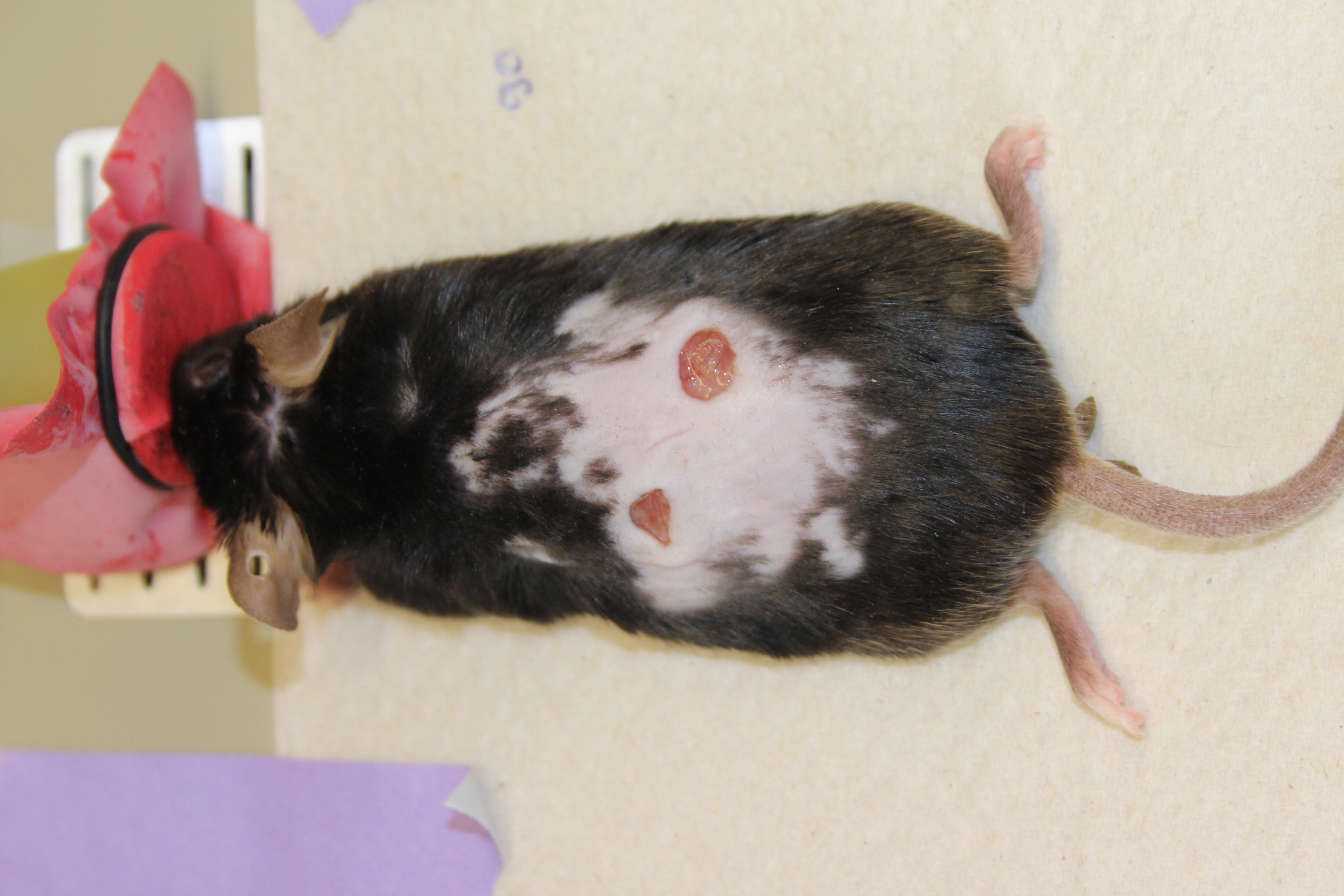

Supplement: Supplementary file 8 — Figure EV1-EV5, Appendix Fig. S1-S4 Source Data [file 44319_2024_186_MOESM8_ESM.zip › EMBOR-2024-59433V3 EV+Appendix Source Data/Appendix Fig S3/S3C/Ctrl d1.JPG]

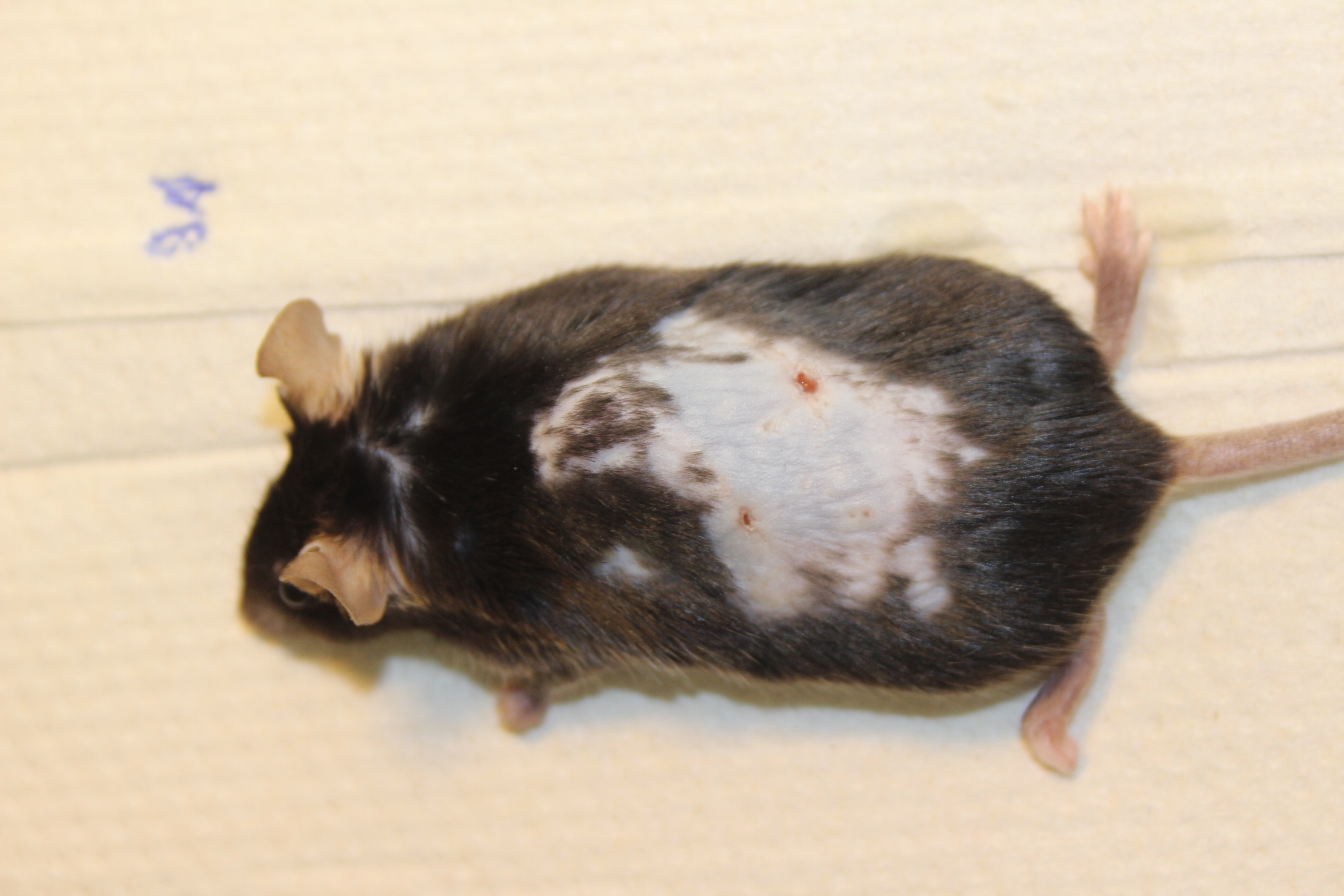

Supplement: Supplementary file 8 — Figure EV1-EV5, Appendix Fig. S1-S4 Source Data [file 44319_2024_186_MOESM8_ESM.zip › EMBOR-2024-59433V3 EV+Appendix Source Data/Appendix Fig S3/S3C/Ctrl d10.JPG]

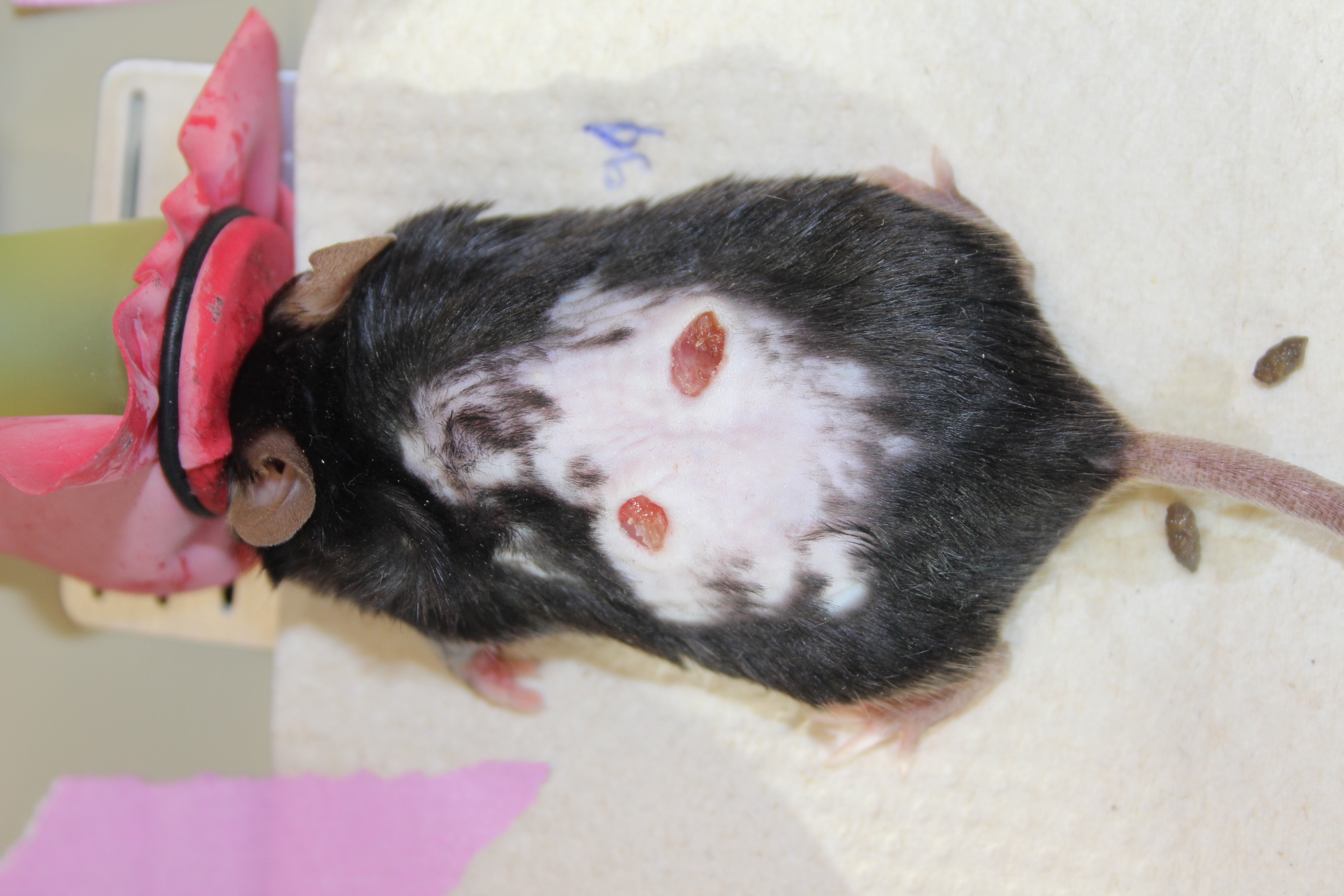

Supplement: Supplementary file 8 — Figure EV1-EV5, Appendix Fig. S1-S4 Source Data [file 44319_2024_186_MOESM8_ESM.zip › EMBOR-2024-59433V3 EV+Appendix Source Data/Appendix Fig S3/S3C/Ctrl d2.JPG]

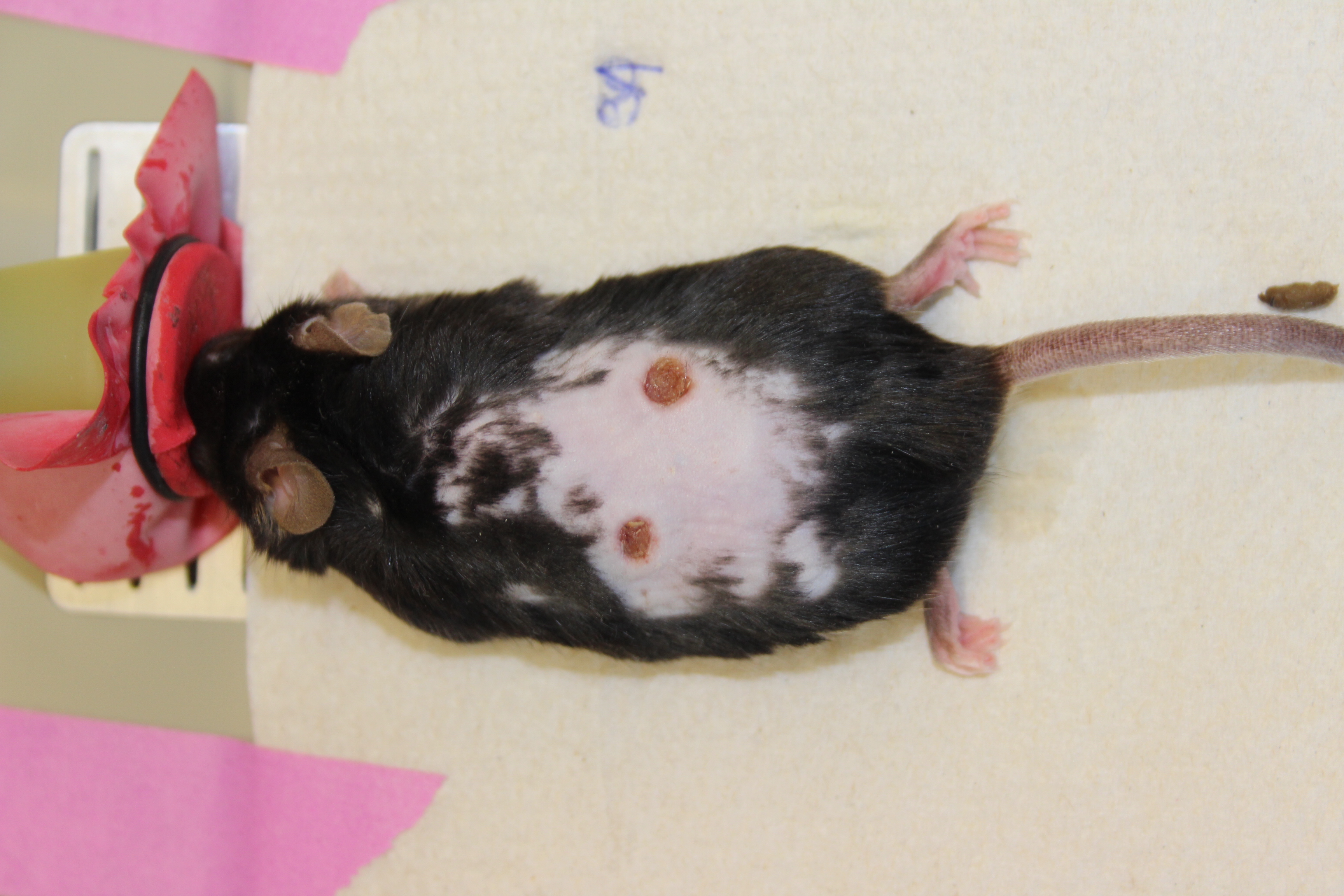

Supplement: Supplementary file 8 — Figure EV1-EV5, Appendix Fig. S1-S4 Source Data [file 44319_2024_186_MOESM8_ESM.zip › EMBOR-2024-59433V3 EV+Appendix Source Data/Appendix Fig S3/S3C/Ctrl d3.JPG]

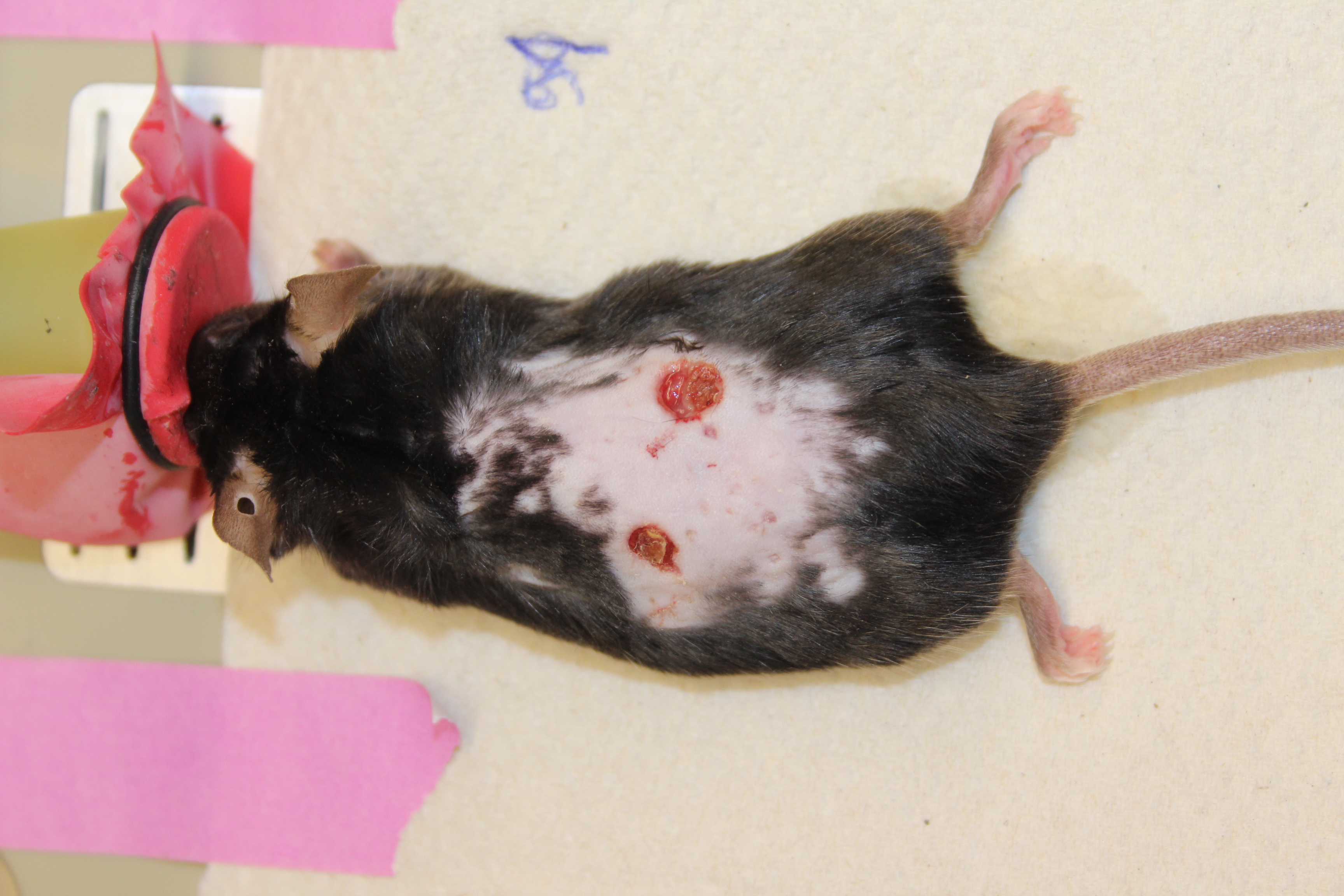

Supplement: Supplementary file 8 — Figure EV1-EV5, Appendix Fig. S1-S4 Source Data [file 44319_2024_186_MOESM8_ESM.zip › EMBOR-2024-59433V3 EV+Appendix Source Data/Appendix Fig S3/S3C/Ctrl d4.JPG]

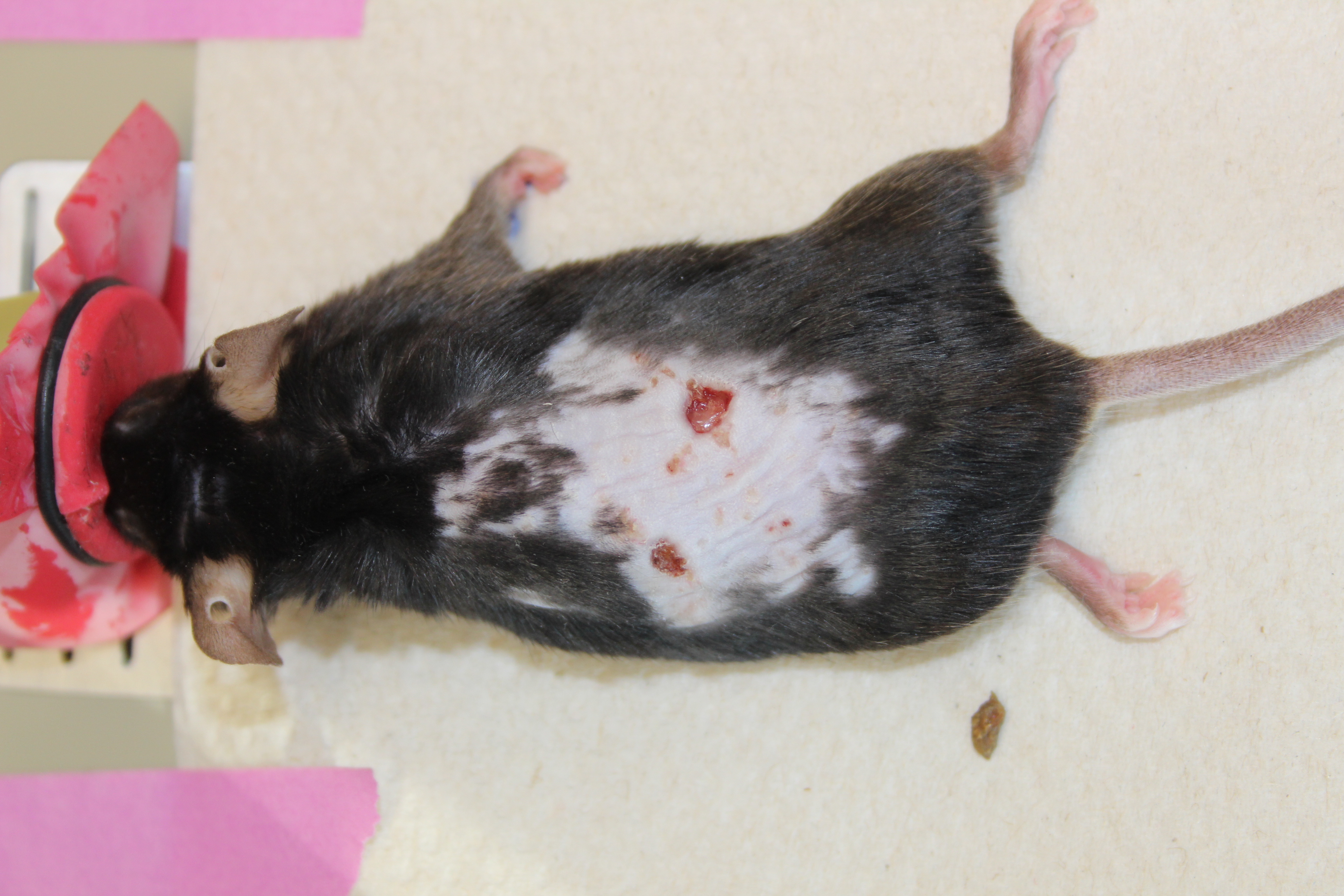

Supplement: Supplementary file 8 — Figure EV1-EV5, Appendix Fig. S1-S4 Source Data [file 44319_2024_186_MOESM8_ESM.zip › EMBOR-2024-59433V3 EV+Appendix Source Data/Appendix Fig S3/S3C/Ctrl d7.JPG]

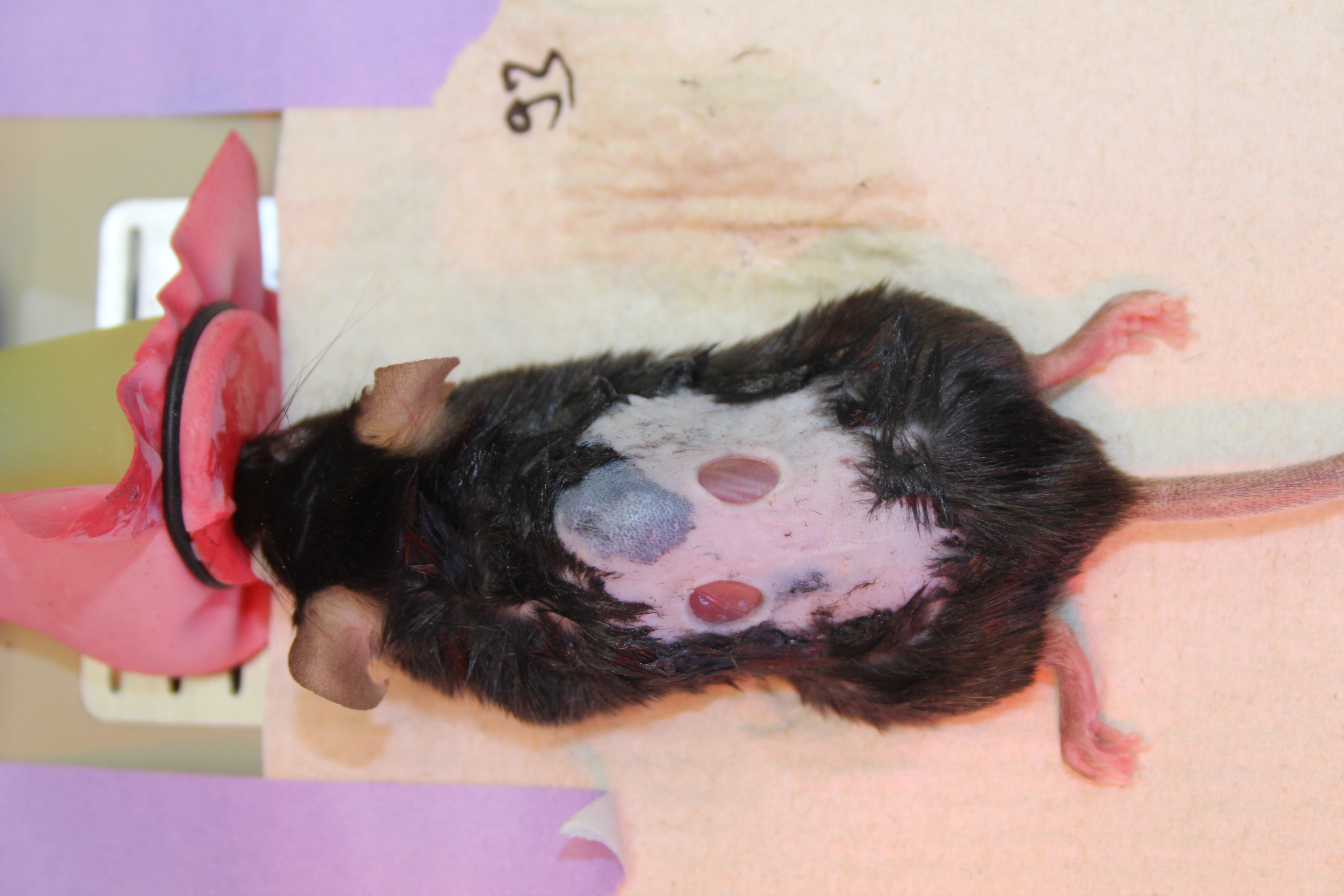

Supplement: Supplementary file 8 — Figure EV1-EV5, Appendix Fig. S1-S4 Source Data [file 44319_2024_186_MOESM8_ESM.zip › EMBOR-2024-59433V3 EV+Appendix Source Data/Appendix Fig S3/S3C/Del d0.JPG]

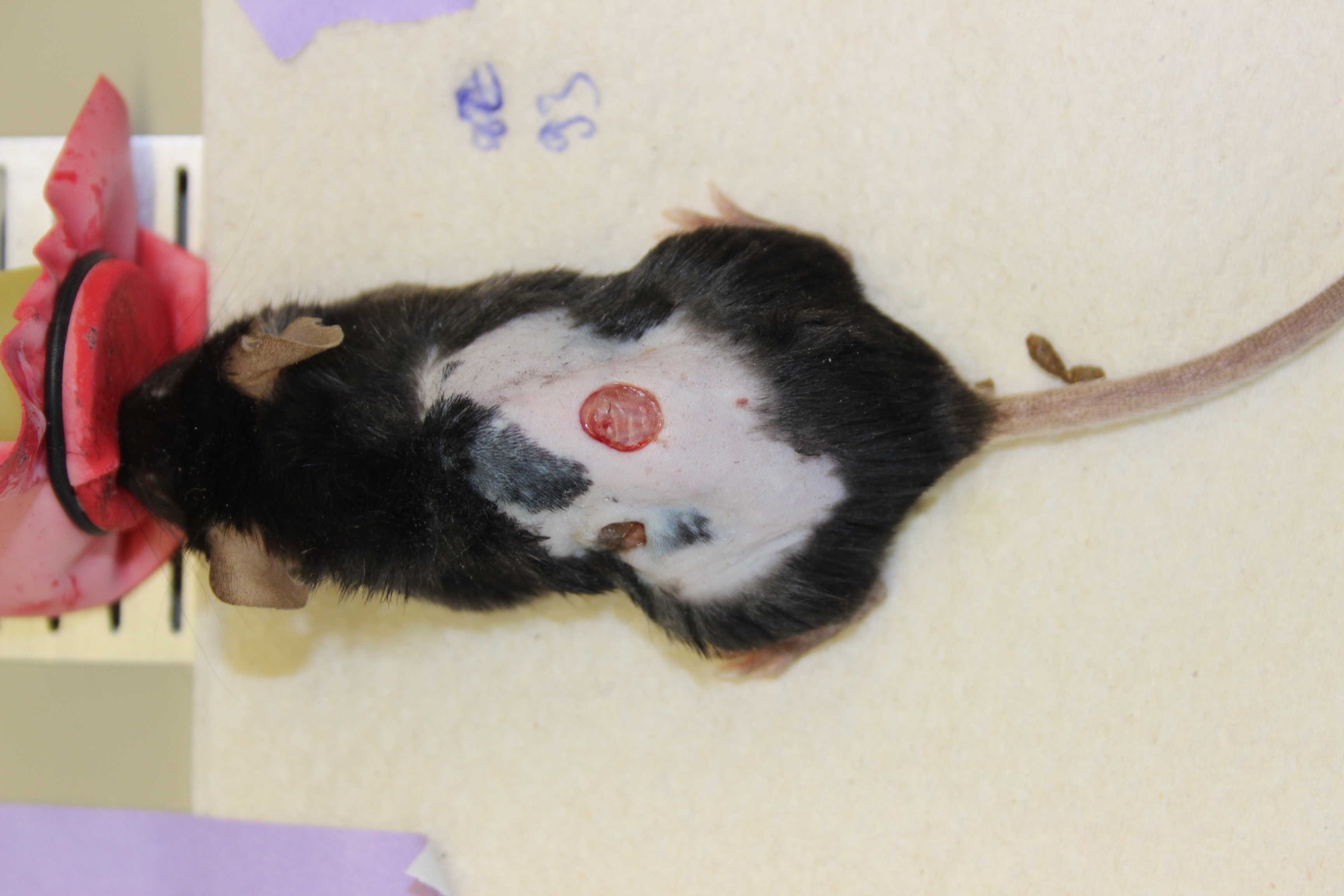

Supplement: Supplementary file 8 — Figure EV1-EV5, Appendix Fig. S1-S4 Source Data [file 44319_2024_186_MOESM8_ESM.zip › EMBOR-2024-59433V3 EV+Appendix Source Data/Appendix Fig S3/S3C/Del d1.JPG]

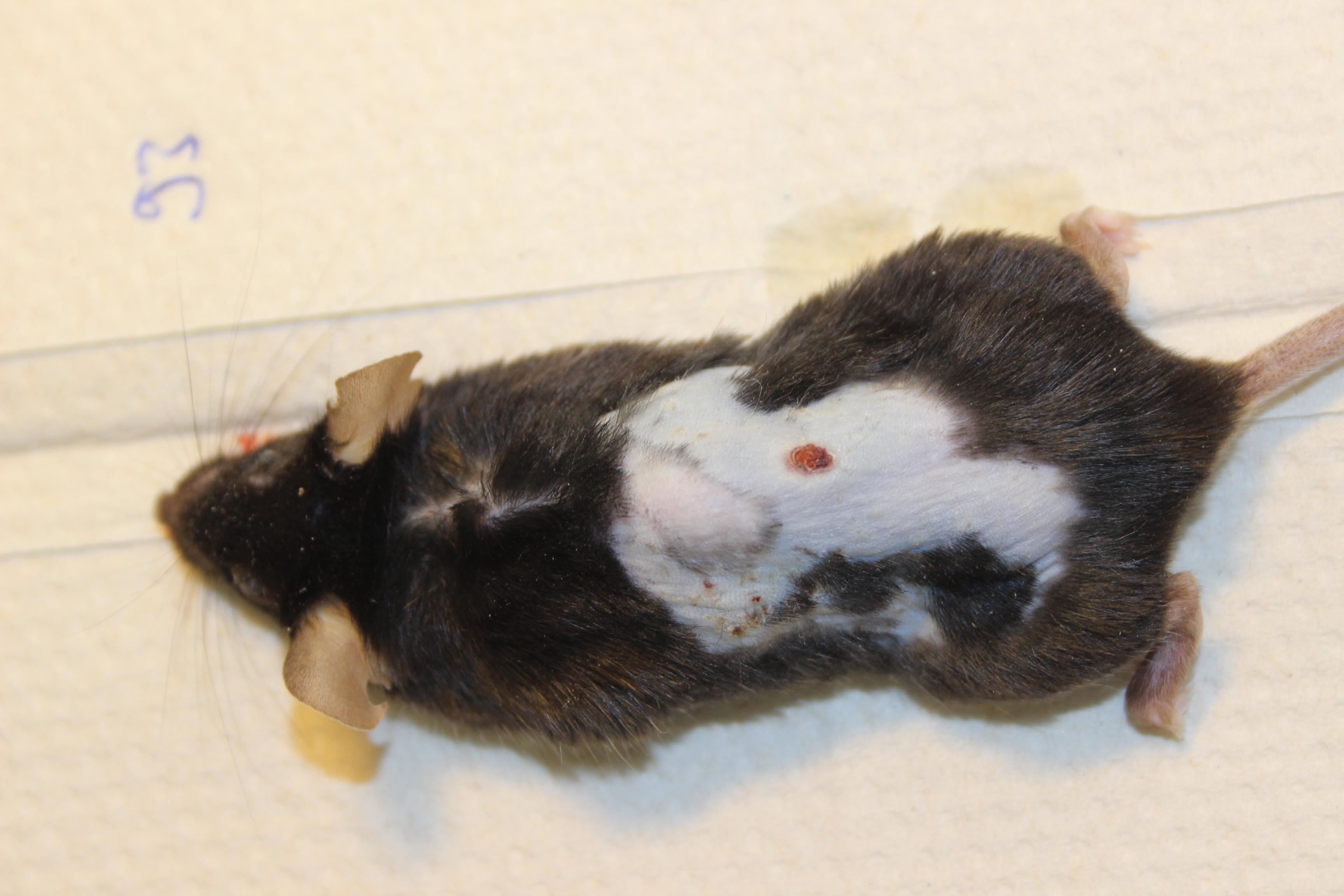

Supplement: Supplementary file 8 — Figure EV1-EV5, Appendix Fig. S1-S4 Source Data [file 44319_2024_186_MOESM8_ESM.zip › EMBOR-2024-59433V3 EV+Appendix Source Data/Appendix Fig S3/S3C/Del d10.JPG]

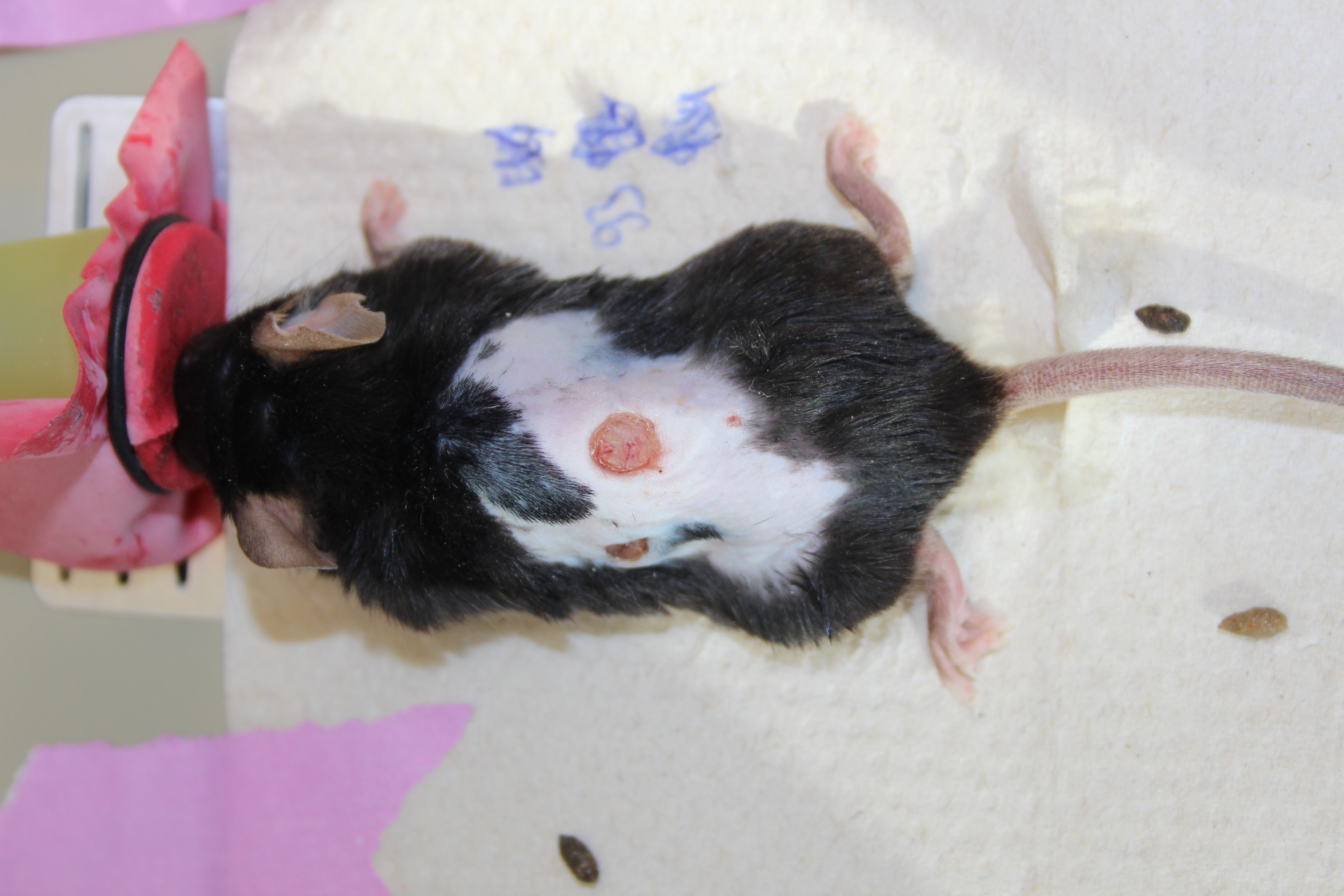

Supplement: Supplementary file 8 — Figure EV1-EV5, Appendix Fig. S1-S4 Source Data [file 44319_2024_186_MOESM8_ESM.zip › EMBOR-2024-59433V3 EV+Appendix Source Data/Appendix Fig S3/S3C/Del d2.JPG]

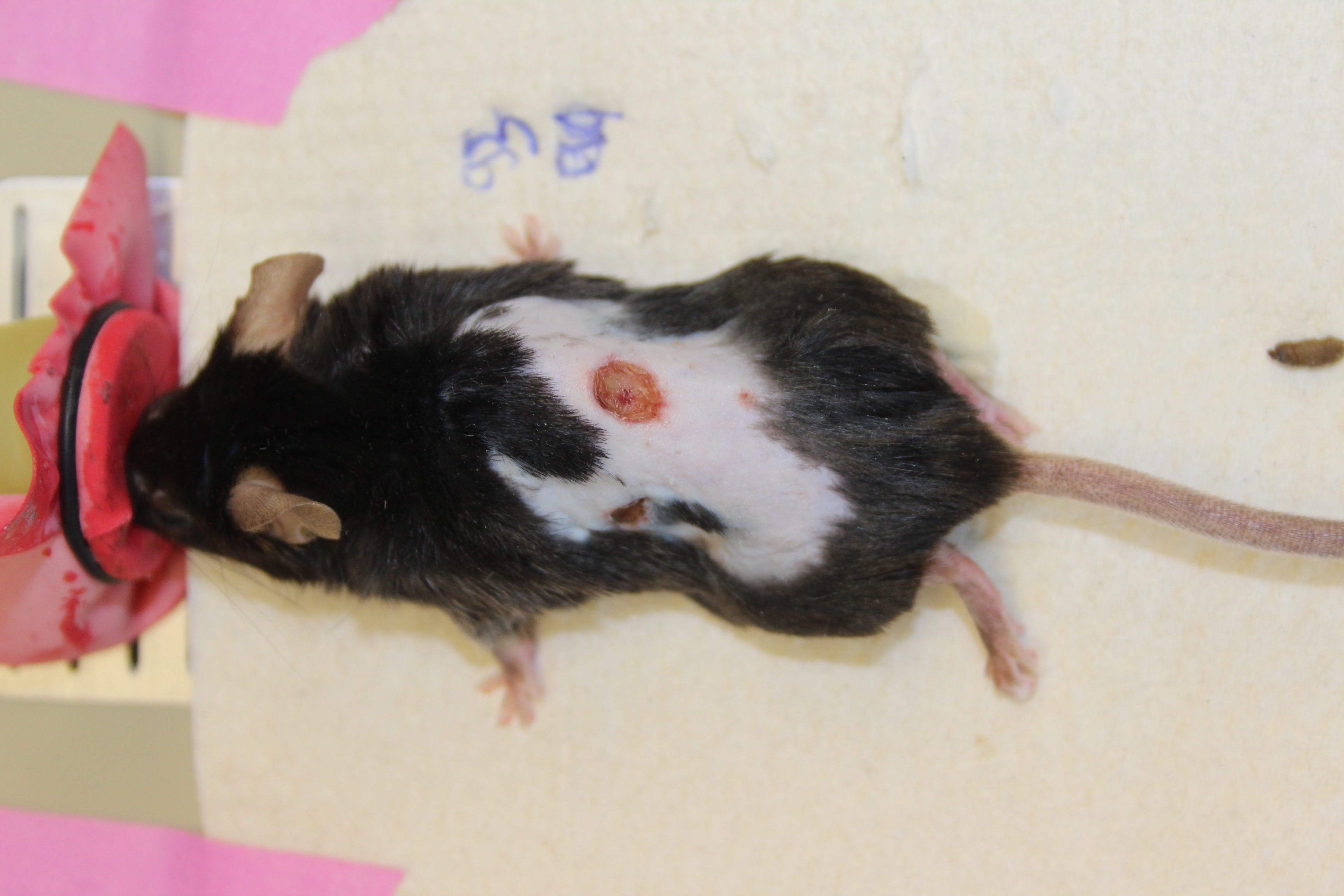

Supplement: Supplementary file 8 — Figure EV1-EV5, Appendix Fig. S1-S4 Source Data [file 44319_2024_186_MOESM8_ESM.zip › EMBOR-2024-59433V3 EV+Appendix Source Data/Appendix Fig S3/S3C/Del d3.JPG]

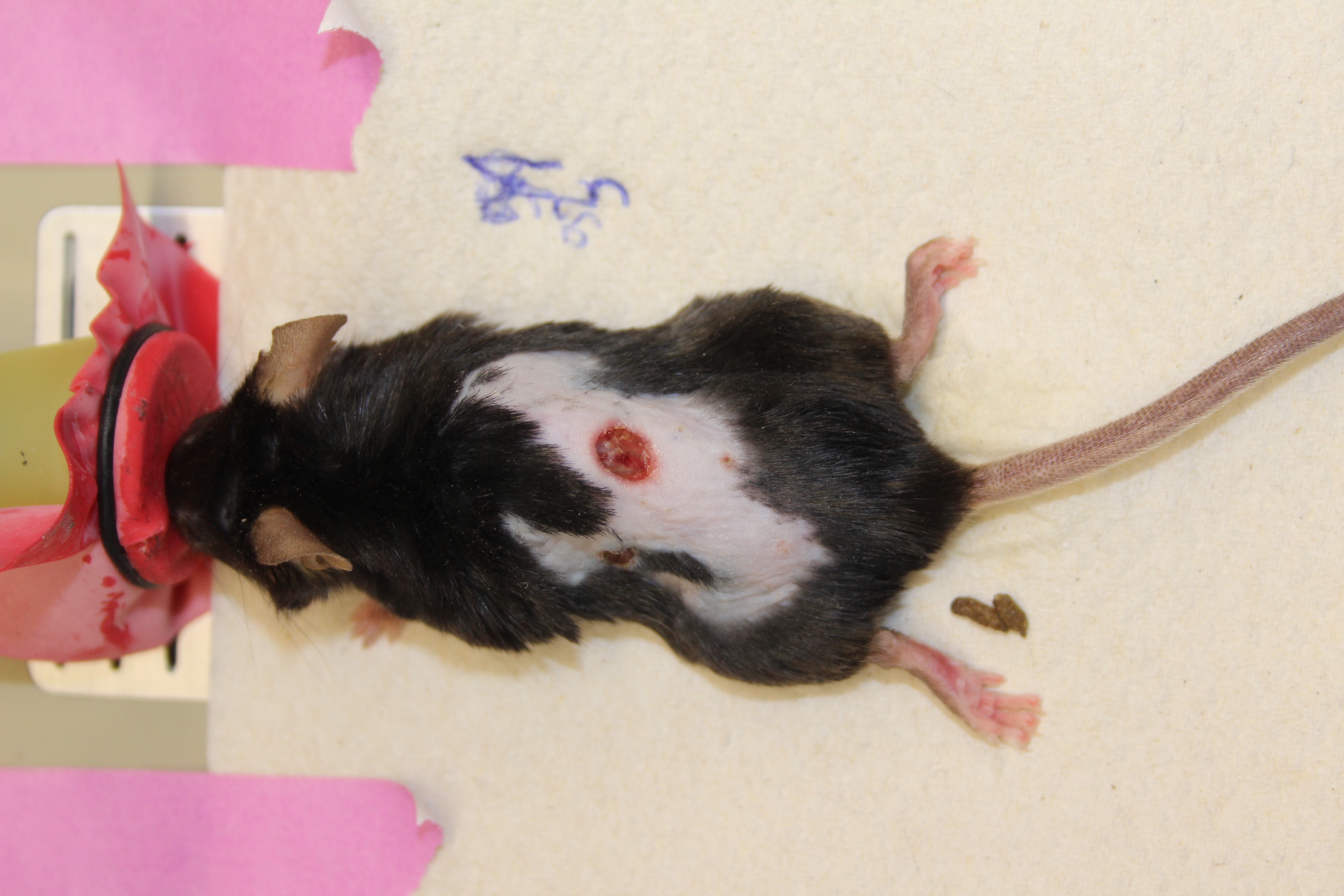

Supplement: Supplementary file 8 — Figure EV1-EV5, Appendix Fig. S1-S4 Source Data [file 44319_2024_186_MOESM8_ESM.zip › EMBOR-2024-59433V3 EV+Appendix Source Data/Appendix Fig S3/S3C/Del d4.JPG]

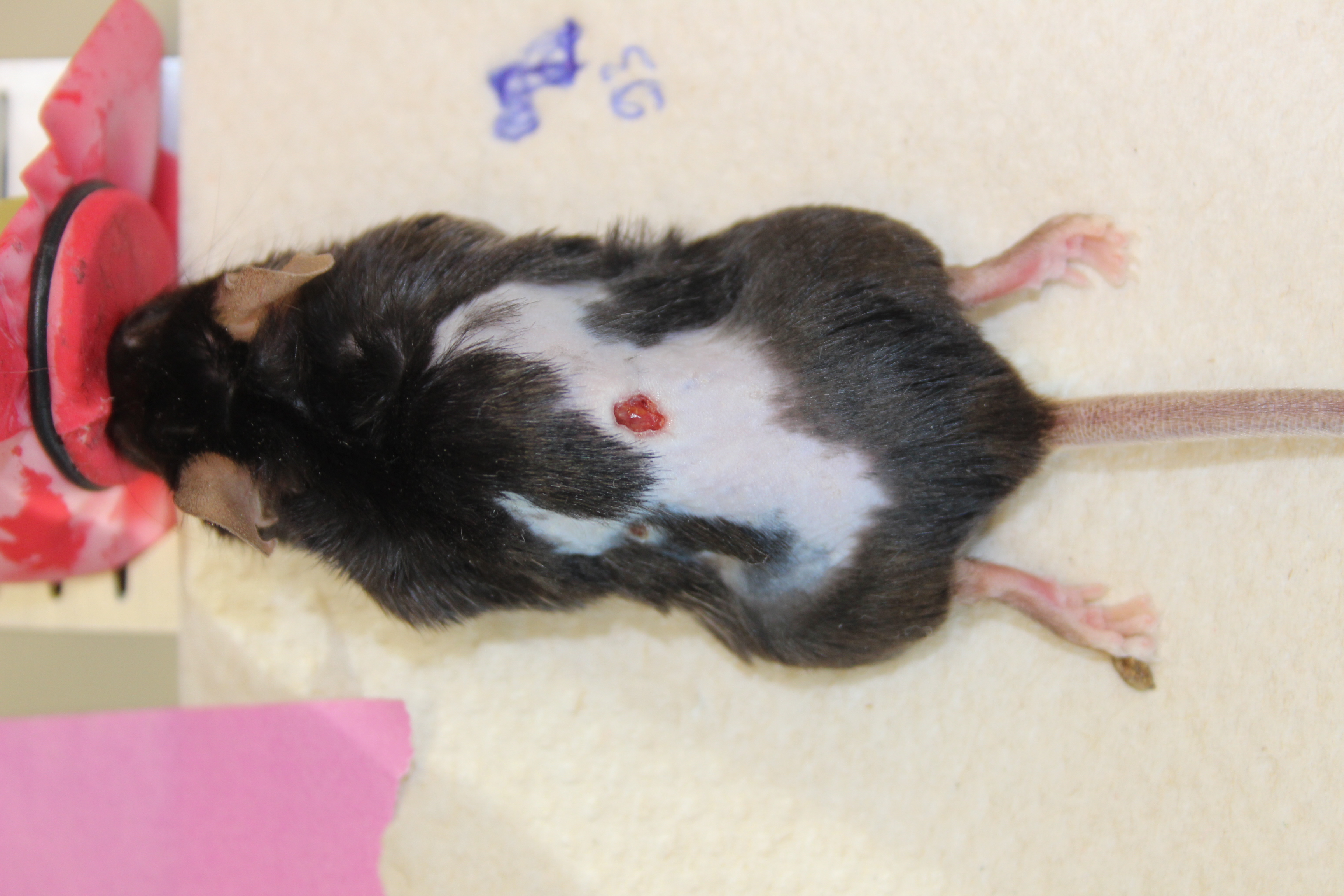

Supplement: Supplementary file 8 — Figure EV1-EV5, Appendix Fig. S1-S4 Source Data [file 44319_2024_186_MOESM8_ESM.zip › EMBOR-2024-59433V3 EV+Appendix Source Data/Appendix Fig S3/S3C/Del d7.JPG]

## Slide 1
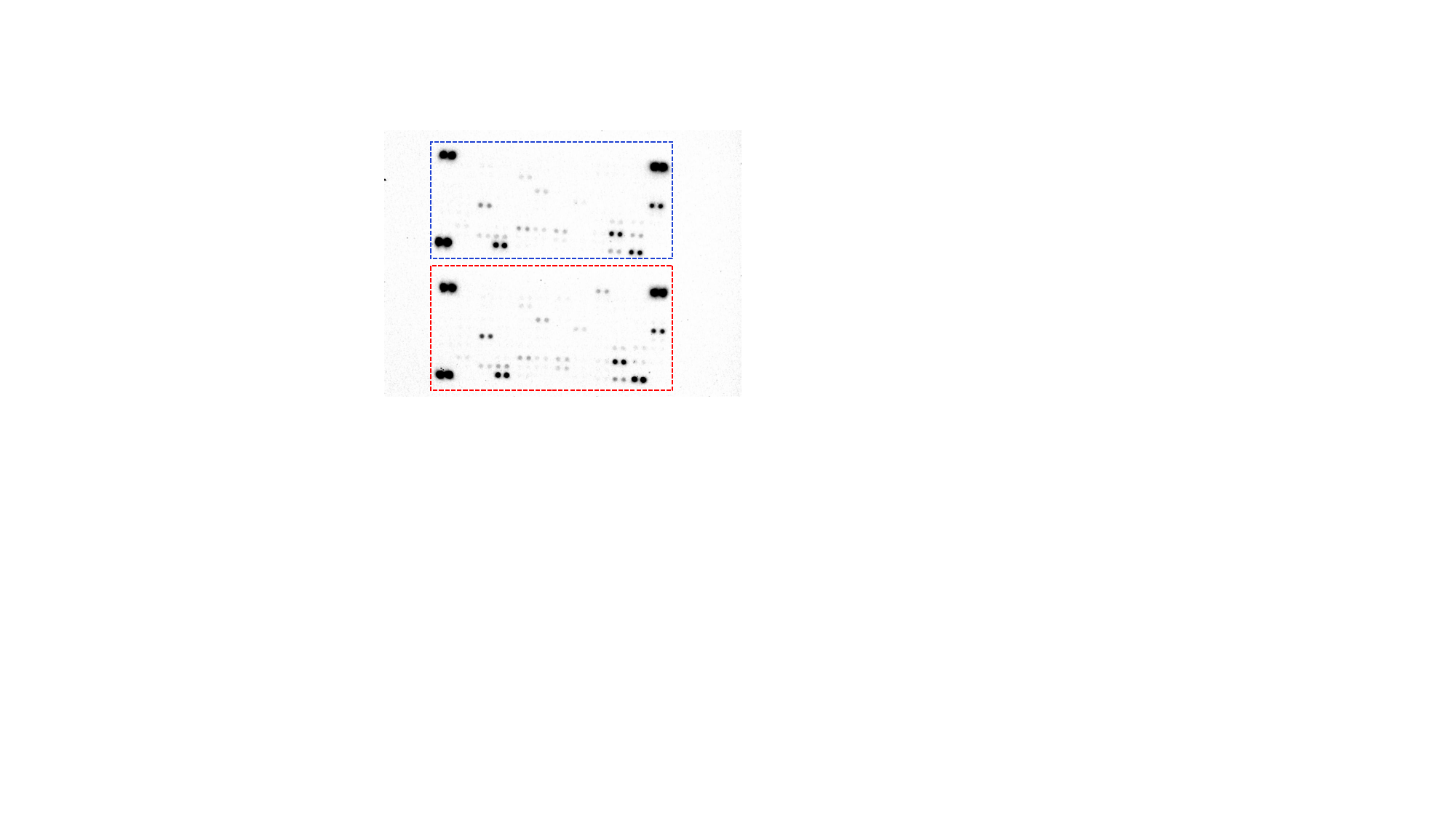

Supplement: Supplementary file 8 — Figure EV1-EV5, Appendix Fig. S1-S4 Source Data [file 44319_2024_186_MOESM8_ESM.zip › EMBOR-2024-59433V3 EV+Appendix Source Data/Appendix Fig S4/S4A/Appendix Fig. S4A image data.pptx]

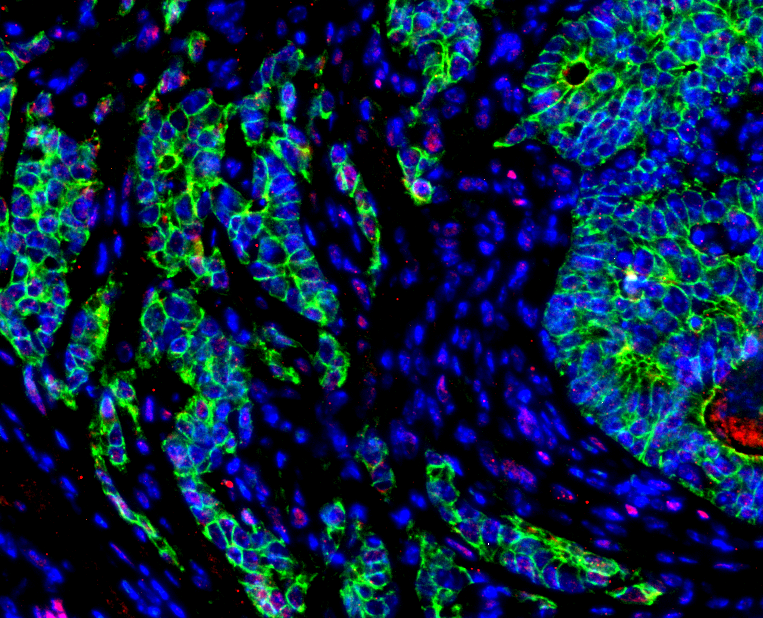

Supplement: Supplementary file 8 — Figure EV1-EV5, Appendix Fig. S1-S4 Source Data [file 44319_2024_186_MOESM8_ESM.zip › EMBOR-2024-59433V3 EV+Appendix Source Data/Fig EV 1/1A/Del ZEB1 IF.tif]

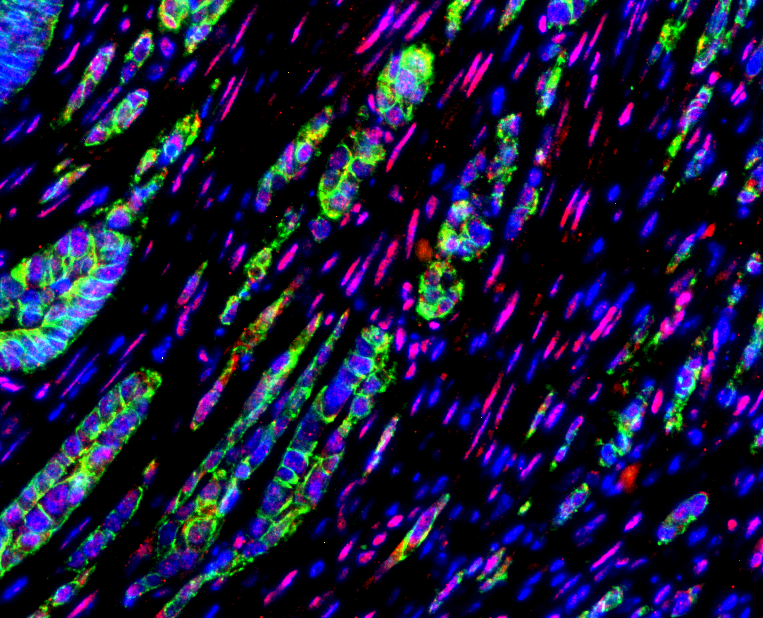

Supplement: Supplementary file 8 — Figure EV1-EV5, Appendix Fig. S1-S4 Source Data [file 44319_2024_186_MOESM8_ESM.zip › EMBOR-2024-59433V3 EV+Appendix Source Data/Fig EV 1/1A/WT ZEB1 IF.tif]

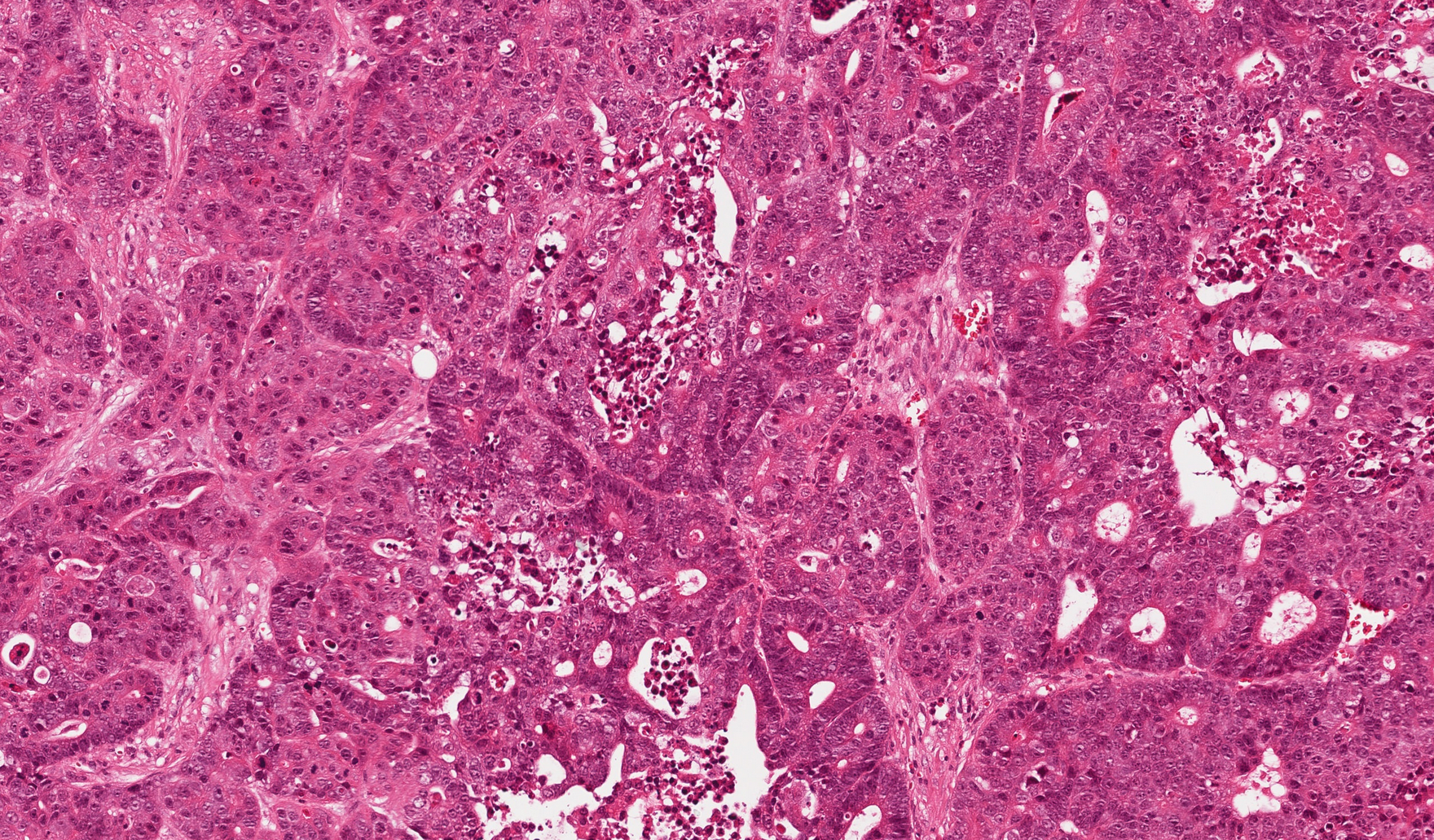

Supplement: Supplementary file 8 — Figure EV1-EV5, Appendix Fig. S1-S4 Source Data [file 44319_2024_186_MOESM8_ESM.zip › EMBOR-2024-59433V3 EV+Appendix Source Data/Fig EV 1/1D/Ctrl IHC HE.png]

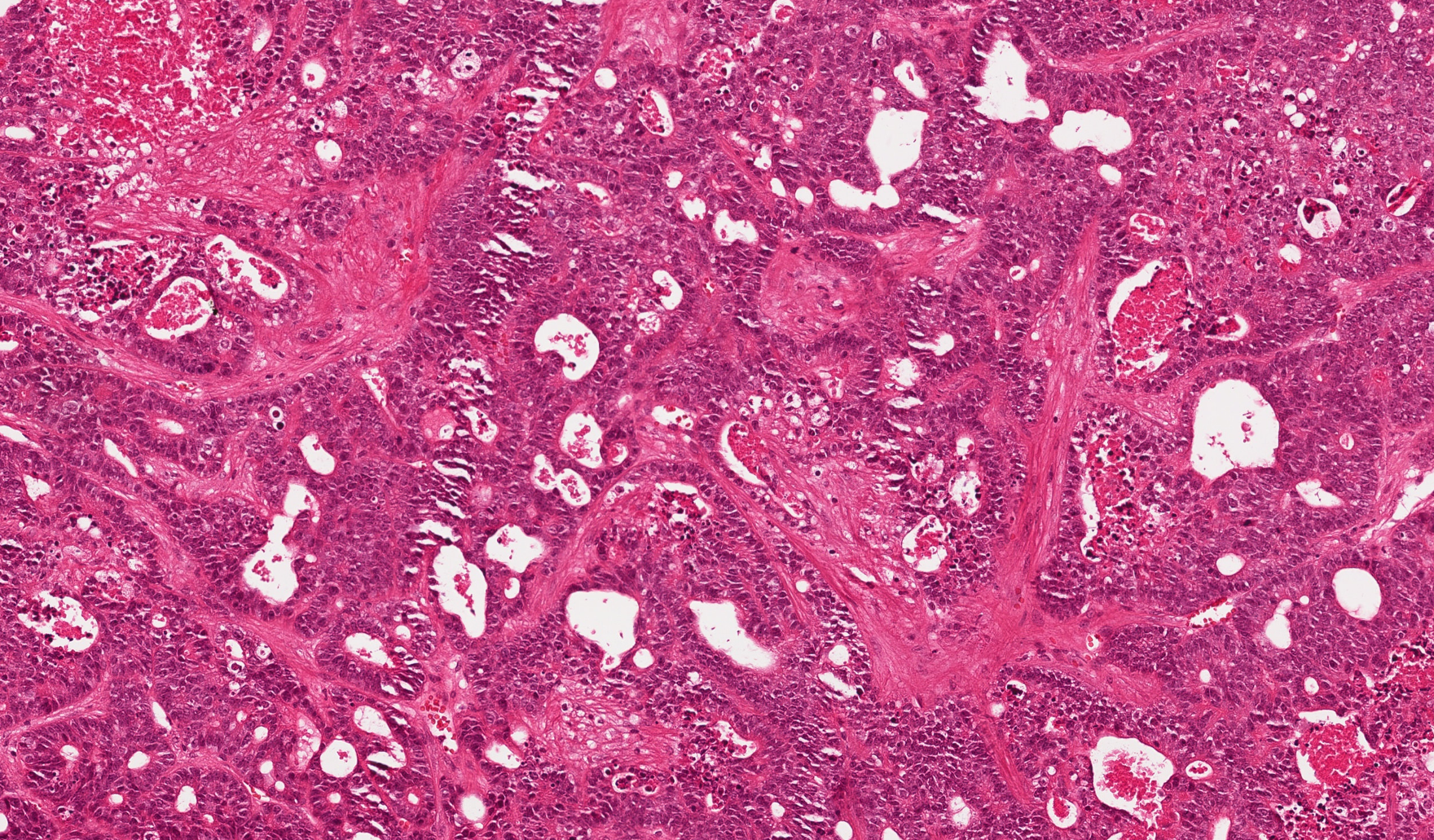

Supplement: Supplementary file 8 — Figure EV1-EV5, Appendix Fig. S1-S4 Source Data [file 44319_2024_186_MOESM8_ESM.zip › EMBOR-2024-59433V3 EV+Appendix Source Data/Fig EV 1/1D/Del IHC HE.png]

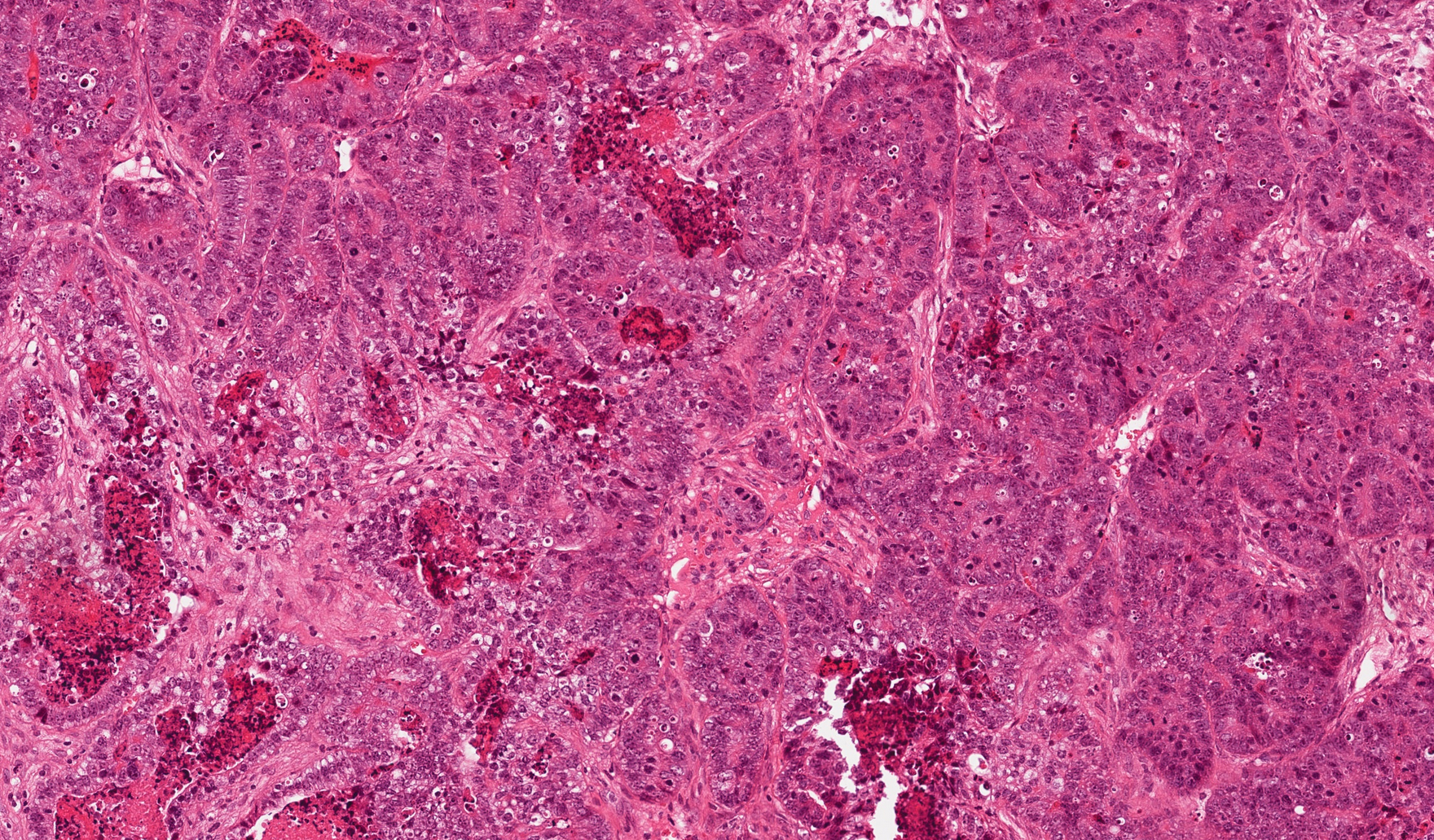

Supplement: Supplementary file 8 — Figure EV1-EV5, Appendix Fig. S1-S4 Source Data [file 44319_2024_186_MOESM8_ESM.zip › EMBOR-2024-59433V3 EV+Appendix Source Data/Fig EV 1/1E/Ctrl IHC HE.png]

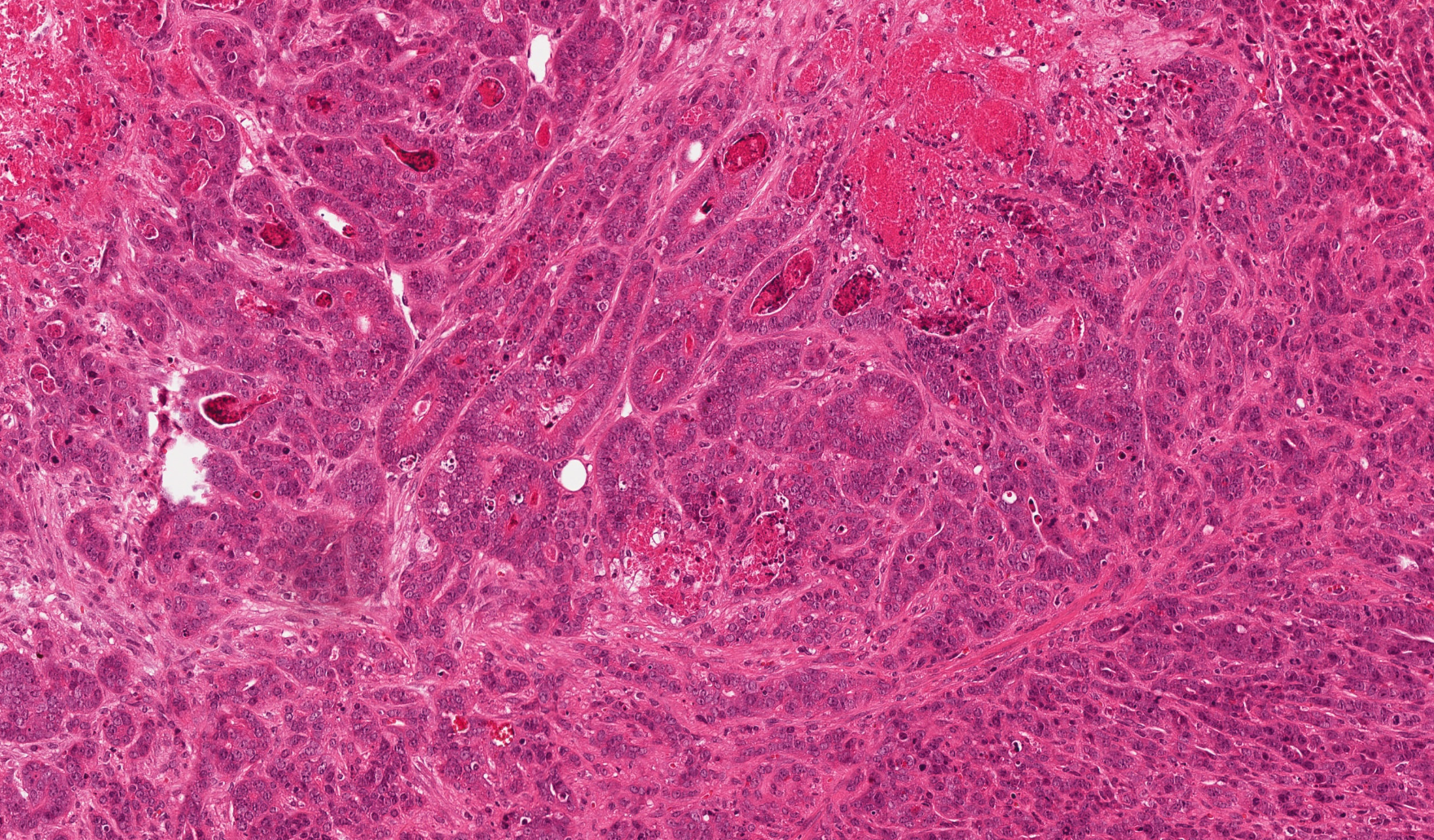

Supplement: Supplementary file 8 — Figure EV1-EV5, Appendix Fig. S1-S4 Source Data [file 44319_2024_186_MOESM8_ESM.zip › EMBOR-2024-59433V3 EV+Appendix Source Data/Fig EV 1/1E/Del IHC HE.png]

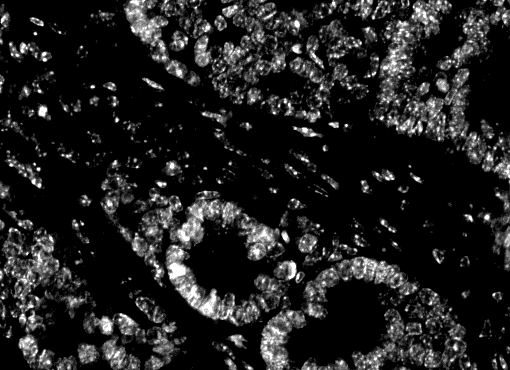

Supplement: Supplementary file 8 — Figure EV1-EV5, Appendix Fig. S1-S4 Source Data [file 44319_2024_186_MOESM8_ESM.zip › EMBOR-2024-59433V3 EV+Appendix Source Data/Fig EV 3/3A/Ctrl IF DAPI.tif]

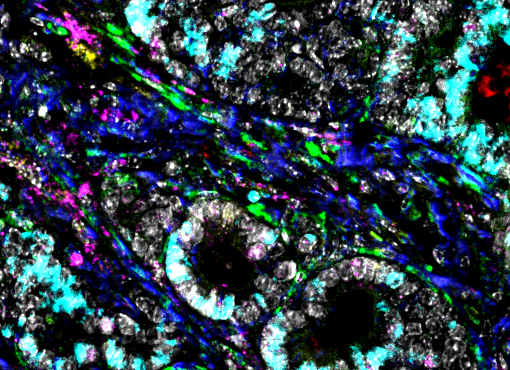

Supplement: Supplementary file 8 — Figure EV1-EV5, Appendix Fig. S1-S4 Source Data [file 44319_2024_186_MOESM8_ESM.zip › EMBOR-2024-59433V3 EV+Appendix Source Data/Fig EV 3/3A/Ctrl IF Merge.tif]

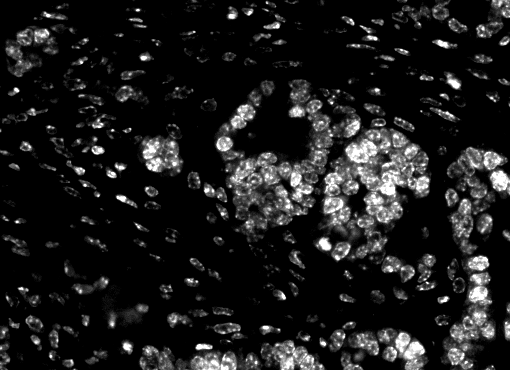

Supplement: Supplementary file 8 — Figure EV1-EV5, Appendix Fig. S1-S4 Source Data [file 44319_2024_186_MOESM8_ESM.zip › EMBOR-2024-59433V3 EV+Appendix Source Data/Fig EV 3/3B/Del IF DAPI.tif]

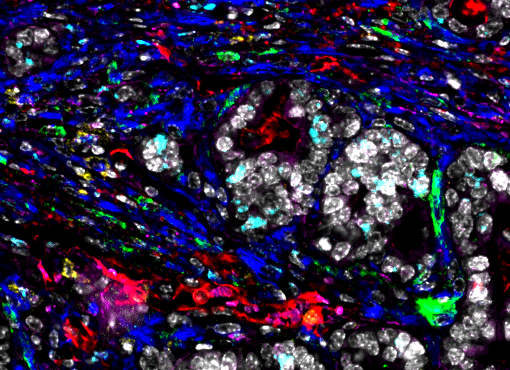

Supplement: Supplementary file 8 — Figure EV1-EV5, Appendix Fig. S1-S4 Source Data [file 44319_2024_186_MOESM8_ESM.zip › EMBOR-2024-59433V3 EV+Appendix Source Data/Fig EV 3/3B/Del IF Merge.tif]

## Slide 1
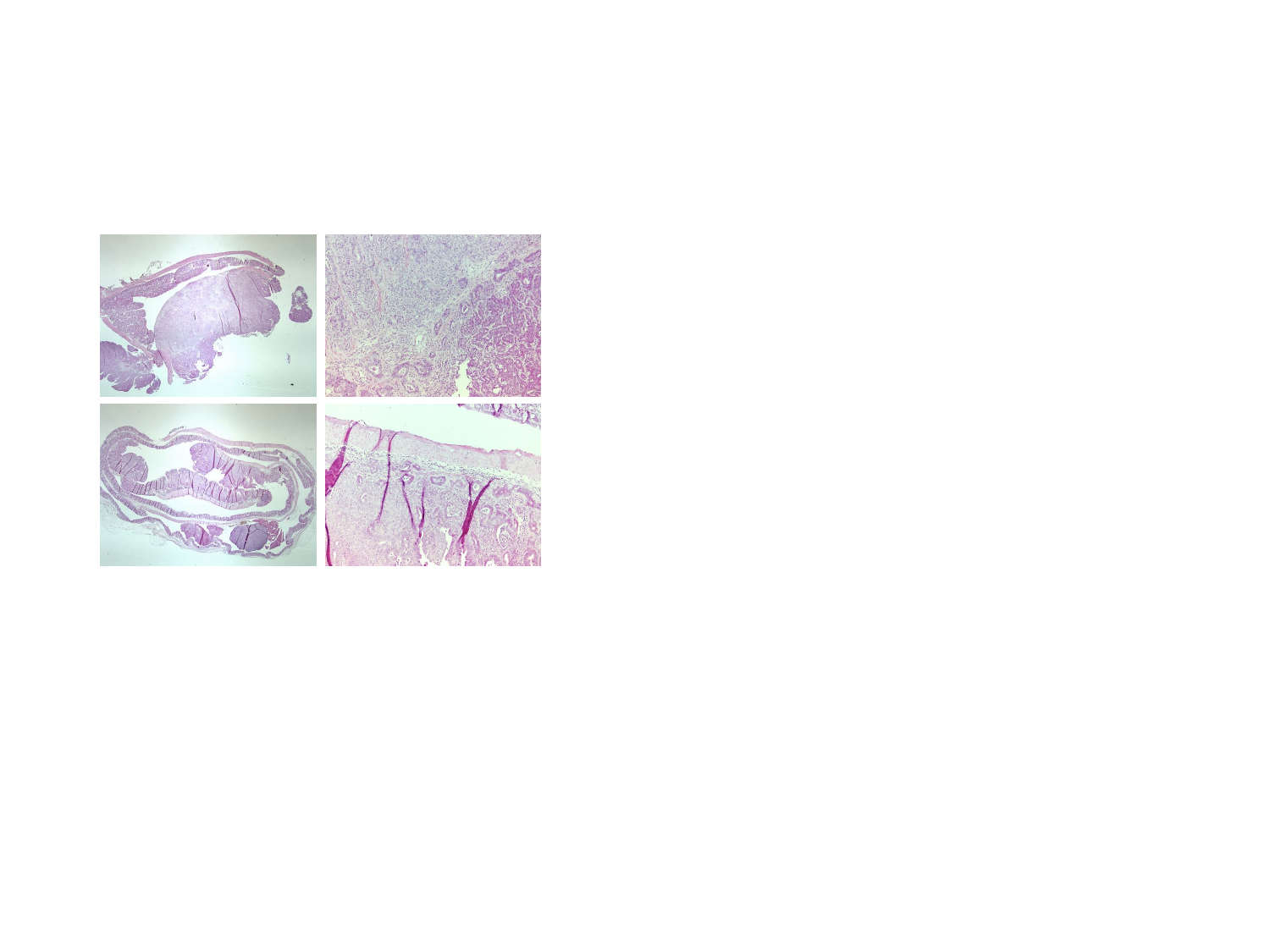

Supplement: Supplementary file 8 — Figure EV1-EV5, Appendix Fig. S1-S4 Source Data [file 44319_2024_186_MOESM8_ESM.zip › EMBOR-2024-59433V3 EV+Appendix Source Data/Fig EV 4/4D/Fig. EV 4D image data.pptx]
