# Supplementary material for: ZEB1-mediated fibroblast polarization controls inflammation and sensitivity to immunotherapy in colorectal cancer
Source: EMBO Rep. 2024 Jun 27;25(8):15. doi: 10.1038/s44319-024-00186-7 (PMC11315988; doi:10.1038/s44319-024-00186-7)
Supplement: Supplementary file 8 — Figure EV1-EV5, Appendix Fig. S1-S4 Source Data [file 44319_2024_186_MOESM8_ESM.zip › EMBOR-2024-59433V3 EV+Appendix Source Data/Appendix Fig S1/S1C/agarose gels.pptx]

## Slide 1
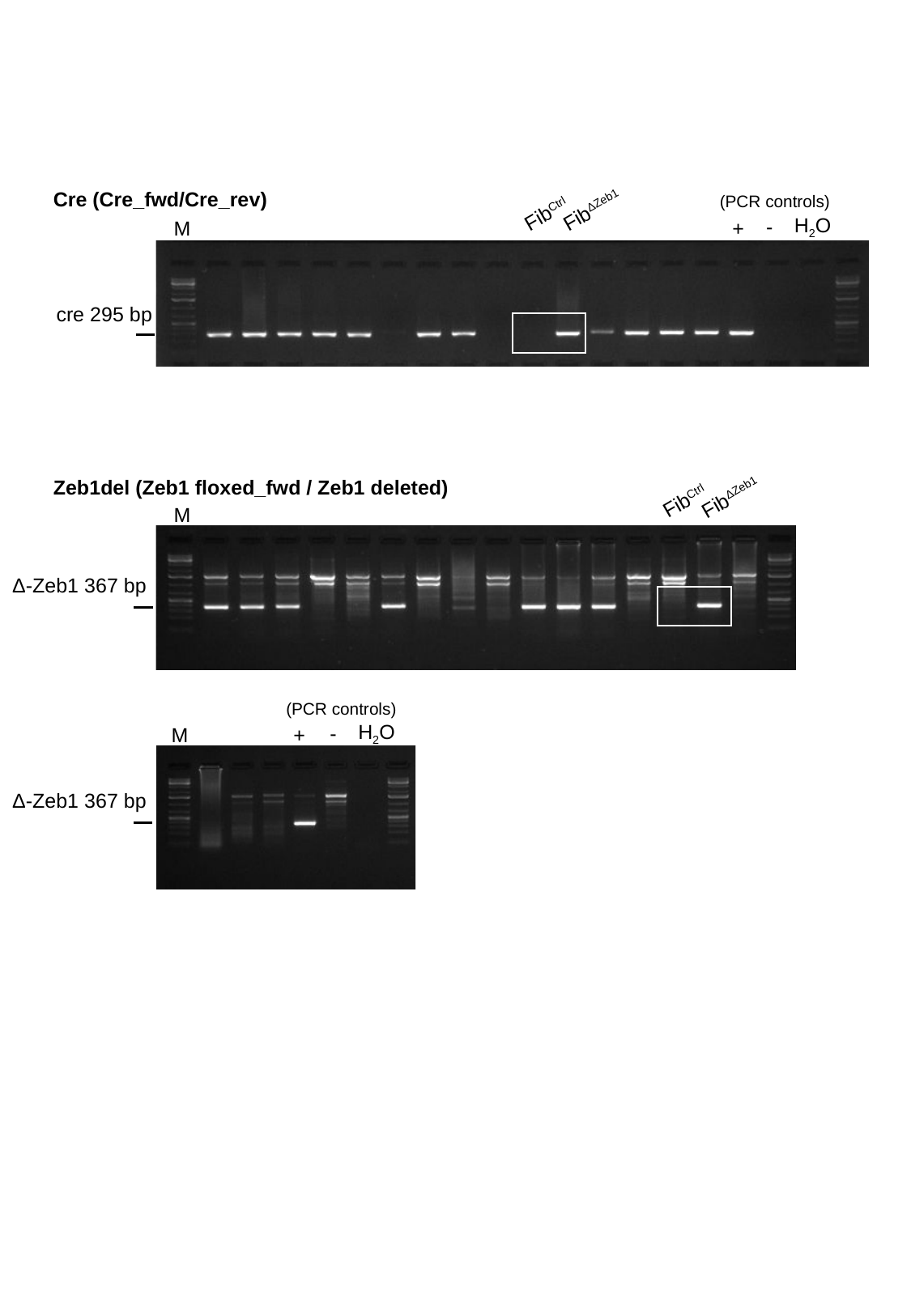

Cre (Cre_fwd/Cre_rev)
(PCR controls)
FibΔZeb1
FibCtrl
-
H2O
M
+
 cre 295 bp
Zeb1del (Zeb1 floxed_fwd / Zeb1 deleted)
FibΔZeb1
FibCtrl
M
Δ-Zeb1 367 bp
(PCR controls)
-
H2O
M
+
Δ-Zeb1 367 bp
Col1Zeb1 (SI)
 3431 3437? 3439? d/+ KPC- H2O
