# Supplementary material for: ZEB1-mediated fibroblast polarization controls inflammation and sensitivity to immunotherapy in colorectal cancer
Source: EMBO Rep. 2024 Jun 27;25(8):15. doi: 10.1038/s44319-024-00186-7 (PMC11315988; doi:10.1038/s44319-024-00186-7)
Supplement: Supplementary file 9 — Expanded View Figures [file 44319_2024_186_MOESM9_ESM.pdf]

Expanded View Figures

Figure EV1. Loss of *Zeb1* in fibroblasts does not affect morphology of primary tumors in the orthotopic transplantation model.

(**A**) IF images and quantification of ZEB1 expression in tumor stroma after orthotopic transplantation of AKP tumor organoids ($n = 10/9$ independent mice for $Fib^{Ctrl}/Fib^{\Delta Zeb1}$, $P < 0.0001$, Student's t test). (**B**) Quantification of primary tumor engraftment in treatment-naïve Fib^{Ctrl} and $Fib^{\Delta Zeb1}$ mice after orthotopic transplantation of AKP tumor organoids. Numbers of experimental mice are indicated. (**C**) Tumor volume after orthotopic transplantation of AKP tumor organoids ($n = 8/5$ independent mice for $Fib^{Ctrl}/Fib^{\Delta Zeb1}$, $P = 0.7584$, Student's t test). (**D**, **E**) Representative H&E stainings of AKP (**D**) and AKP^{re} (**E**) tumor sections. Top left corners show higher magnification of the indicated regions. (**F–H**) Analysis of tumors after orthotopic transplantation of AKP^{re} tumor organoids in treatment-naïve Fib^{Ctrl} and $Fib^{\Delta Zeb1}$ mice. (**F**) Tumor onset ($n = 4/5$ for $Fib^{Ctrl}/Fib^{\Delta Zeb1}$; $P = 0.6401$, Mantel–Cox test). (**G**) Quantification of tumor engraftment. Numbers of experimental mice are indicated. (**H**) Tumor volume ($n = 4/4$ independent mice for $Fib^{Ctrl}/Fib^{\Delta Zeb1}$). (**I–K**) Analysis of tumors after orthotopic transplantation of AKP^{re} tumor organoids in control IgG-treated Fib^{Ctrl} and $Fib^{\Delta Zeb1}$ mice. These mice are shown again as controls in Fig. 5. (**I**, **J**) Tumor onset (**I**) and quantification (**J**) after orthotopic transplantation of AKP^{re} tumor organoids ($n = 12/14$ for $Fib^{Ctrl}/Fib^{\Delta Zeb1}$, $P = 0.4564$, Mantel–Cox test). (**K**) Tumor volumes ($n = 8/10$ independent mice for $Fib^{Ctrl}/Fib^{\Delta Zeb1}$). Only tumors collected after day 28 were included. Data information: Data are represented as mean ± SD (**A**, **C**, **H**, **K**). Scale bars represent 50 µm (**A**) or 1 mm (**D**, **E**). Source data are available online for this figure.

© The Author(s)

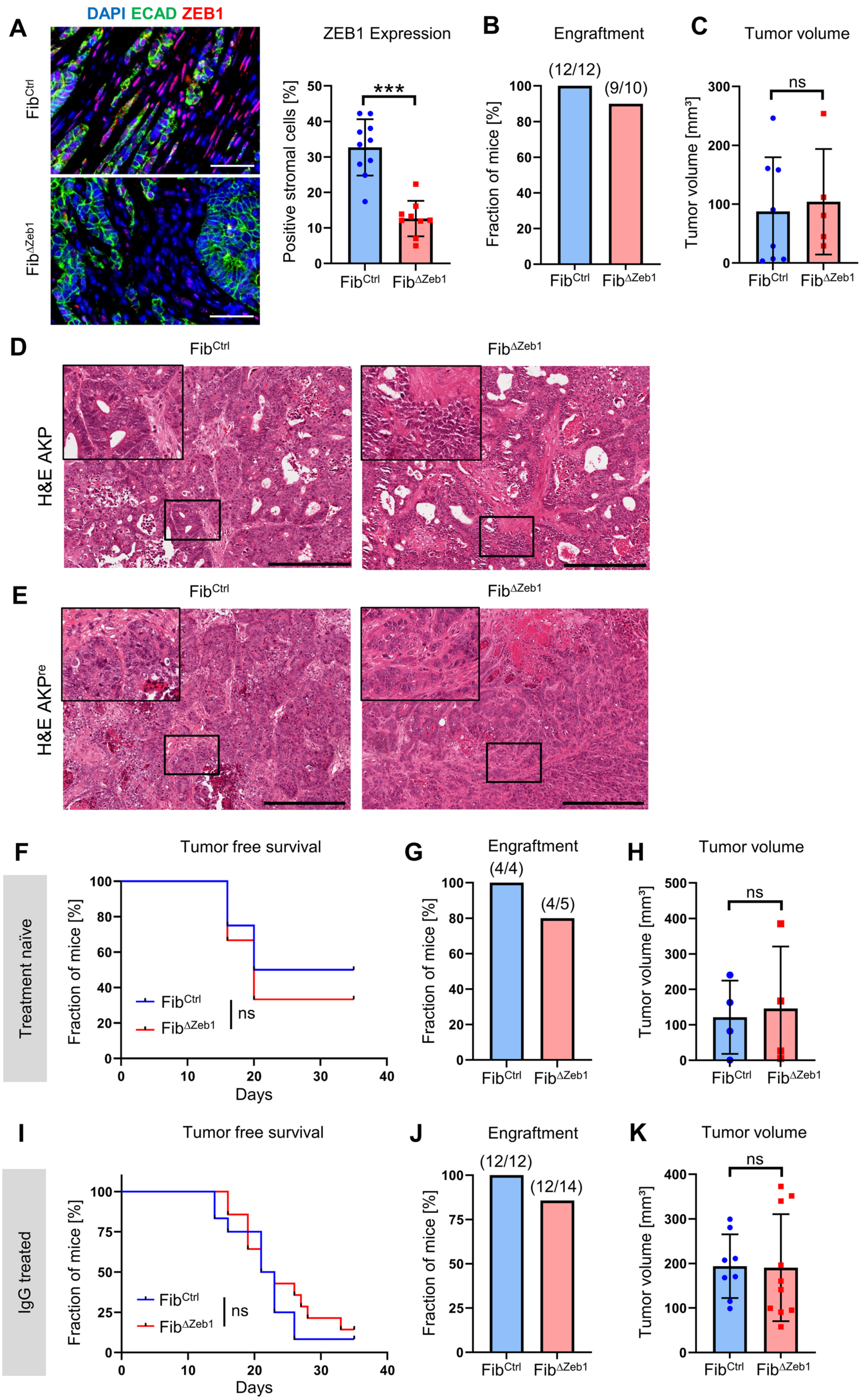
A
DAPI ECAD ZEB1
Fib^Ctrl
Fib^ΔZeb1
ZEB1 Expression
Positive stromal cells [%]
***
B
Engraftment
(12/12)
(9/10)
Fraction of mice [%]
C
Tumor volume
ns
Tumor volume [mm³]
D
H&E AKP
E
H&E AKPre
F
Treatment naïve
Tumor free survival
Fraction of mice [%]
Days
ns
G
Engraftment
(4/4)
(4/5)
H
Tumor volume
I
IgG treated
Tumor free survival
J
Engraftment
(12/12)
(12/14)
K
Tumor volume

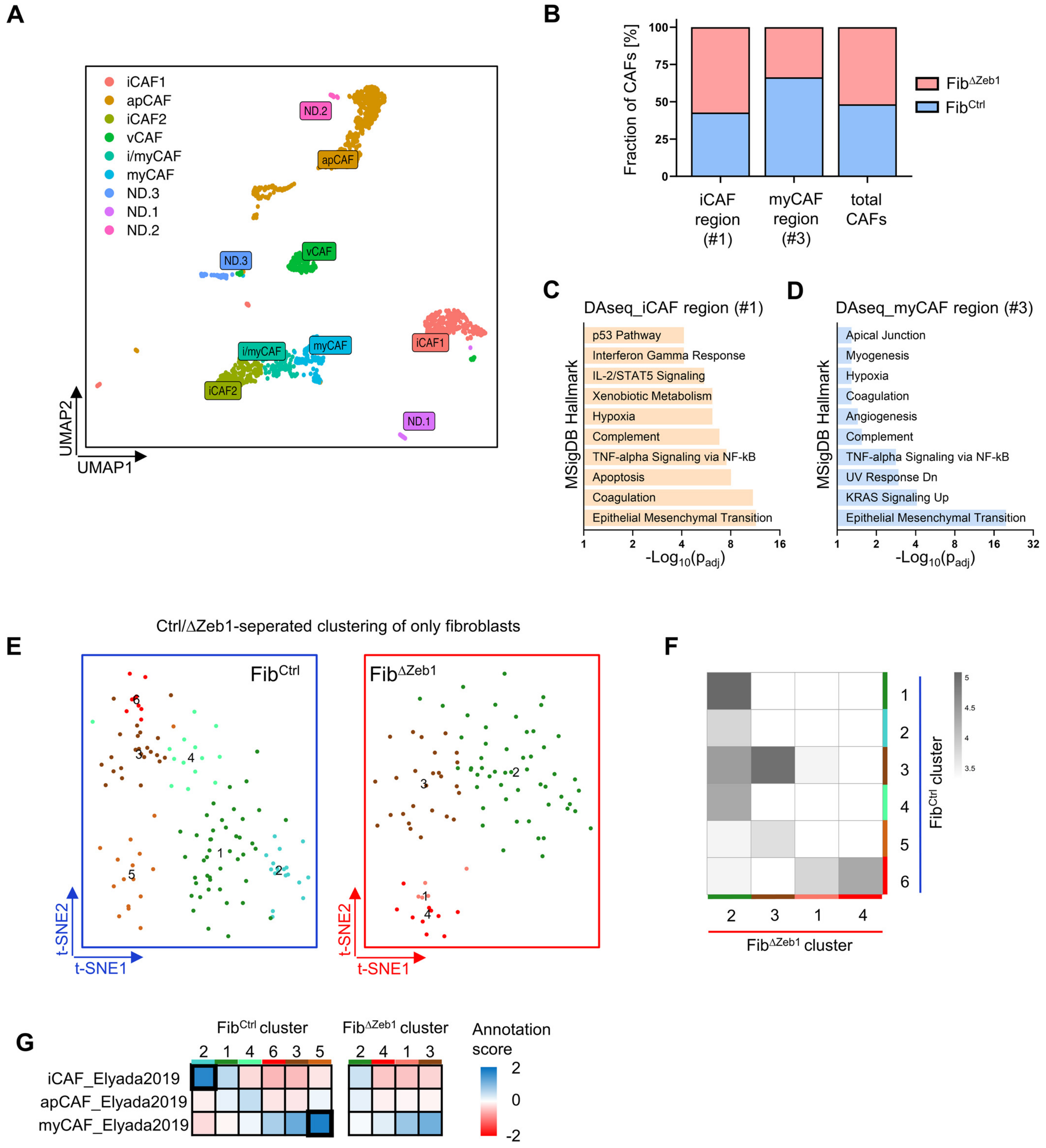
A
iCAF1
apCAF
iCAF2
vCAF
i/myCAF
myCAF
ND.3
ND.1
ND.2
UMAP1
UMAP2
B
Fraction of CAFs [%]
100
75
50
25
0
iCAF region (#1)
myCAF region (#3)
total CAFs
Fib^ΔZeb1
Fib^Ctrl
C
DAseq_iCAF region (#1)
MSigDB Hallmark
p53 Pathway
Interferon Gamma Response
IL-2/STAT5 Signaling
Xenobiotic Metabolism
Hypoxia
Complement
TNF-alpha Signaling via NF-kB
Apoptosis
Coagulation
Epithelial Mesenchymal Transition
-Log10(padj)
D
DAseq_myCAF region (#3)
Apical Junction
Myogenesis
Hypoxia
Coagulation
Angiogenesis
Complement
TNF-alpha Signaling via NF-kB
UV Response Dn
KRAS Signaling Up
Epithelial Mesenchymal Transition
E
Ctrl/ΔZeb1-seperated clustering of only fibroblasts
Fib^Ctrl
Fib^ΔZeb1
t-SNE1
t-SNE2
F
Fib^Ctrl cluster
Fib^ΔZeb1 cluster
G
Fib^Ctrl cluster
Fib^ΔZeb1 cluster
Annotation score
iCAF_Elyada2019
apCAF_Elyada2019
myCAF_Elyada2019

 © The Author(s)

Figure EV2. scRNA-seq in AOM/DSS and orthotopic models show reduced CAF diversity and impaired subtype-specific gene expression in Fib$^{\Delta Zeb1}$ tumors.

(**A**) Phenotypic annotation of AOM/DSS CAFs ($n = 3$ mice per genotype; corresponding to Fig. 2) upon integrated clustering by scoring of iCAF/myCAF/apCAF/mCAF/vCAF gene signatures (Bartoschek et al, 2018; Elyada et al, 2019) and displayed on UMAP Leiden clusters, pooled according to the determined scores. Note that 'i/myCAFs' share features of iCAFs and myCAFs and that the identities of 3 cell clusters could not be determined (ND) using these gene sets. (**B**) Genotype distribution of cells in AOM/DSS CAFs in DA regions (please refer to Fig. 2). The fraction of Fib^{Ctrl} and Fib$^{\Delta Zeb1}$ CAFs in each DA region or among all CAFs is shown. (**C**, **D**) Gene set enrichment analysis using Enrichr of marker genes from DAseq region 1 (**C**) and 3 (**D**) as compared to all other CAFs (Benjamini-Hochburg corrected Fisher's exact test). (**E**) t-SNE sub-clustering of fibroblasts separately in Fib^{Ctrl} (left) and Fib$^{\Delta Zeb1}$ mice (right) of the orthotopic model showing less clusters in Fib$^{\Delta Zeb1}$. (**F**) Cluster similarities defined by cluster annotations based on 'SingleR' scores (see "Methods" for details) (Aran et al, 2019). Grayscale shows the log_2-transformed number of cells across clusters. Note the low similarity of Fib$^{\Delta Zeb1}$ cells with Fib^{Ctrl} clusters 2 and 5. (**G**) Heatmap showing the similarity (annotation scores) of gene expression in CAF clusters with published gene sets. Note, the high scores of Fib^{Ctrl} clusters 2 and 5 when compared with 'iCAF' and 'myCAF' signatures, respectively, and absence in Fib$^{\Delta Zeb1}$. Source data are available online for this figure.

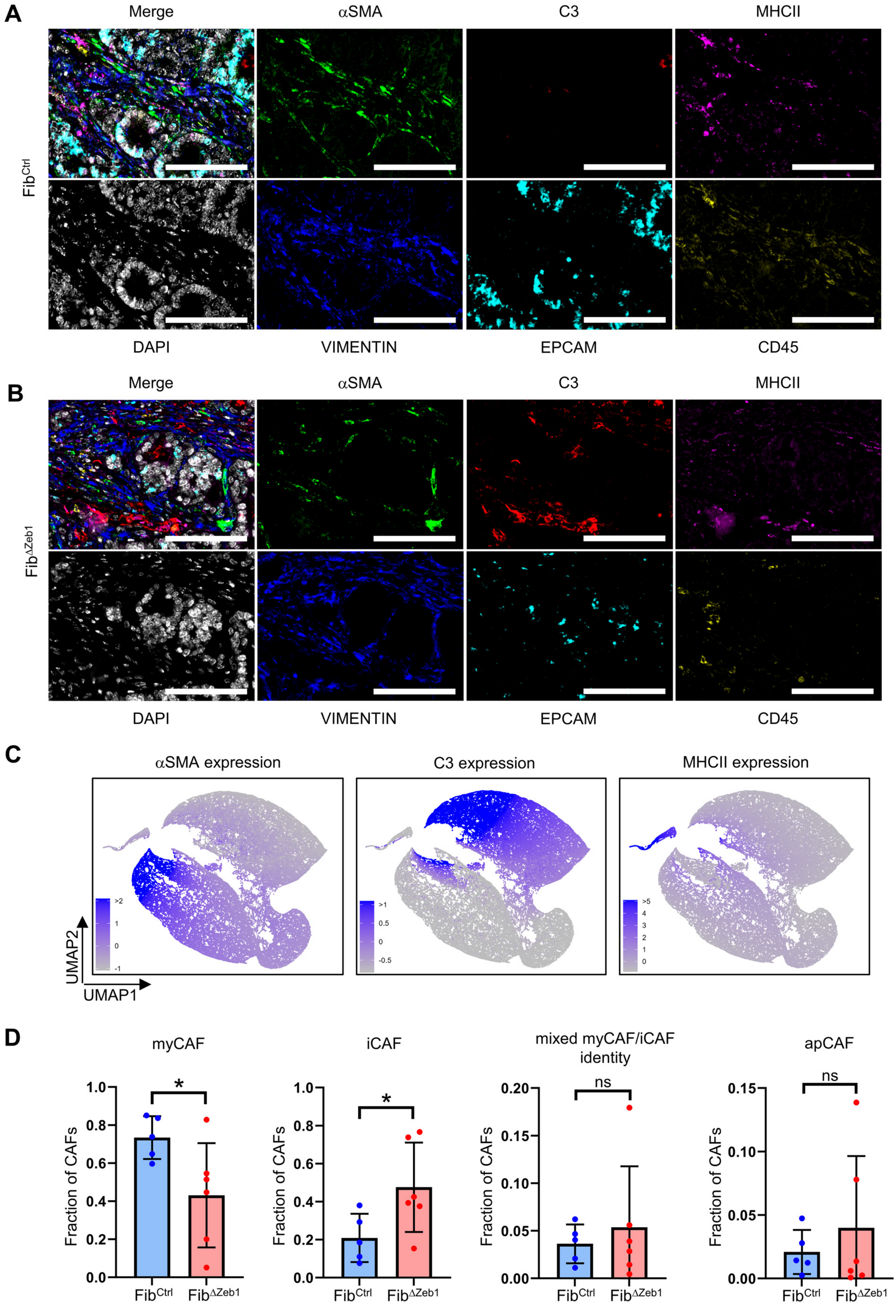
A
Merge
αSMA
C3
MHCII
Fib^Ctrl
DAPI
VIMENTIN
EPCAM
CD45
B
Merge
αSMA
C3
MHCII
Fib^ΔZeb1
DAPI
VIMENTIN
EPCAM
CD45
C
αSMA expression
C3 expression
MHCII expression
UMAP2
UMAP1
D
myCAF
iCAF
mixed myCAF/iCAF identity
apCAF
Fraction of CAFs
*
*
ns
ns
Fib^Ctrl
Fib^ΔZeb1

© The Author(s)

Figure EV3. Multiplexed IF staining of tumor sections reveals an enrichment of iCAF-like cells in Fib$^{\Delta Zeb1}$ tumors.

(**A**, **B**) Single marker images (VIM, αSMA, C3, MHCII, EPCAM, CD45, DAPI) and merge of all channels for the representative images of Fib^{Ctrl} (**A**) and Fib$^{\Delta Zeb1}$ (**B**) orthotopic tumors shown in Fig. 2K. (**C**) Normalized staining intensities of CAF markers (αSMA, C3, MHCII) in UMAP embedding of CAFs from Fib^{Ctrl} and Fib$^{\Delta Zeb1}$ tumors. (**D**) Quantification of the distribution of CAF subtypes based on thresholds for αSMA (myCAF-like), C3 (iCAF-like) or MHCII (apCAF) staining intensity ($n = 5/6$ independent mice for Fib^{Ctrl}/Fib$^{\Delta Zeb1}$, myCAF: $P = 0.0469$, iCAF: $P = 0.0499$, Student's t test). Data information: Data are presented as mean ± SD (**D**). Scale bars represent 100 μm (**A**, **B**). Source data are available online for this figure.

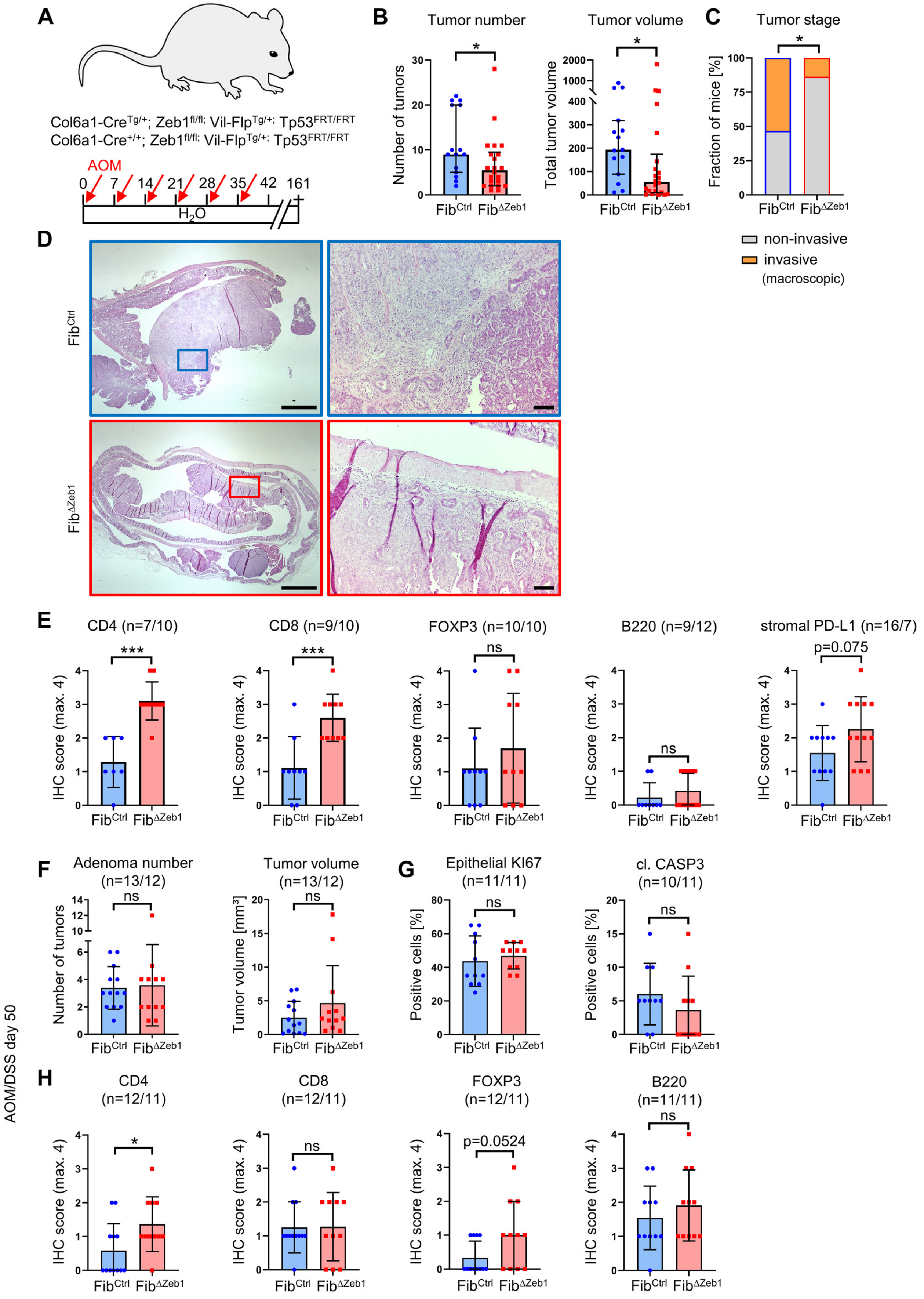
A
Col6a1-CreTg/+; Zeb1fl/fl; Vil-FlpTg/+; Tp53FRT/FRT
Col6a1-Cre+/+; Zeb1fl/fl; Vil-FlpTg/+; Tp53FRT/FRT
AOM
0 7 14 21 28 35 42 161
H2O
B
Tumor number
Number of tumors
Tumor volume
Total tumor volume
C
Tumor stage
Fraction of mice [%]
non-invasive
invasive
(macroscopic)
FibCtrl
FibΔZeb1
D
E
CD4 (n=7/10)
CD8 (n=9/10)
FOXP3 (n=10/10)
B220 (n=9/12)
stromal PD-L1 (n=16/7)
p=0.075
IHC score (max. 4)
ns
F
Adenoma number (n=13/12)
Tumor volume (n=13/12)
Tumor volume [mm3]
G
Epithelial KI67 (n=11/11)
cl. CASP3 (n=10/11)
Positive cells [%]
AOM/DSS day 50
H
CD4 (n=12/11)
CD8 (n=12/11)
FOXP3 (n=12/11)
p=0.0524
B220 (n=11/11)

 © The Author(s)

Figure EV4. Loss of *Zeb1* in fibroblasts impairs tumor progression and increases T-cell infiltration in the invasive non-inflammation-driven AOM/p53 model.

(**A**) Schematic representation of the AOM/p53 model. (**B**) Quantification of numbers and volumes of tumors in the colons of Fib^{Ctrl} and $Fib^{\Delta Zeb1}$ mice at the endpoint ($n = 15/22$ for Fib^{Ctrl}/ $Fib^{\Delta Zeb1}$, number: $P = 0.0433$, volume: $P = 0.0202$, Mann–Whitney test). (**C**) Macroscopic evaluation of the most advanced/progressed tumor per mouse categorizing 'T4' as fully invasive (penetrating the muscle) or not ('T1-T3') (fraction of mice is given, $n = 15/22$ for Fib^{Ctrl}/$Fib^{\Delta Zeb1}$, $P = 0.0252$, Fisher's exact test). (**D**) Representative H&E stainings with a higher magnification of the indicated region to the right. (**E**) IHC-based quantification of immune cell infiltration and stromal PD-L1 expression of tumors from Fib^{Ctrl} and $Fib^{\Delta Zeb1}$ mice. Numbers of experimental mice per genotype are indicated (CD4, CD8, FOXP3, B220, PD-L1: $P < 0.0001$, $P = 0.0010$, 0.3618, 0.3748, 0.0745, Student's *t* test). (**F–H**) AOM/DSS model until day 50 in Fib^{Ctrl} and $Fib^{\Delta Zeb1}$ mice. Macroscopic analysis of early adenomas (number: $P = 0.8339$, volume: $P = 0.2088$, Student's *t* test) (**F**) and IHC quantification of epithelial proliferation and cell death (KI67: $P = 0.5403$, cl. CASP3: $P = 0.2773$, Student's *t* test) (**G**), as well as immune cell infiltration (**H**). Numbers of experimental mice per genotype are indicated (CD4, CD8, FOXP3, B220: $P = 0.0296$, 0.9515, 0.0524, 0.3996, Student's *t* test). Data information: Data are presented as mean ± SD (**B**, **E–H**). Scale bars represent 1.5 mm (**D**, left) or 100 µm (**D**, right). Source data are available online for this figure.

© The Author(s)

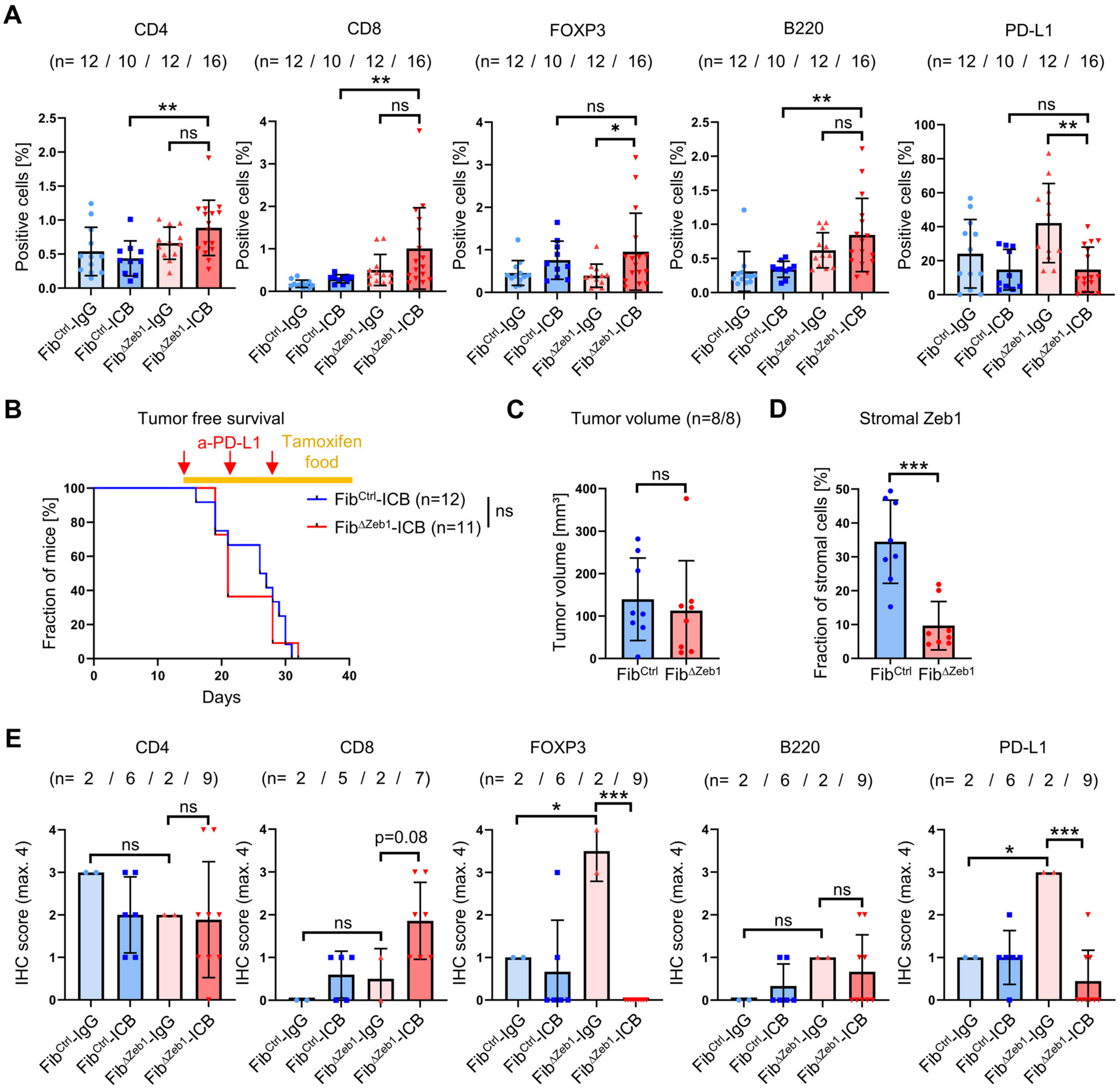

Figure EV5. Monitoring of immune cell infiltration after ICB and effect of late-stage deletion of *Zeb1* on ICB.

(**A**) IHC-based quantification of immune cell infiltration and PD-L1 expression in tumors from Fib^{Ctrl} and $Fib^{\Delta Zeb1}$ mice after orthotopic transplantation of AKP^{re} organoids and intraperitoneal injection of a-PD-L1 antibodies or control IgGs. Numbers of experimental mice per condition are indicated (CD4, CD8, FOXP3, B220, PD-L1: $P = 0.0032$, 0.0076, 0.6442, 0.0027, >0.9999 (Fib^{Ctrl}-ICB vs $Fib^{\Delta Zeb1}$-ICB), p = 0.1568, 0.0525, 0.0304, 0.2150, 0.0004 ($Fib^{\Delta Zeb1}$-IgG vs $Fib^{\Delta Zeb1}$-ICB), Šídák's multiple comparisons test). IgG-treated mice contributed to initial AKP^{re} analysis (Fig. 1). (**B**) Kaplan–Meier analysis showing tumor-free survival of Fib^{Ctrl} and $Fib^{\Delta Zeb1}$ mice after orthotopic transplantation of AKP^{re} organoids and intraperitoneal injection of a-PD-L1 antibodies or control IgGs, as indicated by red arrows. Recombination of *Zeb1* was induced by tamoxifen food starting from day (d) 14. Mice were considered tumor-free if no tumor was detected by palpation. Numbers of experimental mice per condition are indicated ($P = 0.7583$, Mantel–Cox test). (**C**) Quantification of tumor volumes after orthotopic transplantation of AKP^{re} tumor organoids and ICB with late recombination of *Zeb1*. Only tumors collected after d28 were included in this analysis ($n = 8$ independent mice, $P = 0.6276$, Student's *t* test). (**D**) IF-based quantification of stromal cells expressing ZEB1 ($n = 8/8$ independent mice for $Fib^{Ctrl}/Fib^{\Delta Zeb1}$, $P = 0.0002$, Student's *t* test). (**E**) IHC-based quantification of immune cell infiltration and PD-L1 expression in AOM/DSS tumors from Fib^{Ctrl} and $Fib^{\Delta Zeb1}$ mice receiving ICB (2 intraperitoneal injections of a-PD-L1 and a-CTLA-4 antibodies or control IgGs per week starting at d70 of AOM/DSS tumorigenesis). Numbers of experimental mice per condition are indicated (CD4, CD8, FOXP3, B220, PD-L1: $P = 0.9902$, 0.0802, <0.0001, 0.0070, 0.0003 ($Fib^{\Delta Zeb1}$-IgG vs $Fib^{\Delta Zeb1}$-ICB), $P = 0.6241$, 0.7615, 0.0070, 0.3163, 0.0144 (Fib^{Ctrl}-IgG vs $Fib^{\Delta Zeb1}$-IgG), Šídák's multiple comparisons test). Data information: Data are presented as mean ± SD (**A**, **C–E**). Source data are available online for this figure.

© The Author(s)
